# Supplementary figures and images for: Transcriptome of nasopharyngeal samples from COVID-19 patients and a comparative analysis with other SARS-CoV-2 infection models reveal disparate host responses against SARS-CoV-2
Source: J Transl Med. 2021 Jan 7;19:32. doi: 10.1186/s12967-020-02695-0 (PMC7790360; doi:10.1186/s12967-020-02695-0)

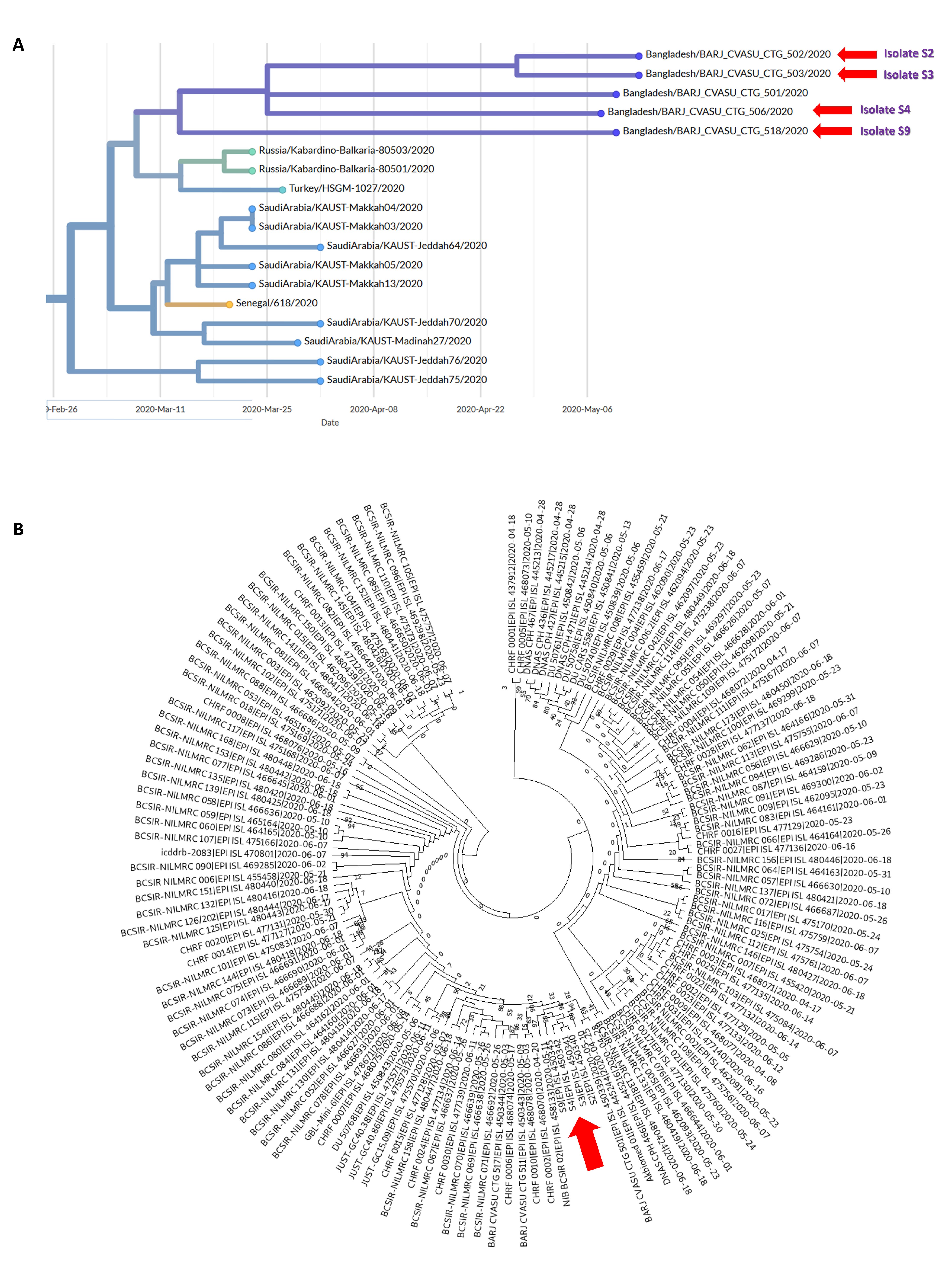

Supplement: Supplementary file 2 — Additional file 2: Figure S1. A. Snapshot of Nextstrain data portal showing the phylogenetic relationship of two SARS-CoV-2 isolates used in this study. Isolates of this study are indicated using a red arrow. B. Phylogenetic tree of Bangladeshi SARS-CoV-2 isolates. Neighbor-joining tree using MEGA tools. Isolates reported in this study are indicated with a red arrow. The evolutionary history was inferred using the Neighbor-Joining method. The optimal tree with the sum of branch length = 0.01403419 is shown. The percentage of replicate trees in which the associated taxa clustered together in the bootstrap test (500 replicates) are shown next to the branches. The evolutionary distances were computed using the Maximum Composite Likelihood method and are in the units of the number of base substitutions per site. This analysis involved 145 nucleotide sequences. Codon positions included were 1st + 2nd + 3rd + Noncoding. All positions with less than 95% site coverage were eliminated, i.e., fewer than 5% alignment gaps, missing data, and ambiguous bases were allowed at any position (partial deletion option). There was a total of 29827 positions in the final dataset. Values represent bootstrap numbers (%). [file 12967_2020_2695_MOESM2_ESM.tif]

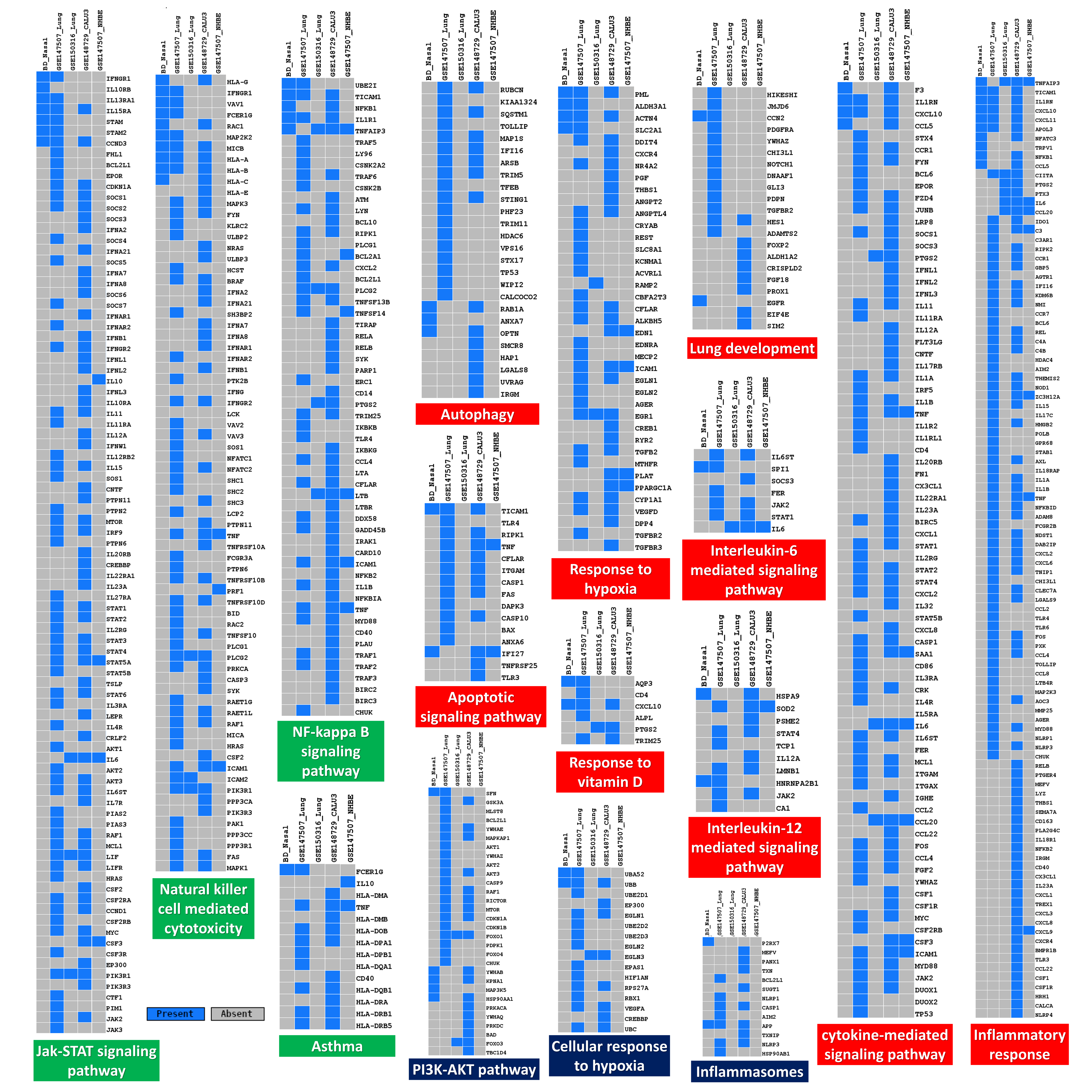

Supplement: Supplementary file 7 — Additional file 7: Figure S3. Deregulated genes of selected terms from Fig. 3 in different SARS-CoV-2 infection systems. For individual processes, blue means presence (differentially expressed gene of the module term) while grey means absence (not differentially expressed in the experimental condition in that module term). Processes in the green, blue, red color background represent KEGG, Bioplanet, GOBP enriched terms, respectively. [file 12967_2020_2695_MOESM7_ESM.tiff]

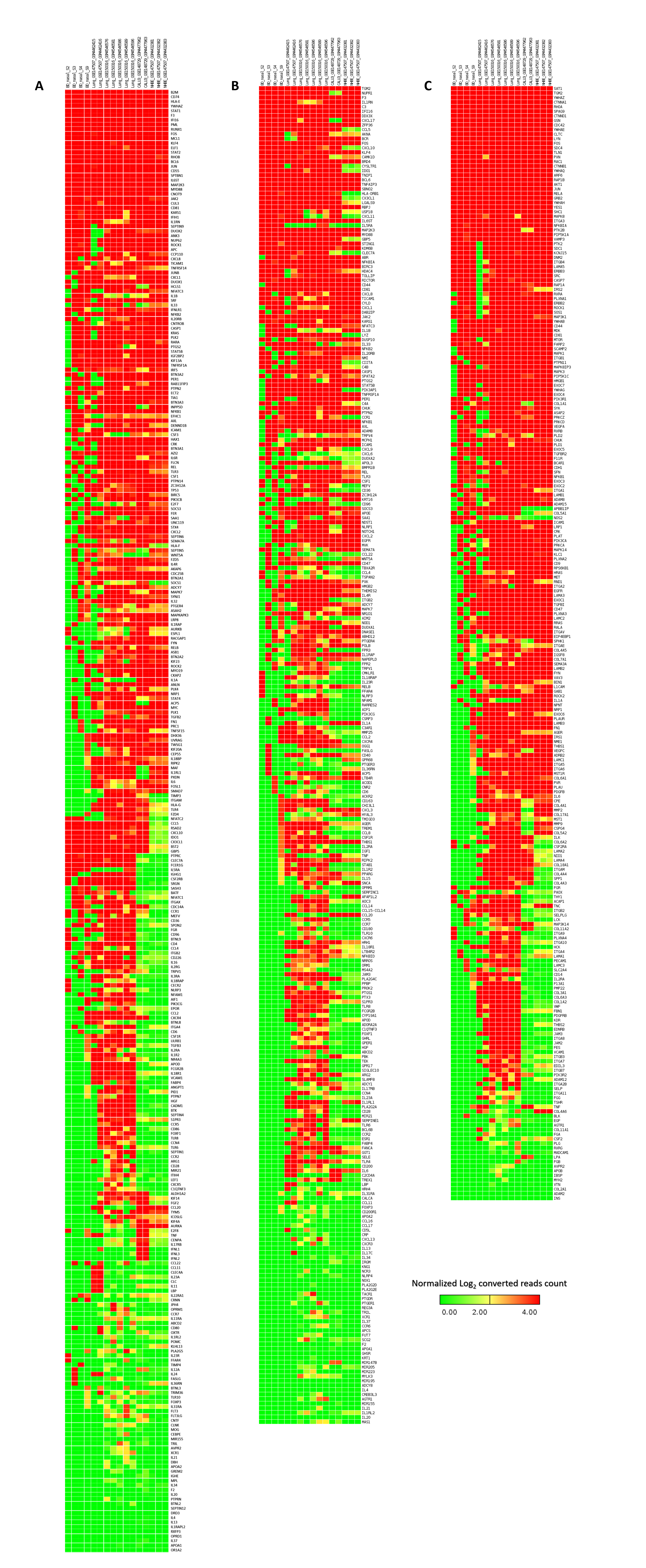

Supplement: Supplementary file 9 — Additional file 9: Figure S4. Expanded view of the heatmaps A, B, C of Fig. 6. [file 12967_2020_2695_MOESM9_ESM.tif]
