# Supplementary material for: Transcriptome of nasopharyngeal samples from COVID-19 patients and a comparative analysis with other SARS-CoV-2 infection models reveal disparate host responses against SARS-CoV-2
Source: J Transl Med. 2021 Jan 7;19:32. doi: 10.1186/s12967-020-02695-0 (PMC7790360; doi:10.1186/s12967-020-02695-0)
Supplement: Supplementary file 3 — Additional file 3. Patient specific clinical features observed in the analyzed four COVID-19 patients. [file 12967_2020_2695_MOESM3_ESM.pdf]

Additional file 3: Patient specific clinical features observed in the analyzed four COVID-19 patients.

Patients' Information

Patient Sample	S2	S3	S4	S9
Sex	Female	Female	Male	Male
Age	43	85	42	55

Patients' Clinical Features

Clinical Features	S2	S3	S4	S9
Fever	Yes	Yes	Yes	Yes
Headache	No	No	No	Yes
Nausea	No	No	No	No
Fatigue	Yes	Yes	No	No
Coughing	Yes	Yes	Yes	Yes
Shortness of Breath	No	Yes	Yes	Yes
Loss of smell and taste	Yes	Yes	No	Yes
Sore throat	Yes	Yes	Yes	Yes
Body ache/pain	Yes	No	No	No
Diarrhea	No	Yes	No	Yes
Hospitalized	No	Yes	No	Yes
Needed Oxygen support	No	Yes (for a short time period- only for 4 hours after hospitalization), oxygen saturation was normal throughout the diseased state	No	Yes
Comorbidities	No	Asthma	NA	NA
Post-disease complications	Itching (might not be related to COVID)	None	NA	NA
Current status of the patients	Healthy and living	Healthy and living	Healthy and living	Dead
Latest COVID-19 test result	Tested COVID-19 negative on June 13	Tested COVID-19 negative on June 13	Tested COVID-19 negative on June 13	NA
