# Supplementary material for: Transcriptome of nasopharyngeal samples from COVID-19 patients and a comparative analysis with other SARS-CoV-2 infection models reveal disparate host responses against SARS-CoV-2
Source: J Transl Med. 2021 Jan 7;19:32. doi: 10.1186/s12967-020-02695-0 (PMC7790360; doi:10.1186/s12967-020-02695-0)
Supplement: Supplementary file 4 — Additional file 4. Differentially expressed genes found in the four nasal samples of COVID-19 patients. [file 12967_2020_2695_MOESM4_ESM.pdf]

Additional file 4 : Differentially expressed genes found in the nasal samples of COVID-19 patients.

Up-regulated	Down-regulated	Differentially expressed
RNA5S14	LINC00963	RNA5S14
RNA5S11	ADGRG1	RNA5S11
RNA5S8	SRSF5	RNA5S8
RNA5S1	ACTN4	RNA5S1
RNA5S10	EWSR1	RNA5S10
RNA5S13	HK1	RNA5S13
RNA5S15	TMED2	RNA5S15
RNA5S2	CCZ1B	RNA5S2
RNA5S16	PSMD2	RNA5S16
RNA5S3	VAMP3	RNA5S3
RNA5S6	CTNND1	RNA5S6
RNA5S17	ARRDC3	RNA5S17
RNA5S7	RDH10	RNA5S7
RNA5S12	EIF3CL	RNA5S12
RNA5S5	PRKAR1A	RNA5S5
RNA5S4	PPP2CB	RNA5S4
RNU1-28P	TSPYL1	RNU1-28P
RNVU1-18	SRP9	RNVU1-18
RNU1-4	SMARCA2	RNU1-4
RNVU1-29	GLUL	RNVU1-29
RNU1-2	RBM25	RNU1-2
RNU1-1	TNFRSF21	RNU1-1
RNU1-27P	SELENBP1	RNU1-27P
RNU1-3	DHCR24	RNU1-3
RNVU1-7	MAT2A	RNVU1-7
RNY1	EIF3C	RNY1
RNU4-2	SCARB2	RNU4-2
RNA5S9	TMPRSS4	RNA5S9
FP671120.2	EIF4G1	FP671120.2
CR392039.1	ENC1	CR392039.1
VTRNA1-1	HNRNPH1	VTRNA1-1
RNA5SP370	WDR1	RNA5SP370
FP236383.6	ADSS2	FP236383.6
RNA5SP202	TMEM123	RNA5SP202
RNVU1-28	IL33	RNVU1-28
CDR1	CCZ1	CDR1
SNORA73B	FBXL5	SNORA73B
RNU1-11P	ZBTB7A	RNU1-11P
RNY4	HNRNPK	RNY4
FP236383.1	PLEKHB2	FP236383.1
RNA5SP389	HNRNPU	RNA5SP389
RNU4-1	ARL8B	RNU4-1
FP236383.9	DHX15	FP236383.9
H4C5	UBQLN1	H4C5
MIR3648-1	ANXA7	MIR3648-1
SMR3B	NDRG2	SMR3B
H4C11	TOMM20	H4C11
RNA5-8SN2	GANAB	RNA5-8SN2
RNA5-8SN3	CTBP2	RNA5-8SN3
FP671120.4	CBWD6	FP671120.4
RNA5-8SN1	ITGB1	RNA5-8SN1
FP236383.3	ASPH	FP236383.3
MIR3648-2	ATP8B1	MIR3648-2
FP671120.1	PFN2	FP671120.1
FP236383.10	DDB1	FP236383.10
RN7SL5P	ARGLU1	RN7SL5P
RNA28S5	SF3B1	RNA28S5
PRB4	ANPEP	PRB4

PRB3	DDX1	PRB3
RNA5SP298	NCSTN	RNA5SP298
FP236383.5	DNAJC10	FP236383.5
FP236383.4	DDX5	FP236383.4
FP236383.12	IFT57	FP236383.12
FP671120.7	PGK1	FP671120.7
RN7SL4P	MIA3	RN7SL4P
RNA5SP226	SDC4	RNA5SP226
RN7SL752P	VPS35	RN7SL752P
RNY3	GALNT7	RNY3
AC079601.1	NAP1L4	AC079601.1
RNA5SP145	SRSF10	RNA5SP145
RNA5SP149	COPB1	RNA5SP149
RNA5SP429	DDX17	RNA5SP429
AL135938.1	RBM39	AL135938.1
RNA5SP74	TAX1BP1	RNA5SP74
CRNN	ANKRD12	CRNN
RNY3P1	LMBRD1	RNY3P1
FAM27E2	CALM2	FAM27E2
FP236383.7	SLC37A3	FP236383.7
RN7SL396P	RDX	RN7SL396P
FP671120.5	ABCD3	FP671120.5
H1-4	CKAP4	H1-4
AL162581.1	TMEM41B	AL162581.1
CRCT1	NFE2L1	CRCT1
RNA5SP335	EPRS1	RNA5SP335
RNA5-8SP6	UBE2Q1	RNA5-8SP6
AC024051.11	ERBB3	AC024051.11
AC024051.5	DIMT1	AC024051.5
AC024051.12	RAC1	AC024051.12
AC024051.1	CALM1	AC024051.1
FP236383.8	PRSS23	FP236383.8
RNU2-1	SFPQ	RNU2-1
AC024051.2	EXOC3	AC024051.2
SNORD17	ADAM9	SNORD17
AC024051.7	XPO1	AC024051.7
AC024051.10	ACSL5	AC024051.10
AC024051.3	CCT2	AC024051.3
AC024051.8	KDELR1	AC024051.8
AC024051.6	PCMTD2	AC024051.6
RNA5SP161	ACTR2	RNA5SP161
AC024051.4	ALDH3A2	AC024051.4
AL161626.1	RTF1	AL161626.1
LINC01783	SH3YL1	LINC01783
CXCL11	ATP6V1A	CXCL11
H4C8	CBX3	H4C8
FABP6-AS1	MAP2K2	FABP6-AS1
RN7SKP71	CLSTN1	RN7SKP71
RN7SKP203	ZDHHC13	RN7SKP203
UBQLNL	LAPTM4A	UBQLNL
CTA-384D8.31	ANXA2	CTA-384D8.31
AC092299.7	SEPTIN2	AC092299.7
BPIFB2	SLC6A6	BPIFB2
SIGLEC16	CLTC	SIGLEC16
TRPV1	STIM2	TRPV1
H2BC8	RCBTB1	H2BC8
FAM27E3	PSMD14	FAM27E3
RNA5SP481	PCYOX1	RNA5SP481
H4C2	SACM1L	H4C2
LINC02176	ST14	LINC02176
MIR663AHG	ACVR1B	MIR663AHG

SCARNA7	SEC61A1	SCARNA7
C1QB	SYNGR2	C1QB
GDF5	IL13RA1	GDF5
SIGLEC1	CAPZA2	SIGLEC1
CA6	XPO7	CA6
SCARNA6	CANX	SCARNA6
MZB1	GALNT1	MZB1
CD8A	SDC1	CD8A
HTN3	MFSD14B	HTN3
AC087276.3	DDX18	AC087276.3
MOCS1	CCNDBP1	MOCS1
AC084082.1	ADGRF1	AC084082.1
RN7SKP80	UBE2N	RN7SKP80
CARNS1	MDFIC	CARNS1
MT-RNR2	SSR1	MT-RNR2
SIGLEC11	CNOT8	SIGLEC11
AP001462.1	SDCBP	AP001462.1
THSD4-AS1	RAB5C	THSD4-AS1
AL354919.1	ERP44	AL354919.1
STATH	MPZL2	STATH
AL137779.1	HMGXB3	AL137779.1
BSN	RSRC2	BSN
CYP2D8P	KPNA1	CYP2D8P
H2AC8	HSPA9	H2AC8
SKAP2	AKR1C3	SKAP2
SNX29P1	ALCAM	SNX29P1
G2E3-AS1	CTNNB1	G2E3-AS1
AC016168.4	GAPDH	AC016168.4
IKZF3	GSN	IKZF3
PPP1R1A	TFCP2L1	PPP1R1A
H2BC4	PDCD4	H2BC4
CHAC1	DYNLT1	CHAC1
AL596325.2	QSOX1	AL596325.2
MAP1LC3C	GOLGA1	MAP1LC3C
HOPX	ESYT2	HOPX
IFIT2	HSP90AA1	IFIT2
RN7SKP255	RBBP9	RN7SKP255
CXCL10	AAGAB	CXCL10
KRT78	CD46	KRT78
AC068987.2	TAP1	AC068987.2
RN7SL391P	HIGD1A	RN7SL391P
ARHGAP40	CLDN7	ARHGAP40
ADRA2A	GM2A	ADRA2A
CNFN	KIF21A	CNFN
TERB1	MARK3	TERB1
TRIM34	PAICS	TRIM34
SLC9C2	NFKB1	SLC9C2
CACNG8	HNRNPA2B1	CACNG8
CLVS1	EIF5B	CLVS1
H1-2	WEE1	H1-2
SPRR2D	CDC42SE2	SPRR2D
SLC23A3	USP48	SLC23A3
AC005921.4	ANKRD36B	AC005921.4
LMX1B	HNRNPL	LMX1B
PGBD5	RCAN3	PGBD5
C8orf34	ALDH3A1	C8orf34
SSPO	SRPRA	SSPO
FLG2	FMO3	FLG2
AP000708.1	CLCN3	AP000708.1
AC006435.4	UGP2	AC006435.4
H2BC5	NIPAL2	H2BC5

FGF17	NCKAP1	FGF17
ABCA8	RAB5A	ABCA8
LINC01562	SLC38A2	LINC01562
FXYD6	EIF1AX	FXYD6
ARHGAP15	DECR1	ARHGAP15
THRB-IT1	SLC25A3	THRB-IT1
LINC01460	SLC44A2	LINC01460
AC073571.1	CP	AC073571.1
MUC5B	PSAP	MUC5B
RN7SL274P	GRN	RN7SL274P
COL9A3	SLC20A1	COL9A3
CROCC2	MARCHF5	CROCC2
AC005696.4	IGFBP3	AC005696.4
ABI3	SON	ABI3
BACH2	GOT1	BACH2
MAL	SLC15A2	MAL
AC091132.5	ACADM	AC091132.5
ZBP1	SLC30A7	ZBP1
AL445665.1	CCT5	AL445665.1
SSUH2	SORT1	SSUH2
RGL4	TMEM87B	RGL4
PPP1R1B	VAV1	PPP1R1B
AP001207.3	PLRG1	AP001207.3
NR1I3	FBP1	NR1I3
POU2F2	EIF2S3	POU2F2
LINC01134	IARS2	LINC01134
AC138866.2	TMBIM6	AC138866.2
AC079949.1	MAOA	AC079949.1
AC138866.1	HSPA5	AC138866.1
IFIT1	B4GALT5	IFIT1
LINC02832	COX6C	LINC02832
CRIP1	CHL1	CRIP1
RBM34	MFSD1	RBM34
MIR99AHG	LINC01578	MIR99AHG
IRF8	BUB3	IRF8
IL15RA	TRA2A	IL15RA
DYSF	ATP5PB	DYSF
AC114498.1	POLR2B	AC114498.1
LINGO4	ACSL3	LINGO4
AC011466.4	SRSF3	AC011466.4
AC015967.2	DHX29	AC015967.2
STAC2	RAB1A	STAC2
TGM3	SCNN1A	TGM3
BCL2L14	WASHC2C	BCL2L14
C10orf82	IMPAD1	C10orf82
AP001020.2	GALNT12	AP001020.2
CDYL2	LAMP2	CDYL2
AC099489.1	ATXN10	AC099489.1
FRMD6-AS1	SPTLC1	FRMD6-AS1
OASL	FDX1	OASL
LETM2	TM4SF1	LETM2
MX2	TMEM50B	MX2
AL691477.1	ATP5F1A	AL691477.1
AL162258.2	RBM6	AL162258.2
PLEKHD1	B2M	PLEKHD1
VASH2	NPC2	VASH2
HERC5	PERP	HERC5
AC004815.1	ELOC	AC004815.1
RNF139-AS1	ANXA3	RNF139-AS1
ZFHX2	SLBP	ZFHX2
CMPK2	TMED10	CMPK2

RUSC2	CPA4	RUSC2
CPED1	EFTUD2	CPED1
ZBTB8A	LDHB	ZBTB8A
ODF3B	MMUT	ODF3B
IFITM1	EEF1A1P6	IFITM1
ECM1	SRI	ECM1
CD53	LINC00511	CD53
IFIT3	SSB	IFIT3
AC016590.1	SKP1	AC016590.1
FCER1G	AKIRIN1	FCER1G
HTN1	DERL1	HTN1
GUSBP3	ATRX	GUSBP3
ZNF324B	UNC5B	ZNF324B
AEN	TMEM30B	AEN
NHLRC4	SLC44A1	NHLRC4
CHDC2	LTA4H	CHDC2
FABP6	SEPHS2	FABP6
H2AC20	ACTG1	H2AC20
ZNF250	IRF2BPL	ZNF250
PTGER2	AQP5	PTGER2
IFITM3	NDUFS8	IFITM3
PLEKHM3	STT3B	PLEKHM3
SMAD9	A4GALT	SMAD9
SPRR2A	TUBA1A	SPRR2A
TBX6	COPS8	TBX6
ZC3H3	NDUFA10	ZC3H3
CD37	PRXL2A	CD37
IFI44L	GDE1	IFI44L
CROCC	DARS1	CROCC
AL031282.2	ANXA1	AL031282.2
PIK3AP1	NUCB2	PIK3AP1
SLC2A5	ID1	SLC2A5
ISG15	FMO2	ISG15
ZNF579	COG2	ZNF579
RSAD2	PSMC5	RSAD2
SYT12	RSRP1	SYT12
AC127164.1	NDFIP1	AC127164.1
AKNA	B4GALT1	AKNA
AC025580.3	RPL6P27	AC025580.3
WDR49	KIFAP3	WDR49
XRRA1	F3	XRRA1
AC027243.1	KARS1	AC027243.1
GUSBP2	OSTC	GUSBP2
ING1	IDH1	ING1
H4C14	JAG1	H4C14
AC020741.1	GLT8D1	AC020741.1
RAMP2-AS1	KYNU	RAMP2-AS1
C1orf229	CMTM6	C1orf229
H4C15	SDR16C5	H4C15
NUPR1	EMB	NUPR1
AC009646.2	CCDC47	AC009646.2
BCDIN3D	PROM1	BCDIN3D
ISG20	APP	ISG20
MT2A	MIPEP	MT2A
CFAP46	C2CD2	CFAP46
MUC5AC	TMEM33	MUC5AC
AC111149.2	CD164	AC111149.2
CHP2	MDH1	CHP2
SPTB	TFCP2	SPTB
CCL5	TMPRSS11D	CCL5
DLEC1	NR2F2	DLEC1

RNF222	VPS26A	RNF222
LBHD1	CAST	LBHD1
NLRC3	GSTK1	NLRC3
IL1RN	YWHAB	IL1RN
P2RX7	TXNDC17	P2RX7
EPHA10	CCN2	EPHA10
PNRC1	CKMT1A	PNRC1
PLPPR2	PSMD11	PLPPR2
FER1L5	PKM	FER1L5
MAPK12	METTL21A	MAPK12
RP11-706O15.5	LAPTM4B	RP11-706O15.5
AL592211.1	PMPCB	AL592211.1
PML	PRNP	PML
PLAAT2	SEC63	PLAAT2
AC138932.1	TMEM165	AC138932.1
CA5A	CAV2	CA5A
USP18	PYGL	USP18
SPI1	ARPC5	SPI1
HNRNPA1P40	TMEM150C	HNRNPA1P40
SPRR2E	CD63	SPRR2E
MCF2L	PLS3	MCF2L
FGR	HLF	FGR
PPP1R16B	AC087473.1	PPP1R16B
CCDC33	ATP6V0D1	CCDC33
ENKD1	PSMB3	ENKD1
MARCKSL1	NOMO1	MARCKSL1
AC026523.2	GTF2E2	AC026523.2
CFAP74	OAT	CFAP74
HMOX1	CD151	HMOX1
GPR65	ALDH2	GPR65
NINL	FBXO3	NINL
AP001107.1	MORN2	AP001107.1
MICB	CTBS	MICB
OAS2	STOM	OAS2
UBE2T	RNF149	UBE2T
MAP4K2	MAP3K5	MAP4K2
RPL37	ERLIN1	RPL37
IRF7	ERMP1	IRF7
NTAN1	SMIM15	NTAN1
IFI27	CCDC80	IFI27
DTX2P1	HSP90B1	DTX2P1
AC027290.3	CKMT1B	AC027290.3
ABHD8	TSPAN3	ABHD8
UBE2L6	STAM2	UBE2L6
AC019117.3	ABHD5	AC019117.3
TEKT2	MSMO1	TEKT2
AC118344.4	RBM5	AC118344.4
NATD1	TM9SF3	NATD1
BEX2	ATP1B1	BEX2
GNL3L	SARAF	GNL3L
SRCIN1	AMFR	SRCIN1
SAMD9	DYNC2LI1	SAMD9
LINC01551	H3-3A	LINC01551
SGTB	RNF145	SGTB
TNFRSF14-AS1	ERG28	TNFRSF14-AS1
SERPING1	TUBA1C	SERPING1
BICD2	RBM3	BICD2
SRGAP3-AS2	GLB1	SRGAP3-AS2
DUSP5	PGD	DUSP5
PRR29	USP47	PRR29
AC008079.1	TAGLN2	AC008079.1

CD96	RETREG2	CD96
RP11-706O15.3	TMX4	RP11-706O15.3
RN7SL718P	EXOC1	RN7SL718P
AHNAK2	MAT2B	AHNAK2
NIN	VPS25	NIN
OCEL1	NUP107	OCEL1
CYP2F1	ADAM15	CYP2F1
CCND3	ARPC3	CCND3
RPS15	SYPL1	RPS15
MUC13	TGFBR1	MUC13
AKAP12	DSG2	AKAP12
GUSBP1	RPL4P4	GUSBP1
SPINK5	SELENOP	SPINK5
ZNF76	EPCAM	ZNF76
PTCHD4	S100A6	PTCHD4
CRYBG3	COG6	CRYBG3
ZFP36	ANKRD10	ZFP36
CARD16	WDR33	CARD16
DHX35	LRRC8D	DHX35
SAMD9L	MMP14	SAMD9L
ST3GAL2	RWDD4	ST3GAL2
PPDPF	SDF4	PPDPF
EYA1	DLD	EYA1
SP2	FHL2	SP2
CCDC40	SLC35F5	CCDC40
IFIH1	TACSTD2	IFIH1
AMOTL2	ASAH1	AMOTL2
NUMA1	GUF1	NUMA1
FAM222A	ENO1	FAM222A
CCDC88C	LUC7L3	CCDC88C
RND1	MTDH	RND1
UBA52	JKAMP	UBA52
ARHGAP39	H2AZ2	ARHGAP39
WDR62	ERAP1	WDR62
AC134407.2	TMEM192	AC134407.2
SCGB1A1	RCN1	SCGB1A1
ATF3	WASH6P	ATF3
ZNF500	ITGB5	ZNF500
ALPK3	SLC35A2	ALPK3
RP11-589F5.3	TTC19	RP11-589F5.3
TICAM1	OCIAD1	TICAM1
UBAP2	ADAM28	UBAP2
CEP135	P4HB	CEP135
ZNF329	SFN	ZNF329
CCDC78	SLC39A7	CCDC78
C12orf50	EIF4A3	C12orf50
EVI2B	TMEM30A	EVI2B
YY1AP1	GPD2	YY1AP1
TRIM14	CD59	TRIM14
NKX3-1	GTF2H2B	NKX3-1
CCDC159	PON2	CCDC159
JPX	HACD3	JPX
ZNF335	GSS	ZNF335
MRTFA	TUFM	MRTFA
FOXG1	ATP2C1	FOXG1
CCDC106	CACHD1	CCDC106
SYTL5	CA12	SYTL5
COTL1	MBOAT2	COTL1
OAS1	THYN1	OAS1
H2AC6	PDIA6	H2AC6
AC004151.1	LEMD3	AC004151.1

MUC21	RPN1	MUC21
HOOK1	PTTG1IP	HOOK1
H2BC18	PRDX1	H2BC18
OAS3	GAA	OAS3
PREX1	ABCC3	PREX1
CCDC88B	IER3IP1	CCDC88B
FTL	EGFR	FTL
FOSB	ANXA4	FOSB
WNK2	IGFBP2	WNK2
MAST3	SLC27A2	MAST3
EPSTI1	TRAPPC3	EPSTI1
PCNT	OS9	PCNT
NFATC3	SLC18B1	NFATC3
HIGD2A	ALOX15	HIGD2A
KIAA0040	GNS	KIAA0040
C15orf62	RRN3	C15orf62
CEP112	PIGX	CEP112
ARHGAP26	ARL6IP1	ARHGAP26
SGSM1	RPL7AP66	SGSM1
MVB12B	AC004069.1	MVB12B
SPAG9	LGR4	SPAG9
SYNPO	LRG1	SYNPO
PKD1P5	SPARCL1	PKD1P5
SHANK2	TMEM87A	SHANK2
CLEC16A	ALDH1A1	CLEC16A
ELF4	LMAN2	ELF4
NCCRP1	AC007318.1	NCCRP1
H2AC18	DDOST	H2AC18
HELB	CHPF	HELB
FCGR2A	CTSB	FCGR2A
ATP5F1E	RASA1	ATP5F1E
SIPA1L3	SLC2A1	SIPA1L3
H2AC19	CFH	H2AC19
SEC24A	TM9SF2	SEC24A
POLR2M	EML3	POLR2M
GNG5	CTSH	GNG5
KAT2B	HNRNPA1P4	KAT2B
A2ML1	ERLIN2	A2ML1
TNFAIP3	MTRNR2L9	TNFAIP3
HSF1	LRP5	HSF1
MFN1	SEMA3A	MFN1
AC126755.1	GFM2	AC126755.1
ALMS1	HNRNPA1L2	ALMS1
FBXO48	PIGG	FBXO48
RABIF	RRM1	RABIF
SLC25A23	TSPAN13	SLC25A23
MCUB	TMEM68	MCUB
SOBP	KRT5	SOBP
HELZ2	ACVR1	HELZ2
CBX6	ATP1A1	CBX6
SRCAP	PRODH	SRCAP
TACC2	RPAP2	TACC2
FAM193A	MFSD11	FAM193A
SERPINB8	FDFT1	SERPINB8
PRPF3	MFSD14C	PRPF3
SYTL2	ANKRD36	SYTL2
MBNL1	SGK1	MBNL1
PATL1	NEU1	PATL1
ZMIZ2	CLDN4	ZMIZ2
ZNF358	SCGB2A1	ZNF358
BLZF1	ERLEC1	BLZF1

STK10	EEF1A1P5	STK10
NIBAN1	AL391121.1	NIBAN1
CDK18	POR	CDK18
PHC3	RAB3IP	PHC3
FYCO1	FAM3B	FYCO1
LGALS9B	CALR	LGALS9B
TEX9	AC073333.1	TEX9
MX1	EBPL	MX1
FNDC3B	NPTN	FNDC3B
CLPB	HSD17B13	CLPB
PRRC2A	RPS21	PRRC2A
APOL3	NIFK	APOL3
CENPBD1P1	ALS2CL	CENPBD1P1
RAB8A	PSEN2	RAB8A
DAP	RCN2	DAP
ZNF592	CHKA	ZNF592
GTDC1	GTF2H2C	GTDC1
MOB3A	TMA7	MOB3A
TADA2B	ALDH7A1	TADA2B
SH3KBP1	CD81	SH3KBP1
SAMD4B	RTN4	SAMD4B
TRAF3IP2	DHX36	TRAF3IP2
SPDEF	SNRPA1	SPDEF
CDKN2B	DNAJC1	CDKN2B
RBMS2	HNRNPA1P35	RBMS2
CIZ1	FTH1P10	CIZ1
DUSP3	HNRNPAB	DUSP3
RFX5	FUCA2	RFX5
MAP3K11	ADK	MAP3K11
TENT5C	FMO5	TENT5C
TBC1D15	RTN3	TBC1D15
RPL36AL	ASCC3	RPL36AL
R3HDM2	PSME1	R3HDM2
MLPH	WASH4P	MLPH
S100A11	TFB2M	S100A11
RND3	REEP5	RND3
UBB	TMEM51	UBB
PAK4	WSB1	PAK4
RASSF9	SREK1	RASSF9
ATXN7	RTN3P1	ATXN7
RUNDC1	RARS1	RUNDC1
IQCE	HADH	IQCE
R3HDM1	MBOAT1	R3HDM1
NEK6	PGAP4	NEK6
JADE2	FAAH2	JADE2
TCOF1	SMG1P4	TCOF1
CSNK1G2	AC008810.1	CSNK1G2
TOB2	TLR2	TOB2
ATN1	ZMPSTE24	ATN1
NCOR2	HNRNPA1P10	NCOR2
IL1R1	PAPSS2	IL1R1
SPECC1	AGL	SPECC1
ANKRD17	TMEM9	ANKRD17
BICDL1	TMEM9B	BICDL1
RAB3B	TSPAN1	RAB3B
VPS37B	CLN5	VPS37B
CRY2	TMEM59	CRY2
ACSS1	AL592114.1	ACSS1
WARS1	GUSB	WARS1
OPTN	ANAPC4	OPTN
ZNF609	COMMD7	ZNF609

PDE4DIP	MAP3K6	PDE4DIP
FCHSD2	ANKRD66	FCHSD2
SP3	DSE	SP3
WWC1	GCLC	WWC1
MLXIP	PRSS8	MLXIP
ZFP36L2	CYP4F12	ZFP36L2
SORBS3	ABCE1	SORBS3
SND1	ENPP4	SND1
FOXK1	ABCA5	FOXK1
GTPBP1	GLUD2	GTPBP1
CCDC69	CALM2P2	CCDC69
C6orf132	KLHL42	C6orf132
	PRMT7	LINC00963
	ITGB6	ADGRG1
	SESN1	SRSF5
	FTH1P2	ACTN4
	CAMK2G	EWSR1
	STAM	HK1
	MANF	TMED2
	THOC3	CCZ1B
	MSTO1	PSMD2
	ZPR1	VAMP3
	INPP1	CTNND1
	NOMO2	ARRDC3
	LRRN1	RDH10
	SRSF11	EIF3CL
	SELENOI	PRKAR1A
	TOPORS	PPP2CB
	PLTP	TSPYL1
	SLC39A6	SRP9
	TMEM106C	SMARCA2
	CD47	GLUL
	SORD2P	RBM25
	AL158206.1	TNFRSF21
	KRT10	SELENBP1
	EIF4A1P4	DHCR24
	UNC93B3	MAT2A
	CXADR	EIF3C
	CUEDC1	SCARB2
	IL10RB	TMPRSS4
	YWHAZP4	EIF4G1
	ATP5F1B	ENC1
	PRKAR2B	HNRNPH1
	SMC4	WDR1
	PTDSS1	ADSS2
	COPG2	TMEM123
	GTF2F2	IL33
	LONRF1	CCZ1
	RPL10P9	FBXL5
	HLA-B	ZBTB7A
	RFNG	HNRNPK
	MAN1B1	PLEKHB2
	EIF4HP1	HNRNPU
	H3P36	ARL8B
	LACTB	DHX15
	CNIH1	UBQLN1
	MAPKAPK3	ANXA7
	JPT1	NDRG2
	SMAP1	TOMM20
	CYP3A5	GANAB
	SPTSSA	CTBP2

	TTC29	CBWD6
	C1D	ITGB1
	PA2G4P6	ASPH
	DPAGT1	ATP8B1
	SMARCAD1	PFN2
	RPL7AP6	DDB1
	BX679664.3	ARGLU1
	PLS1	SF3B1
	F11R	ANPEP
	RMDN3	DDX1
	DDAH2	NCSTN
	BCAP29	DNAJC10
	FSCN1	DDX5
	AKAP1	IFT57
	ITFG1	PGK1
	ARVCF	MIA3
	LOXL4	SDC4
	AC090498.1	VPS35
	FAM3D	GALNT7
	HNRNPA1P7	NAP1L4
	HNRNPA1P12	SRSF10
	RHBDL2	COPB1
	MPP7	DDX17
	UBE2E3	RBM39
	SCPEP1	TAX1BP1
	DNAJB11	ANKRD12
	SDHA	LMBRD1
	LAMB3	CALM2
	VARS1	SLC37A3
	NT5DC1	RDX
	PBXIP1	ABCD3
	TAP2	CKAP4
	HLA-F	TMEM41B
	CDH1	NFE2L1
	PRELID1	EPRS1
	UPK1B	UBE2Q1
	PSMD1	ERBB3
	TMTC4	DIMT1
	ITGAV	RAC1
	NUDT12	CALM1
	PGAM4	PRSS23
	RNF26	SFPQ
	CCDC65	EXOC3
	FARSB	ADAM9
	PARP2	XPO1
	VIPR1	ACSL5
	ARL3	CCT2
	ENY2	KDELR1
	SDCBPP3	PCMTD2
	BCAP31	ACTR2
	B4GALT4	ALDH3A2
	RAMAC	RTF1
	C5orf15	SH3YL1
	FTH1P8	ATP6V1A
	MAD2L1BP	CBX3
	RAC1P2	MAP2K2
	NAAA	CLSTN1
	GLULP4	ZDHHC13
	EXOSC8	LAPTM4A
	MT-TY	ANXA2
	BLOC1S4	SEPTIN2

	AC144530.1	SLC6A6
	TMEM45B	CLTC
	ABHD3	STIM2
	HLA-L	RCBTB1
	AC113935.1	PSMD14
	PRPF39	PCYOX1
	CTNNAL1	SACM1L
	VPS35P1	ST14
	HSP90AB2P	ACVR1B
	UNC93B7	SEC61A1
	CHPT1	SYNGR2
	TNC	IL13RA1
	DDX50	CAPZA2
	MLYCD	XPO7
	AQP3	CANX
	LRRCC1	GALNT1
	TPBG	SDC1
	GJB3	MFSD14B
	PABPC1P4	DDX18
	SLC25A17	CCNDBP1
	SLC44A3	ADGRF1
	SLC26A4	UBE2N
	PRDX4	MDFIC
	GGH	SSR1
	HNRNPA1P8	CNOT8
	LSAMP	SDCBP
	CDS2	RAB5C
	FAT1	ERP44
	ATP6AP2	MPZL2
	MRAP2	HMGXB3
	G6PC3	RSRC2
	ERO1B	KPNA1
	CLK1	HSPA9
	SNX14	AKR1C3
	PTGES3P3	ALCAM
	CCT6A	CTNNB1
	AC006511.4	GAPDH
	KLF9	GSN
	FKBP1C	TFCP2L1
	PIPSL	PDCD4
	FAM162A	DYNLT1
	AC024293.1	QSOX1
	TMEM147	GOLGA1
	ALG1	ESYT2
	CCDC59	HSP90AA1
	DBP	RBBP9
	TF	AAGAB
	FKBP9	CD46
	RPL14P1	TAP1
	TMEM43	HIGD1A
	ACAD10	CLDN7
	BORCS5	GM2A
	EMC7	KIF21A
	ACTA1	MARK3
	UNC93B6	PAICS
	EDEM2	NFKB1
	PTK7	HNRNPA2B1
	ARSDP1	EIF5B
	TMED7	WEE1
	TUSC3	CDC42SE2
	PGRMC1	USP48

	UBLCP1	ANKRD36B
	AC026271.1	HNRNPL
	BMI1	RCAN3
	DYNC1I2P1	ALDH3A1
	ARMT1	SRPRA
	TLR1	FMO3
	GPC1	CLCN3
	VDAC1P1	UGP2
	HNRNPKP4	NIPAL2
	RRAGA	NCKAP1
	KIT	RAB5A
	ST13P3	SLC38A2
	AC136632.1	EIF1AX
	AL391244.2	DECR1
	ETHE1	SLC25A3
	TMED1	SLC44A2
	RPL7P10	CP
	HEXB	PSAP
	MOSPD1	GRN
	SLC52A2	SLC20A1
	KDSR	MARCHF5
	SELENOS	IGFBP3
	HLA-A	SON
	ADH1A	GOT1
	RPL22P1	SLC15A2
	DNAJB9	ACADM
	AC083873.1	SLC30A7
	DENND10P1	CCT5
	FUCA1	SORT1
	HSPA8P5	TMEM87B
	LPCAT3	VAV1
	SLC35B2	PLRG1
	EXOSC9	FBP1
	TAGLN2P1	EIF2S3
	ATP5MC3	IARS2
	EIF3FP3	TMBIM6
	COL6A3	MAOA
	CHCHD1	HSPA5
	TMCO1	B4GALT5
	PABPC3	COX6C
	TP63	CHL1
	CTSC	MFSD1
	HSD17B12	LINC01578
	CEACAM3	BUB3
	SMG1P1	TRA2A
	PSMC1P1	ATP5PB
	ELMO3	POLR2B
	AL158801.6	ACSL3
	UQCRC1	SRSF3
	HLA-V	DHX29
	MGST1	RAB1A
	NTS	SCNN1A
	AL133477.1	WASHC2C
	H3P44	IMPAD1
	PSENEN	GALNT12
	RPL7AP34	LAMP2
	PSPC1	ATXN10
	LAMC1	SPTLC1
	AL121769.1	FDX1
	NDUFA12	TM4SF1
	MT-TL1	TMEM50B

	AC106795.1	ATP5F1A
	AC022968.1	RBM6
	LRRIQ1	B2M
	LRRC17	NPC2
	UFD1	PERP
	UXS1	ELOC
	AC008065.1	ANXA3
	KRT6B	SLBP
	HMGN2P17	TMED10
	ALOX15P1	CPA4
	RPL37AP1	EFTUD2
	ATP6AP1	LDHB
	AC099670.1	MMUT
	BMP3	EEF1A1P6
	FKSG70	SRI
	FAM171A1	LINC00511
	RPN2	SSB
	HLA-H	SKP1
	EEF1A1P38	AKIRIN1
	THNSL2	DERL1
	PDIA3	ATRX
	AC064799.1	UNC5B
	KTN1	TMEM30B
	AC209007.1	SLC44A1
	AC104619.3	LTA4H
	SYT8	SEPHS2
	COQ5	ACTG1
	MSH2	IRF2BPL
	NPC1	AQP5
	UQCRFS1P1	NDUFS8
	MORF4L1P1	STT3B
	UBBP4	A4GALT
	GPR89A	TUBA1A
	MFSD5	COPS8
	SERPINB4	NDUFA10
	HNRNPA1P48	PRXL2A
	AP000936.3	GDE1
	SIRT3	DARS1
	AC092115.2	ANXA1
	RPS27P29	NUCB2
	CYP26A1	ID1
	ATP13A5	FMO2
	AL109918.1	COG2
	PIGO	PSMC5
	ITM2B	RSRP1
	RPL9P32	NDFIP1
	PFN1P1	B4GALT1
	EIF2S2P4	RPL6P27
	HNRNPA3P6	KIFAP3
	HSP90AB3P	F3
	PLLP	KARS1
	CLDN1	OSTC
	ANXA8L1	IDH1
	RPL23P8	JAG1
	AC113404.3	GLT8D1
	HSP90AA6P	KYNU
	RPL7P47	CMTM6
	SETP20	SDR16C5
	PHF14	EMB
	SRD5A3	CCDC47
	SCAMP3	PROM1

	AC244034.1	APP
	TRIAP1	MIPEP
	F2RL1	C2CD2
	PPIAP87	TMEM33
	AP002784.2	CD164
	SUMO2P1	MDH1
	AC005000.1	TFCP2
	HLA-C	TMPRSS11D
	MTATP8P1	NR2F2
	KRT18P16	VPS26A
	SERBP1P5	CAST
	RPL4P5	GSTK1
	DCAF13	YWHAB
	SRSF2	TXNDC17
	SETSIP	CCN2
	FKBP9P1	CKMT1A
	PRCP	PSMD11
	CD9	PKM
	IFNGR1	METTL21A
	RPL7P9	LAPTM4B
	TMEM212	PMPCB
	EIF4BP3	PRNP
	PSCA	SEC63
	AL049597.1	TMEM165
	RPL7P32	CAV2
	YWHAZP5	PYGL
	AC012085.1	ARPC5
	HNRNPCP2	TMEM150C
	FTLP3	CD63
	APLP2	PLS3
	SETP14	HLF
	UBE2I	AC087473.1
	CDC42P6	ATP6V0D1
	ANXA8	PSMB3
	AL354702.1	NOMO1
	FGFR3	GTF2E2
	H3-5	OAT
	TSPAN6	CD151
	EIF4A1P2	ALDH2
	RPL7P1	FBXO3
	GAPDHP65	MORN2
	AC105250.1	CTBS
	EPHA1	STOM
	AC002075.2	RNF149
	RPS7P10	MAP3K5
	EEF1A1P19	ERLIN1
	EIF5AL1	ERMP1
	RPL10AP2	SMIM15
	ARSD	CCDC80
	XRCC6P2	HSP90B1
	RPL13AP20	CKMT1B
	AC092597.1	TSPAN3
	EIF4BP6	STAM2
	AC099560.2	ABHD5
	RPL12P38	MSMO1
	RPS26P6	RBM5
	LDHBP2	TM9SF3
	KRT18P11	ATP1B1
	AC004057.1	SARAF
	RPL34P26	AMFR
	BZW1P2	DYNC2LI1

	AC092683.1	H3-3A
	S100A4	RNF145
	HLA-DRB6	ERG28
	C1GALT1C1	TUBA1C
	RPS26P31	RBM3
	SLC5A8	GLB1
	MTCO1P40	PGD
	GAPDHP44	USP47
	RPL7AP11	TAGLN2
	PPIC	RETREG2
	TMX1	TMX4
	ITGA6	EXOC1
	GAPDHP61	MAT2B
	PSMC1P5	VPS25
	EIF4BP7	NUP107
	PPIAP16	ADAM15
	EEF1A1P7	ARPC3
	RPSAP19	SYPL1
	AC005480.2	TGFBR1
	UNC50	DSG2
	MIR22HG	RPL4P4
	RPS26P8	SELENOP
	AC115223.1	EPCAM
	HSPA8P1	S100A6
	DPYD	COG6
	AC126120.1	ANKRD10
	AC016734.1	WDR33
	RARRES1	LRRC8D
	MTCO3P12	MMP14
	DPY30	RWDD4
	ALG5	SDF4
	PPIAP66	DLD
	PPIAP43	FHL2
	AC112187.1	SLC35F5
	PPIAL4C	TACSTD2
	MTND4P12	ASAH1
	TMEM183B	GUF1
	ACTBP2	ENO1
	HLA-G	LUC7L3
	RPS7P11	MTDH
	NACA3P	JKAMP
	EEF1A1P29	H2AZ2
	AC068522.1	ERAP1
	RPS26P11	TMEM192
	AC034236.1	RCN1
	MTCO2P2	WASH6P
	AC020898.1	ITGB5
	CLCA4	SLC35A2
	LYPD3	TTC19
	EEF1A1P4	OCIAD1
	AC004552.1	ADAM28
	NAMPTP1	P4HB
	PPIAP13	SFN
	RPS4XP22	SLC39A7
	EEF1A1P25	EIF4A3
	TUBAP2	TMEM30A
	CROT	GPD2
	AP000281.2	CD59
	RPL10P12	GTF2H2B
	FTH1P15	PON2
	RPL3P4	HACD3

	AC104339.1	GSS
	RPS23P8	TUFM
	PPIAP31	ATP2C1
	AC135178.7	CACHD1
	RPL10P4	CA12
	AC104563.1	MBOAT2
	RPS26P47	THYN1
	H3P16	PDIA6
	FTH1P11	LEMD3
	EEF1A1P16	RPN1
	YWHAZP3	PTTG1IP
	GAPDHP73	PRDX1
	GAPDHP63	GAA
	AL627402.1	ABCC3
	AC009245.1	IER3IP1
	RPS3AP5	EGFR
	H3P6	ANXA4
	RPL27AP5	IGFBP2
	RPS26P15	SLC27A2
	H3P47	TRAPPC3
	AC078819.1	OS9
	TCN1	SLC18B1
	HSP90AA2P	ALOX15
	FTH1P7	GNS
	RPS7P1	RRN3
	RPS27AP16	PIGX
	RPL7P19	ARL6IP1
	TMSB4XP2	RPL7AP66
	RPS26P28	AC004069.1
	RPL10AP6	LGR4
	PPIAP22	LRG1
	EEF1A1P8	SPARCL1
	ADH1B	TMEM87A
	ATP1B3	ALDH1A1
	PPIAP6	LMAN2
	RPL3P2	AC007318.1
	RPL13AP25	DDOST
	MTND6P4	CHPF
	RPS24P8	CTSB
	AC092865.1	RASA1
	RPL15P20	SLC2A1
	AL133260.1	CFH
	FTH1P16	TM9SF2
	EEF1A1P11	EML3
	RPS15AP1	CTSH
	FTH1P3	HNRNPA1P4
	APOD	ERLIN2
	AL596275.1	MTRNR2L9
	RPL15P18	LRP5
	RPL17P22	SEMA3A
	KRT6C	GFM2
	EIF4A1P10	HNRNPA1L2
	PDIA3P1	PIGG
	FTH1P20	RRM1
	EEF1A1P22	TSPAN13
	ANXA2P2	TMEM68
	AC073072.1	KRT5
	AC025518.1	ACVR1
	S100A2	ATP1A1
	RPL7P23	PRODH
	MTND6P3	RPAP2

	AL009174.1	MFSD11
	AC092670.1	FDFT1
	PPIAP29	MFSD14C
	FTH1P12	ANKRD36
	AC090543.3	SGK1
	H3C9P	NEU1
	RPL17P36	CLDN4
	MTND5P11	SCGB2A1
	MT-TE	ERLEC1
	MTCO2P12	EEF1A1P5
	MTRNR2L1	AL391121.1
	HLA-J	POR
	EEF1A1P12	RAB3IP
	EEF1A1P14	FAM3B
	AC091429.1	CALR
	FTH1P5	AC073333.1
	TPT1P9	EBPL
	EEF1A1P13	NPTN
	AC006386.2	HSD17B13
	AC012005.1	RPS21
	RPL41P2	NIFK
	MT-TA	ALS2CL
		PSEN2
		RCN2
		CHKA
		GTF2H2C
		TMA7
		ALDH7A1
		CD81
		RTN4
		DHX36
		SNRPA1
		DNAJC1
		HNRNPA1P35
		FTH1P10
		HNRNPAB
		FUCA2
		ADK
		FMO5
		RTN3
		ASCC3
		PSME1
		WASH4P
		TFB2M
		REEP5
		TMEM51
		WSB1
		SREK1
		RTN3P1
		RARS1
		HADH
		MBOAT1
		PGAP4
		FAAH2
		SMG1P4
		AC008810.1
		TLR2
		ZMPSTE24
		HNRNPA1P10
		PAPSS2
		AGL

		TMEM9
		TMEM9B
		TSPAN1
		CLN5
		TMEM59
		AL592114.1
		GUSB
		ANAPC4
		COMMD7
		MAP3K6
		ANKRD66
		DSE
		GCLC
		PRSS8
		CYP4F12
		ABCE1
		ENPP4
		ABCA5
		GLUD2
		CALM2P2
		KLHL42
		PRMT7
		ITGB6
		SESN1
		FTH1P2
		CAMK2G
		STAM
		MANF
		THOC3
		MSTO1
		ZPR1
		INPP1
		NOMO2
		LRRN1
		SRSF11
		SELENOI
		TOPORS
		PLTP
		SLC39A6
		TMEM106C
		CD47
		SORD2P
		AL158206.1
		KRT10
		EIF4A1P4
		UNC93B3
		CXADR
		CUEDC1
		IL10RB
		YWHAZP4
		ATP5F1B
		PRKAR2B
		SMC4
		PTDSS1
		COPG2
		GTF2F2
		LONRF1
		RPL10P9
		HLA-B
		RFNG
		MAN1B1

		EIF4HP1
		H3P36
		LACTB
		CNIH1
		MAPKAPK3
		JPT1
		SMAP1
		CYP3A5
		SPTSSA
		TTC29
		C1D
		PA2G4P6
		DPAGT1
		SMARCAD1
		RPL7AP6
		BX679664.3
		PLS1
		F11R
		RMDN3
		DDAH2
		BCAP29
		FSCN1
		AKAP1
		ITFG1
		ARVCF
		LOXL4
		AC090498.1
		FAM3D
		HNRNPA1P7
		HNRNPA1P12
		RHBDL2
		MPP7
		UBE2E3
		SCPEP1
		DNAJB11
		SDHA
		LAMB3
		VARS1
		NT5DC1
		PBXIP1
		TAP2
		HLA-F
		CDH1
		PRELID1
		UPK1B
		PSMD1
		TMTC4
		ITGAV
		NUDT12
		PGAM4
		RNF26
		CCDC65
		FARSB
		PARP2
		VIPR1
		ARL3
		ENY2
		SDCBPP3
		BCAP31
		B4GALT4
		RAMAC

		C5orf15
		FTH1P8
		MAD2L1BP
		RAC1P2
		NAAA
		GLULP4
		EXOSC8
		MT-TY
		BLOC1S4
		AC144530.1
		TMEM45B
		ABHD3
		HLA-L
		AC113935.1
		PRPF39
		CTNNAL1
		VPS35P1
		HSP90AB2P
		UNC93B7
		CHPT1
		TNC
		DDX50
		MLYCD
		AQP3
		LRRCC1
		TPBG
		GJB3
		PABPC1P4
		SLC25A17
		SLC44A3
		SLC26A4
		PRDX4
		GGH
		HNRNPA1P8
		LSAMP
		CDS2
		FAT1
		ATP6AP2
		MRAP2
		G6PC3
		ERO1B
		CLK1
		SNX14
		PTGES3P3
		CCT6A
		AC006511.4
		KLF9
		FKBP1C
		PIPSL
		FAM162A
		AC024293.1
		TMEM147
		ALG1
		CCDC59
		DBP
		TF
		FKBP9
		RPL14P1
		TMEM43
		ACAD10
		BORCS5

		EMC7
		ACTA1
		UNC93B6
		EDEM2
		PTK7
		ARSDP1
		TMED7
		TUSC3
		PGRMC1
		UBLCP1
		AC026271.1
		BMI1
		DYNC1I2P1
		ARMT1
		TLR1
		GPC1
		VDAC1P1
		HNRNPKP4
		RRAGA
		KIT
		ST13P3
		AC136632.1
		AL391244.2
		ETHE1
		TMED1
		RPL7P10
		HEXB
		MOSPD1
		SLC52A2
		KDSR
		SELENOS
		HLA-A
		ADH1A
		RPL22P1
		DNAJB9
		AC083873.1
		DENND10P1
		FUCA1
		HSPA8P5
		LPCAT3
		SLC35B2
		EXOSC9
		TAGLN2P1
		ATP5MC3
		EIF3FP3
		COL6A3
		CHCHD1
		TMCO1
		PABPC3
		TP63
		CTSC
		HSD17B12
		CEACAM3
		SMG1P1
		PSMC1P1
		ELMO3
		AL158801.6
		UQCRC1
		HLA-V
		MGST1
		NTS

		AL133477.1
		H3P44
		PSENEN
		RPL7AP34
		PSPC1
		LAMC1
		AL121769.1
		NDUFA12
		MT-TL1
		AC106795.1
		AC022968.1
		LRRIQ1
		LRRC17
		UFD1
		UXS1
		AC008065.1
		KRT6B
		HMGN2P17
		ALOX15P1
		RPL37AP1
		ATP6AP1
		AC099670.1
		BMP3
		FKSG70
		FAM171A1
		RPN2
		HLA-H
		EEF1A1P38
		THNSL2
		PDIA3
		AC064799.1
		KTN1
		AC209007.1
		AC104619.3
		SYT8
		COQ5
		MSH2
		NPC1
		UQCRFS1P1
		MORF4L1P1
		UBBP4
		GPR89A
		MFSD5
		SERPINB4
		HNRNPA1P48
		AP000936.3
		SIRT3
		AC092115.2
		RPS27P29
		CYP26A1
		ATP13A5
		AL109918.1
		PIGO
		ITM2B
		RPL9P32
		PFN1P1
		EIF2S2P4
		HNRNPA3P6
		HSP90AB3P
		PLLP
		CLDN1

		ANXA8L1
		RPL23P8
		AC113404.3
		HSP90AA6P
		RPL7P47
		SETP20
		PHF14
		SRD5A3
		SCAMP3
		AC244034.1
		TRIAP1
		F2RL1
		PPIAP87
		AP002784.2
		SUMO2P1
		AC005000.1
		HLA-C
		MTATP8P1
		KRT18P16
		SERBP1P5
		RPL4P5
		DCAF13
		SRSF2
		SETSIP
		FKBP9P1
		PRCP
		CD9
		IFNGR1
		RPL7P9
		TMEM212
		EIF4BP3
		PSCA
		AL049597.1
		RPL7P32
		YWHAZP5
		AC012085.1
		HNRNPCP2
		FTLP3
		APLP2
		SETP14
		UBE2I
		CDC42P6
		ANXA8
		AL354702.1
		FGFR3
		H3-5
		TSPAN6
		EIF4A1P2
		RPL7P1
		GAPDHP65
		AC105250.1
		EPHA1
		AC002075.2
		RPS7P10
		EEF1A1P19
		EIF5AL1
		RPL10AP2
		ARSD
		XRCC6P2
		RPL13AP20
		AC092597.1

		EIF4BP6
		AC099560.2
		RPL12P38
		RPS26P6
		LDHBP2
		KRT18P11
		AC004057.1
		RPL34P26
		BZW1P2
		AC092683.1
		S100A4
		HLA-DRB6
		C1GALT1C1
		RPS26P31
		SLC5A8
		MTCO1P40
		GAPDHP44
		RPL7AP11
		PPIC
		TMX1
		ITGA6
		GAPDHP61
		PSMC1P5
		EIF4BP7
		PPIAP16
		EEF1A1P7
		RPSAP19
		AC005480.2
		UNC50
		MIR22HG
		RPS26P8
		AC115223.1
		HSPA8P1
		DPYD
		AC126120.1
		AC016734.1
		RARRES1
		MTCO3P12
		DPY30
		ALG5
		PPIAP66
		PPIAP43
		AC112187.1
		PPIAL4C
		MTND4P12
		TMEM183B
		ACTBP2
		HLA-G
		RPS7P11
		NACA3P
		EEF1A1P29
		AC068522.1
		RPS26P11
		AC034236.1
		MTCO2P2
		AC020898.1
		CLCA4
		LYPD3
		EEF1A1P4
		AC004552.1
		NAMPTP1

		PPIAP13
		RPS4XP22
		EEF1A1P25
		TUBAP2
		CROT
		AP000281.2
		RPL10P12
		FTH1P15
		RPL3P4
		AC104339.1
		RPS23P8
		PPIAP31
		AC135178.7
		RPL10P4
		AC104563.1
		RPS26P47
		H3P16
		FTH1P11
		EEF1A1P16
		YWHAZP3
		GAPDHP73
		GAPDHP63
		AL627402.1
		AC009245.1
		RPS3AP5
		H3P6
		RPL27AP5
		RPS26P15
		H3P47
		AC078819.1
		TCN1
		HSP90AA2P
		FTH1P7
		RPS7P1
		RPS27AP16
		RPL7P19
		TMSB4XP2
		RPS26P28
		RPL10AP6
		PPIAP22
		EEF1A1P8
		ADH1B
		ATP1B3
		PPIAP6
		RPL3P2
		RPL13AP25
		MTND6P4
		RPS24P8
		AC092865.1
		RPL15P20
		AL133260.1
		FTH1P16
		EEF1A1P11
		RPS15AP1
		FTH1P3
		APOD
		AL596275.1
		RPL15P18
		RPL17P22
		KRT6C
		EIF4A1P10

		PDIA3P1
		FTH1P20
		EEF1A1P22
		ANXA2P2
		AC073072.1
		AC025518.1
		S100A2
		RPL7P23
		MTND6P3
		AL009174.1
		AC092670.1
		PPIAP29
		FTH1P12
		AC090543.3
		H3C9P
		RPL17P36
		MTND5P11
		MT-TE
		MTCO2P12
		MTRNR2L1
		HLA-J
		EEF1A1P12
		EEF1A1P14
		AC091429.1
		FTH1P5
		TPT1P9
		EEF1A1P13
		AC006386.2
		AC012005.1
		RPL41P2
		MT-TA
