# Supplementary material for: Transcriptome of nasopharyngeal samples from COVID-19 patients and a comparative analysis with other SARS-CoV-2 infection models reveal disparate host responses against SARS-CoV-2
Source: J Transl Med. 2021 Jan 7;19:32. doi: 10.1186/s12967-020-02695-0 (PMC7790360; doi:10.1186/s12967-020-02695-0)
Supplement: Supplementary file 6 — Additional file 6. Differentially expressed genes in different SARS-CoV-2 infected cell types. [file 12967_2020_2695_MOESM6_ESM.pdf]

Additional file 6 Differentially expressed genes in different SARS-CoV-2 infected cell types.

BD_Nasal	GSE147507_Lung	GSE150316_Lung	GSE147507_NHBE	GSE148729_CALU3
RNA5S14	AAK1	IGHV4-34	AC006058.4	ZC3HAV1
RNA5S11	AAMP	IGHV4-59	AC092964.1	DDX58
RNA5S8	AAR2	MIR205HG	AC099336.2	OAS3
RNA5S1	AARS1	IGKV2-24	AC144530.1	OAS2
RNA5S10	AASS	IGLV3-27	AL445490.1	GBP1
RNA5S13	AATF	IGHV3-73	ALDH1A3	IFIT3
RNA5S15	ABCA1	IGHM	ANXA3	IFIT2
RNA5S2	ABCB10	IGLV1-47	AOAH	ZNFX1
RNA5S16	ABCB11	FAM26F	AP001324.1	HELZ2
RNA5S3	ABCB7	IGLV3-21	BCL2A1	TRIM22
RNA5S6	ABCC10	IGLV9-49	BCL2L11	RSAD2
RNA5S17	ABCC2	IGHV5-51	C1QTNF1	CMPK2
RNA5S7	ABCC3	APLNR	C1R	HERC5
RNA5S12	ABCD4	IGLV3-25	C3	MX1
RNA5S5	ABCF1	IGKV1-12	CAMK2D	GBP4
RNA5S4	ABCF2	CD3D	CCDC14	IFI27
RNU1-28P	ABCF3	IGLL5	CCL20	TAP1
RNVU1-18	ABCG2	LAG3	CCNA2	CXCL10
RNU1-4	ABHD12B	IGHV1-18	CD3E	ISG20
RNVU1-29	ABHD13	IGHV3-74	CENPA	PARP14
RNU1-2	ABHD14B	IGKV1-39	CEP55	MX2
RNU1-1	ABHD17A	DERL3	CES1	IFIT1
RNU1-27P	ABHD2	KRT4	CES1P1	APOL6
RNU1-3	ABHD3	IGHV3-48	CFB	WARS1
RNVU1-7	ABHD5	HIST1H3G	CLEC5A	IDO1
RNY1	ABHD6	IGHV3-49	COL8A1	LAMP3
RNU4-2	ABI1	FOLR2	CPA4	IFI35
RNA5S9	ABL1	IGHG4	CSF3	ISG15
FP671120.2	ABL2	IGHGP	CSTB	TRANK1
CR392039.1	ABLIM1	HIST1H2BL	CTSH	OAS1
VTRNA1-1	ABLIM3	IGHV3-53	CXCL16	TNFSF10
RNA5SP370	ABR	CD27	CXCL5	IFITM1
FP236383.6	ABRACL	HIST1H3C	CXCL9	PMAIP1
RNA5SP202	ABT1	IGHV3-15	DUSP4	IRF1
RNVU1-28	ABTB1	HIST1H2BF	EDN1	IRF7
CDR1	AC000089.1	HIST1H2AH	EEF1A1P13	SAMD9
SNORA73B	AC002075.2	LTB	EFNA1	SAMHD1
RNU1-11P	AC004057.1	HIST1H2BO	EIF4BP3	JUN
RNY4	AC004151.1	KIF2C	EIF4BP6	TXNIP
FP236383.1	AC004158.1	HIST1H3F	FAT2	PARP10
RNA5SP389	AC004453.1	HIST1H2BH	FBXW7	GSDMB
RNU4-1	AC004552.1	RAMP3	FCGBP	IFIT5
FP236383.9	AC004846.1	APOL4	GAS6-AS1	HCP5
H4C5	AC004846.2	HIST1H2BI	GCLC	PPP1R15A
MIR3648-1	AC004847.1	HIST1H2AL	HELZ2	DHX58
SMR3B	AC004877.2	PSMB10	HK3	CCL5
H4C11	AC005005.4	SNORA13	HSH2D	UBE2L6
RNA5-8SN2	AC005083.1	CCNB1	HSPB1P1	RNF213
RNA5-8SN3	AC005229.4	EXOC3L1	ICAM1	HLA-B
FP671120.4	AC005261.2	RNU1-28P	IFI27	PLEKHA4
RNA5-8SN1	AC005332.6	ITM2C	IFI44L	HLA-F
FP236383.3	AC005336.1	LTF	IFI6	STAT2
MIR3648-2	AC005912.1	C12orf75	IFITM1	CX3CL1
FP671120.1	AC006001.4	HIST1H1B	IFITM10	XAF1
FP236383.10	AC006252.1	HIST1H3I	IKBKE	TAP2
RN7SL5P	AC006262.1	HIST1H3B	IKZF1	DDX60

RNA28S5	AC006386.2	MYBL2	IL10	USP18
PRB4	AC006435.2	CLEC3B	IL6	LGALS9
PRB3	AC006511.4	PLVAP	IRF7	IFI16
RNA5SP298	AC006548.28	KIFC1	ITGA5	NFKBIA
FP236383.5	AC007336.1	HIST1H2AG	IVL	HLA-E
FP236383.4	AC007384.1	HIST1H2BN	KB-1507C5.4	PTGER4
FP236383.12	AC007686.3	VAMP5	KRT6B	SAMD9L
FP671120.7	AC007743.1	MMP28	LINC00504	DDX60L
RN7SL4P	AC007881.1	DLGAP5	LTB	IFI44
RNA5SP226	AC007906.2	ICAM2	MAFF	AL669918.1
RN7SL752P	AC008147.2	RARRES3	MAP3K8	HERC6
RNY3	AC008537.3	HIST2H2AB	MAP7D2	KLF4
AC079601.1	AC008555.2	IFI27L2	MCM4	TIPARP
RNA5SP145	AC008676.1	HIST1H3D	MIR155HG	DTX3L
RNA5SP149	AC008687.2	LIMD2	MIR3142HG	CXCL8
RNA5SP429	AC008760.2	BUB1	MME	RTP4
AL135938.1	AC008764.2	STYXL1	MMP9	TENT5A
RNA5SP74	AC008969.1	HIST1H2AE	MX1	NFKBIZ
CRNN	AC009005.1	CKS2	MX2	MAP3K8
RNY3P1	AC009054.2	BANK1	MYLK	PARP12
FAM27E2	AC009126.1	MRPL2	NANOS1	OASL
FP236383.7	AC009245.1	NCF1	NCCRP1	STAT1
RN7SL396P	AC009275.1	RAMP2	NEDD9	PLAAT4
FP671120.5	AC009283.1	SP140	NHBE_DE	IFNL1
H1-4	AC009303.4	HIST1H2BG	NID1	NLRC5
AL162581.1	AC009318.1	NDUFA3	NT5DC3	SP110
CRCT1	AC009961.1	CIITA	OAS1	CEACAM1
RNA5SP335	AC010173.1	THYN1	OR7E47P	CCL20
RNA5-8SP6	AC010226.1	ITGB4	OSBP2	BATF2
AC024051.11	AC010326.4	GIMAP7	P2RY6	PARP9
AC024051.5	AC010970.1	HDAC9	PARP12	UBA7
AC024051.12	AC011472.2	CORO1A	PCDH17	PSMB8
AC024051.1	AC011498.7	CLEC14A	PDCD4	TEP1
FP236383.8	AC011933.1	CRIP1	PDGFB	ETS2
RNU2-1	AC012085.1	PSD4	PDZK1IP1	CFB
AC024051.2	AC012368.1	FCGRT	PGLYRP4	VIM
SNORD17	AC015912.3	TXNDC5	PLA1A	NMI
AC024051.7	AC015922.3	HN1	PLAT	TNFAIP2
AC024051.10	AC016142.1	SNRPD2	PLSCR1	GBP3
AC024051.3	AC016745.2	HIST1H2BC	POU2F2	HLA-C
AC024051.8	AC016747.1	CXorf36	PPARGC1A	PMEPA1
AC024051.6	AC016957.2	PLCG2	PRELID1	APOL1
RNA5SP161	AC018638.1	HIST1H2BD	PRELID2	NCOA7
AC024051.4	AC018638.2	LMAN2	PRF1	TRIM5
AL161626.1	AC018868.1	PSME1	RAB15	IFIH1
LINC01783	AC019322.6	NCAPD2	RAB7B	PML
CXCL11	AC020612.3	CLU	RFTN1	HLA-A
H4C8	AC020765.2	RFX5	RND1	IFITM2
FABP6-AS1	AC020916.1	ATPIF1	RPL10AP6	AHNAK
RN7SKP71	AC021106.1	PRDX5	RPL21P16	SNORD3A
RN7SKP203	AC022149.1	PPP1CA	RPL37P2	JAK2
UBQLNL	AC022211.4	PSMD4	RPL7AP50	LAP3
CTA-384D8.31	AC022274.1	SPAG9	RPL9	DUSP1
AC092299.7	AC022400.9	SERTAD2	RPS2P55	XRN1
BPIFB2	AC022613.1	ROCK2	S100P	SLC15A3
SIGLEC16	AC022916.2	CREB3L2	SAA1	BBC3
TRPV1	AC022929.2	FAM168B	SAA2	B2M
H2BC8	AC022966.1	NABP1	SEC14L4	LY6E
FAM27E3	AC023157.1	PIK3R1	SERPINB1	KDM6A
RNA5SP481	AC023157.3	AFF4	SMTN	BIRC3

H4C2	AC023590.1	DDX21	SOD2	FNDC3A
LINC02176	AC025518.1	SNX13	SPRR2B	NEAT1
MIR663AHG	AC025580.2	PDZD8	SPRR2D	SCD
SCARNA7	AC025884.1	BCL2L2	SPRR2E	HLA-H
C1QB	AC027801.1	SLIT3	ST3GAL5	IL1A
GDF5	AC034236.1	CHSY1	STAT5A	OPTN
SIGLEC1	AC040160.1	LRRTM2	STRBP	RN7SL3
CA6	AC040162.1	FOXO3	SUSD6	PPM1K
SCARNA6	AC040970.1	YBX3	TCF4	NUB1
MZB1	AC055733.1	LPP	TCIM	CD74
CD8A	AC060766.4	CLIC4	TFCP2L1	NFKB2
HTN3	AC060766.7	FNIP1	TGM1	MLEC
AC087276.3	AC060814.2	TNFAIP3	TNF	TRIM21
MOCS1	AC064799.1	ELK4	TNFAIP2	CXCL11
AC084082.1	AC067930.4	NUPL1	TNFAIP3	KLF6
RN7SKP80	AC067945.3	SPRY4	TNFSF14	SAT1
CARNS1	AC068039.4	TTC19	TREML4	ABHD2
MT-RNR2	AC068448.2	RLIM	TRIM47	ETV7
SIGLEC11	AC068888.1	AGFG1	VGLL4	CFTR
AP001462.1	AC068987.2	PHLDA1	VNN1	EGR1
THSD4-AS1	AC069547.1	SLC6A6	VNN3	MXD1
AL354919.1	AC073072.1	FOXO1	XAF1	AFAP1
STATH	AC073343.1	IMPAD1	XDH	SLC1A3
AL137779.1	AC073349.2	OSBPL8	YTHDC2	SNORA73B
BSN	AC073548.1	MAPK6	ZC3H12A	APOL2
CYP2D8P	AC073861.1	ZFAND5	ZCCHC10	EPSTI1
H2AC8	AC073869.1	PPP1R3B	ZNF488	ATF3
SKAP2	AC078819.1	CTB-89H12.4		IL6
SNX29P1	AC079228.1	PAG1		ZFP36
G2E3-AS1	AC079250.1	KLF6		TRIM38
AC016168.4	AC079922.1	NEDD9		HLA-DRA
IKZF3	AC079949.1	TXNRD1		CYLD
PPP1R1A	AC080023.1	ENPP4		RIPOR2
H2BC4	AC083843.3	RP11-463O12.5		BTN3A1
CHAC1	AC083899.1	KITLG		OGFR
AL596325.2	AC084871.1	FLJ42393		ERAP2
MAP1LC3C	AC084880.1	UPP1		PLAUR
HOPX	AC087473.1	CSGALNACT2		AL645922.1
IFIT2	AC087623.2	SLC16A7		TMEM140
RN7SKP255	AC090114.2	NRCAM		SNORA73A
CXCL10	AC090543.3	GK		DNAJB4
KRT78	AC090559.1	EMP1		NFE2L3
AC068987.2	AC090587.2	BHLHE40		NT5C3A
RN7SL391P	AC091230.1	RP11-107E5.3		IKZF3
ARHGAP40	AC091304.1	AKAP12		ARRDC3
ADRA2A	AC092118.1	NUDT16		TDRD7
CNFN	AC092597.1	GBE1		TRIM69
TERB1	AC092670.1	ADAMTS9		SERPINB2
TRIM34	AC092683.1	TMED7		TPM1
SLC9C2	AC092718.4	VGLL3		RASGRP3
CACNG8	AC092865.1	SLC6A8		TAPBPL
CLVS1	AC092919.3	PTP4A1		DICER1
H1-2	AC092964.1	ATP13A3		TOP2A
SPRR2D	AC093010.3	NAMPT		PRKD2
SLC23A3	AC093323.1	ADAMTS1		SHFL
AC005921.4	AC093690.1	SLC2A3		BST2
LMX1B	AC093752.1	RHOQ		PNPT1
PGBD5	AC097263.1	PDLIM3		PIK3AP1
C8orf34	AC097523.1	ERRFI1		PTGS2
SSPO	AC098583.1	BEST1		TYMP

FLG2	AC098851.1	IER3		TRIM26
AP000708.1	AC099336.2	SLC7A2		NEURL3
AC006435.4	AC099343.4	ADAMTS15		HSH2D
H2BC5	AC099524.1	ZNF638-IT1		PTX3
FGF17	AC099560.2	IGSF10		ADAR
ABCA8	AC099789.1	KLF9		MED12L
LINC01562	AC104339.1	NME9		HIP1R
FXYD6	AC104563.1	CD300E		CYP1A1
ARHGAP15	AC104791.2	KIAA1614		CCNL1
THRB-IT1	AC104981.1	NCR3LG1		MB21D2
LINC01460	AC105250.1	IL6		SEMA3A
AC073571.1	AC105942.1	RP11-415J8.3		ALCAM
MUC5B	AC106795.1	ADAMTS9-AS1		MYD88
RN7SL274P	AC107375.1	RP11-752L20.3		SOD2
COL9A3	AC108058.1	SPDYA		CFAP54
CROCC2	AC108134.1	GADD45A		SP100
AC005696.4	AC108925.1	HIF1A-AS2		KIF20A
ABI3	AC109322.1	USP53		SYNE1
BACH2	AC109829.2	EGLN3		SCN3A
MAL	AC110285.6	FAM110C		INHBA
AC091132.5	AC110749.1	C11orf96		NR3C1
ZBP1	AC112777.1	NAV2-AS5		OTUD1
AL445665.1	AC113398.1	CTD-2033D15.2		SLC12A7
SSUH2	AC113935.1	GLDN		FOS
RGL4	AC114498.1	SLED1		IL32
PPP1R1B	AC114728.1	NAMPTP1		TFRC
AP001207.3	AC115223.1	APOLD1		LINC00944
NR1I3	AC116049.1	HILPDA		SCARNA5
POU2F2	AC116407.2	FOSB		FZD4
LINC01134	AC116533.1	CYR61		FST
AC138866.2	AC116914.2	EGR1		IRAK2
AC079949.1	AC121761.1	RP11-417F21.1		B4GALT5
AC138866.1	AC124067.4	NR4A3		GCA
IFIT1	AC125437.1	SLC19A2		SQLE
LINC02832	AC125807.1	PTX3		MT2A
CRIP1	AC125807.2	PTGS2		NUAK2
RBM34	AC127502.1	LIF		TRAFD1
MIR99AHG	AC127502.2	BRD7P4		NFKBIE
IRF8	AC129507.1	CTD-2026K11.6		CFH
IL15RA	AC131011.2	RP11-212E4.1		KYNU
DYSF	AC131235.2	FGG		CNP
AC114498.1	AC132192.2	RP1-309I22.2		GRB10
LINGO4	AC132812.1	AC007278.3		CD274
AC011466.4	AC133134.1	RP11-212I21.3		PSMB9
AC015967.2	AC136475.5	RP1-29C18.10		CSF1
STAC2	AC136475.9	RP11-314N13.9		RPS6KC1
TGM3	AC138305.3	AC007278.2		TRIM31
BCL2L14	AC144530.1	RP11-635L1.2		BTN3A3
C10orf82	AC145285.2	SLCO4A1-AS1		APOL3
AP001020.2	AC145350.2	RP11-393I2.2		ANXA4
CDYL2	AC146944.3	RP11-291I6.2		TGM2
AC099489.1	AC159540.2	CTD-2184D3.5		MOV10
FRMD6-AS1	AC211476.10	SDCBPP1		SNHG3
OASL	AC211476.5	EDN2		CTSS
LETM2	AC234781.1	CCL20		HDAC9
MX2	AC240565.3	SLC5A8		LGMN
AL691477.1	AC240565.4			BRD2
AL162258.2	AC241952.2			PRKDC
PLEKHD1	AC243964.4			REL
VASH2	AC244669.1			KMT2C

HERC5	AC245297.1			TNIP1
AC004815.1	AC245297.4			RMRP
RNF139-AS1	AC246787.1			RMRP
ZFHX2	ACAA1			TNFRSF10B
CMPK2	ACACB			CYP24A1
RUSC2	ACAD8			ZBP1
CPED1	ACADVL			SNORD17
ZBTB8A	ACAP3			CHD2
ODF3B	ACAT2			SPOCK2
IFITM1	ACBD5			ACACA
ECM1	ACBD6			PGK1
CD53	ACBD7			FAP
IFIT3	ACD			LGALS3BP
AC016590.1	ACHE			PLSCR1
FCER1G	ACKR3			DHCR24
HTN1	ACO2			AL049839.2
GUSBP3	ACOT1			AF117829.1
ZNF324B	ACOT13			PELI1
AEN	ACOT2			AMBRA1
NHLRC4	ACOT8			PGAP3
CHDC2	ACOT9			ADRB2
FABP6	ACP2			LINC02068
H2AC20	ACP5			SRPK2
ZNF250	ACRBP			CXCL2
PTGER2	ACSF2			AREG
IFITM3	ACSF3			CLTRN
PLEKHM3	ACSL1			CBX6
SMAD9	ACSS1			TNFAIP3
SPRR2A	ACTB			CEBPB
TBX6	ACTG1P17			SERPINA3
ZC3H3	ACTL6A			NEDD4
CD37	ACTN1			F2RL1
IFI44L	ACTN4			SEMA7A
CROCC	ACTR1A			HMGCR
AL031282.2	ACTR1B			UBN2
PIK3AP1	ACTR3			ARL14
SLC2A5	ACTR3B			TLR3
ISG15	ACTR5			PIK3R3
ZNF579	ACVR1			KRT17
RSAD2	ACVR1B			LIF
SYT12	ACVR1C			CFLAR
AC127164.1	ACVRL1			SPAG9
AKNA	ADA			RCAN1
AC025580.3	ADA2			PSME2
WDR49	ADAM11			MIR155HG
XRRA1	ADAM15			TM4SF4
AC027243.1	ADAM17			CREB5
GUSBP2	ADAM19			ABL2
ING1	ADAM1A			ATP9A
H4C14	ADAM8			ST3GAL2
AC020741.1	ADAMTS1			FOSB
RAMP2-AS1	ADAMTS2			APOBEC3G
C1orf229	ADAMTS3			PLXNA2
H4C15	ADAMTS9			CD59
NUPR1	ADAMTS9-AS1			CD24
AC009646.2	ADAMTSL3			SP140L
BCDIN3D	ADAMTSL4-AS1			RNVU1-28
ISG20	ADAMTSL5			GADD45B
MT2A	ADAR			RIPK2
CFAP46	ADARB1			ETV3

MUC5AC	ADAT2			IFITM3
AC111149.2	ADCK2			SNORA63
CHP2	ADCY3			LASP1
SPTB	ADCY4			HDLBP
CCL5	ADCY5			ACO1
DLEC1	ADCY6			CMTM4
RNF222	ADCY9			SORT1
LBHD1	ADD1			TSPAN8
NLRC3	ADD3			MAP4K4
IL1RN	ADGRD1			UNC93B1
P2RX7	ADGRD2			IRF1-AS1
EPHA10	ADGRE1			TMBIM6
PNRC1	ADGRE2			CASC19
PLPPR2	ADGRE5			VAT1
FER1L5	ADGRF2			MYH9
MAPK12	ADGRF5P2			RNF19B
RP11-706O15.5	ADGRG1			BRI3BP
AL592211.1	ADGRG3			TRAF1
PML	ADGRL2			THEMIS2
PLAAT2	ADH5			BAZ2B
AC138932.1	ADH7			LDHA
CA5A	ADIPOR1			HSPA5
USP18	ADIPOR2			LGALS4
SPI1	ADIRF-AS1			RELB
HNRNPA1P40	ADK			SLC12A2
SPRR2E	ADM5			SEPTIN9
MCF2L	ADNP			XBP1
FGR	ADNP2			PLS1
PPP1R16B	ADPRM			TNFSF15
CCDC33	ADRA2A			KIAA0100
ENKD1	ADRM1			NCAPD2
MARCKSL1	ADSL			ATP1A1
AC026523.2	ADSS1			CCNB2
CFAP74	ADSS2			SDC1
HMOX1	AEBP1			SMURF1
GPR65	AEBP2			ENDOD1
NINL	AEN			ZEB2
AP001107.1	AF165147.1			SLC3A2
MICB	AFAP1			ITGB1
OAS2	AFAP1L1			BACH2
UBE2T	AFDN			ZBTB20
MAP4K2	AFF4			MCL1
RPL37	AFG3L1P			DCP1A
IRF7	AFG3L2			FRS2
NTAN1	AFTPH			NUDT3
IFI27	AGAP1			HIVEP2
DTX2P1	AGAP10P			NUCKS1
AC027290.3	AGAP3			EPHA4
ABHD8	AGAP4			SDC4
UBE2L6	AGAP6			N4BP1
AC019117.3	AGAP7P			C3
TEKT2	AGBL5			MYCBP2
AC118344.4	AGER			IL15RA
NATD1	AGFG1			NEDD9
BEX2	AGFG2			ACKR4
GNL3L	AGK			GPBP1
SRCIN1	AGMO			MSMO1
SAMD9	AGO1			UHRF1BP1
LINC01551	AGPAT2			DHCR7
SGTB	AGPAT4			NFAT5

TNFRSF14-AS1	AGRN			DAB2IP
SERPING1	AGTR1			CMTR1
BICD2	AGTRAP			YBX1
SRGAP3-AS2	AHCTF1			EEF1A1
DUSP5	AHDC1			SPP1
PRR29	AHNAK			ARL6IP1
AC008079.1	AHR			MLKL
CD96	AHRR			TRIM25
RP11-706O15.3	AHSA2P			ANXA2P2
RN7SL718P	AIDA			SEC16B
AHNAK2	AIF1L			SMCHD1
NIN	AIFM2			RN7SL4P
OCEL1	AIM2			BTN2A2
CYP2F1	AIMP1			SNORA74A
CCND3	AIMP2			ARHGEF28
RPS15	AIP			NAPA
MUC13	AJ003147.3			H2BU1
AKAP12	AJM1			RSRC2
GUSBP1	AJUBA			TRIM56
SPINK5	AK3			AC107959.3
ZNF76	AK7			TOP2B
PTCHD4	AKAP1			FDPS
CRYBG3	AKAP10			ASPM
ZFP36	AKAP11			IER5
CARD16	AKAP12			EGOT
DHX35	AKAP13			CPEB3
SAMD9L	AKAP17A			KPNA2
ST3GAL2	AKAP5			SCARNA6
PPDPF	AKAP8L			DUSP16
EYA1	AKIRIN1			PER1
SP2	AKIRIN2			GDF15
CCDC40	AKNA			GBP5
IFIH1	AKR1A1			RARRES1
AMOTL2	AKR1B1			LBR
NUMA1	AKR1B10			ZNF462
FAM222A	AKR1B15			RN7SL2
CCDC88C	AKR1C2			ZBED5
RND1	AKR1C3			TAPBP
UBA52	AKR1C7P			IDI1
ARHGAP39	AKR7A2			NCAPG
WDR62	AKT1			SECTM1
AC134407.2	AKT2			PATL1
SCGB1A1	AKT3			DAPP1
ATF3	AL008729.1			CHST9
ZNF500	AL009174.1			ANXA5
ALPK3	AL022322.2			HMMR
RP11-589F5.3	AL031058.1			ARHGAP26
TICAM1	AL031283.1			CCNB1
UBAP2	AL031595.3			FDFT1
CEP135	AL031777.1			ERAP1
ZNF329	AL033397.1			EHD4
CCDC78	AL033519.3			AC109326.1
C12orf50	AL034379.1			IRF9
EVI2B	AL034397.3			TAAR3P
YY1AP1	AL035071.1			RND1
TRIM14	AL035661.1			AHNAK2
NKX3-1	AL049555.1			AC015688.4
CCDC159	AL049840.6			RICTOR
JPX	AL049873.1			BTN3A2
ZNF335	AL109806.1			GTPBP1

MRTFA	AL118516.1			UBE2Z
FOXG1	AL121603.2			DUSP5
CCDC106	AL121895.1			ZNF274
SYTL5	AL121944.2			ASAP2
COTL1	AL121949.1			AP2M1
OAS1	AL133260.1			CASP1
H2AC6	AL133335.1			NAV2
AC004151.1	AL135745.1			UBD
MUC21	AL135818.1			H2AZ2
HOOK1	AL158050.1			BICC1
H2BC18	AL158801.6			NOCT
OAS3	AL159141.1			BLZF1
PREX1	AL161431.1			DPYSL2
CCDC88B	AL161626.1			MDH1
FTL	AL161787.1			CRYZL2P-SEC16B
FOSB	AL162151.2			SPRY2
WNK2	AL162231.2			HERPUD1
MAST3	AL353804.1			HLA-J
EPSTI1	AL355309.1			DUOX1
PCNT	AL355355.2			FSCN1
NFATC3	AL356273.6			RPLP0
HIGD2A	AL356489.2			NAMPT
KIAA0040	AL391427.1			XRCC5
C15orf62	AL445363.3			IDH1
CEP112	AL445490.1			PPP1R10
ARHGAP26	AL445524.1			PHACTR4
SGSM1	AL445931.1			CSRNP1
MVB12B	AL512306.2			SERPINB9
SPAG9	AL512408.1			ANXA2
SYNPO	AL513314.2			PERP
PKD1P5	AL589743.1			PSME1
SHANK2	AL590705.3			NEDD1
CLEC16A	AL590867.2			CLUHP3
ELF4	AL591846.2			H1-0
NCCRP1	AL592211.1			SH3KBP1
H2AC18	AL596202.1			CTSD
HELB	AL627309.5			RASGEF1B
FCGR2A	AL627309.6			KDM2A
ATP5F1E	AL669831.1			CYP2J2
SIPA1L3	AL691432.2			CRYZ
H2AC19	AL691447.2			SOCS1
SEC24A	AL732372.2			UPK1B
POLR2M	AL772337.3			GLCCI1
GNG5	AL807752.1			PLEKHA7
KAT2B	AL807757.2			GNG12
A2ML1	AL845472.1			TSC22D2
TNFAIP3	ALAS1			HLTF
HSF1	ALDH1A1			TMEM62
MFN1	ALDH1A3			JUNB
AC126755.1	ALDH3A1			H2AZ1
ALMS1	ALDH3B1			SOCS3
FBXO48	ALDH3B2			AHR
RABIF	ALDOA			CAT
SLC25A23	ALDOC			STK10
MCUB	ALG1L13P			TRAM1
SOBP	ALG3			KCNV1
HELZ2	ALG5			AL356488.2
CBX6	ALG6			SCARNA2
SRCAP	ALKBH2			NR2F1-AS1
TACC2	ALKBH5			DSEL

FAM193A	ALKBH6			ACAT2
SERPINB8	ALKBH7			TET2
PRPF3	ALOX5AP			CFAP57
SYTL2	ALPK1			ADM
MBNL1	ALPL			BACH1
PATL1	ALS2CL			FUT4
ZMIZ2	ALYREF			IARS2
ZNF358	AMBRA1			GMPR
BLZF1	AMDHD1			TGFBI
STK10	AMER1			GBP1P1
NIBAN1	AMFR			SNORA70
CDK18	AMIGO2			DND1
PHC3	AMMECR1L			PDZD2
FYCO1	AMN1			SNORA12
LGALS9B	AMOTL1			GALNT10
TEX9	AMPD2			CD47
MX1	AMT			ZNF503
FNDC3B	AMTN			CD46
CLPB	ANAPC1			AC019117.4
PRRC2A	ANAPC11			IL4I1
APOL3	ANAPC13			COL11A2
CENPBD1P1	ANAPC2			IFNB1
RAB8A	ANAPC5			TNFSF13B
DAP	ANAPC7			RND3
ZNF592	ANGEL1			SCARNA13
GTDC1	ANGPTL2			TRIM14
MOB3A	ANGPTL4			RPS25
TADA2B	ANK3			ZNF200
SH3KBP1	ANKDD1A			CEP350
SAMD4B	ANKDD1B			HMGB3
TRAF3IP2	ANKFY1			VPS41
SPDEF	ANKH			ZCCHC2
CDKN2B	ANKLE2			CTSH
RBMS2	ANKMY1			RNU1-67P
CIZ1	ANKMY2			SKIV2L
DUSP3	ANKRA2			EEF1A1P5
RFX5	ANKRD10			HMGB1
MAP3K11	ANKRD11			ZC3H12A
TENT5C	ANKRD17			PPP1R15B
TBC1D15	ANKRD22			APLP2
RPL36AL	ANKRD27			ARAP2
R3HDM2	ANKRD29			NEB
MLPH	ANKRD36			WTAP
S100A11	ANKRD36B			MAFF
RND3	ANKRD37			HACD3
UBB	ANKRD42			RPS3
PAK4	ANKRD46			CSKMT
RASSF9	ANKRD49			SNORD3B-2
ATXN7	ANKRD50			HSD17B7P2
RUNDC1	ANKRD54			CENPF
IQCE	ANKS1A			SEPTIN2
R3HDM1	ANKZF1			SLFN5
NEK6	ANO6			EPC1
JADE2	ANP32A			ANKS1A
TCOF1	ANPEP			S100A6
CSNK1G2	ANXA1			CAPN2
TOB2	ANXA2			DBI
ATN1	ANXA2R			BIRC5
NCOR2	ANXA3			IFNL2
IL1R1	ANXA5			ENO1

SPECC1	ANXA6			BAZ1A
ANKRD17	ANXA8			ARID4B
BICDL1	AOC3			CPD
RAB3B	AOPEP			PSMB8-AS1
VPS37B	AP000446.1			PSMB10
CRY2	AP000487.1			HCG27
ACSS1	AP000640.2			TAGLN2
WARS1	AP000769.1			CANX
OPTN	AP000844.2			PATL2
ZNF609	AP000892.4			DNAH17
PDE4DIP	AP000936.3			IQGAP3
FCHSD2	AP000974.1			KIF4A
SP3	AP001324.1			SLC5A4-AS1
WWC1	AP001830.1			RBMS2
MLXIP	AP001972.5			ACTB
ZFP36L2	AP002784.2			PRKACB
SORBS3	AP003068.2			MED13
SND1	AP006621.2			ARL6IP5
FOXK1	AP006623.1			INTS6
GTPBP1	AP1B1			GABBR1
CCDC69	AP1M1			GOLIM4
C6orf132	AP1M2			RBCK1
LINC00963	AP1S2			NECTIN2
ADGRG1	AP2A1			FOSL1
SRSF5	AP2A2			RNF149
ACTN4	AP2M1			PPP4R4
EWSR1	AP3B1			GLO1
HK1	AP3D1			STING1
TMED2	AP3M1			FAS
CCZ1B	AP3M2			H2AW
PSMD2	AP3S1			FADS1
VAMP3	AP3S2			RBM39
CTNND1	AP4M1			CITED2
ARRDC3	AP5Z1			BNIP3L
RDH10	APBA3			CIITA
EIF3CL	APBB1			DIP2B
PRKAR1A	APBB1IP			MFSD14A
PPP2CB	APBB3			SUN2
TSPYL1	APC			CAPS
SRP9	APEH			NT5E
SMARCA2	APEX2			VTRNA1-3
GLUL	APH1A			STMN1
RBM25	API5			AL136295.5
TNFRSF21	APLF			TSN
SELENBP1	APLP1			SERPINB10
DHCR24	APLP2			MAOB
MAT2A	APOBEC3A			TPX2
EIF3C	APOBEC3C			AL021578.1
SCARB2	APOBEC3G			KIAA0040
TMPRSS4	APOBR			GABRP
EIF4G1	APOC1			IFNL3
ENC1	APOD			FOXO1
HNRNPH1	APOL1			ARL5B
WDR1	APOL3			IFI6
ADSS2	APOL6			PTTG1IP
TMEM123	APOLD1			VAV3
IL33	APOO			CXCL1
CCZ1	APP			CD2AP
FBXL5	APPL1			CDKN1A
ZBTB7A	APRT			HLA-DQB1

HNRNPK	APTR			CRIP2
PLEKHB2	APTX			REV3L
HNRNPU	AQP1			KCNJ15
ARL8B	AQP3			LRP2
DHX15	AQP9			CLK1
UBQLN1	AQR			HLA-DOB
ANXA7	ARAP1			PLCG2
NDRG2	ARAP3			CXXC4
TOMM20	ARC			GTPBP2
GANAB	AREG			EEF1G
CTBP2	ARF3			ARHGEF38
CBWD6	ARF5			MYH10
ITGB1	ARFGAP1			NCEH1
ASPH	ARFGAP2			PNP
ATP8B1	ARFGAP3			UBC
PFN2	ARFGEF1			AC006064.5
DDB1	ARFGEF3			ITGB6
ARGLU1	ARFIP1			SCARNA10
SF3B1	ARFIP2			SETD5
ANPEP	ARFRP1			PTTG1
DDX1	ARGLU1			SIPA1L2
NCSTN	ARHGAP1			CYP51A1
DNAJC10	ARHGAP10			KDM6B
DDX5	ARHGAP15			DDIT3
IFT57	ARHGAP17			SOD2
PGK1	ARHGAP21			GPSM2
MIA3	ARHGAP23			MBD1
SDC4	ARHGAP26			COL7A1
VPS35	ARHGAP27P1-BPTFP1-KPNA2P3			DUSP4
GALNT7	ARHGAP29			IDH2
NAP1L4	ARHGAP32			EMP2
SRSF10	ARHGAP35			C1R
COPB1	ARHGAP4			HAS2
DDX17	ARHGAP42			CD40
RBM39	ARHGAP44			TNFRSF10A
TAX1BP1	ARHGAP9			AC002480.1
ANKRD12	ARHGDIA			EXT1
LMBRD1	ARHGDIB			PARP8
CALM2	ARHGEF1			AC000120.3
SLC37A3	ARHGEF10			AP002990.1
RDX	ARHGEF10L			DHFR
ABCD3	ARHGEF16			HLA-V
CKAP4	ARHGEF17			AMOTL2
TMEM41B	ARHGEF25			DEPDC1
NFE2L1	ARHGEF3			SC5D
EPRS1	ARHGEF34P			ITPKC
UBE2Q1	ARHGEF37			NFKB1
ERBB3	ARHGEF5			CASP8
DIMT1	ARHGEF7			VTCN1
RAC1	ARHGEF9			KSR1
CALM1	ARID2			CDK12
PRSS23	ARID3A			RAB24
SFPQ	ARID3B			AURKA
EXOC3	ARID5A			AC068580.4
ADAM9	ARIH2			TUBA1A
XPO1	ARL10			RTN3
ACSL5	ARL13B			ITGAV
CCT2	ARL14EP			RPL3
KDELR1	ARL2			AEN
PCMTD2	ARL3			TTC28

ACTR2	ARL5B			IL10RA
ALDH3A2	ARL8A			PIK3R1
RTF1	ARMC10			POLD2
SH3YL1	ARMC5			TNFRSF9
ATP6V1A	ARMC6			ANLN
CBX3	ARMC8			AP5Z1
MAP2K2	ARMC9			NOL6
CLSTN1	ARMCX4			RYR2
ZDHHC13	ARMCX5-GPRASP2			RBM5
LAPTM4A	ARMCX7P			PTPRF
ANXA2	ARMH3			SGO2
SEPTIN2	ARNT2			NEK2
SLC6A6	ARPC1B			SHROOM2
CLTC	ARPC2			ZNF263
STIM2	ARPC3			GBP2
RCBTB1	ARPC5			ANKRD33B
PSMD14	ARPC5L			EID1
PCYOX1	ARPP19			PSAP
SACM1L	ARRB1			IQGAP1
ST14	ARRB2			DUSP10
ACVR1B	ARRDC1			LAD1
SEC61A1	ARRDC2			SFT2D2
SYNGR2	ARRDC3			SERBP1
IL13RA1	ARRDC4			PALMD
CAPZA2	ARSB			HSD17B14
XPO7	ARSD			TBL1XR1
CANX	ARSJ			GPCPD1
GALNT1	ARSL			GJB1
SDC1	ARX			HBEGF
MFSD14B	ASAP3			HACD2
DDX18	ASB1			ZNF385C
CCNDBP1	ASB13			ZC3H12C
ADGRF1	ASB16			USF1
UBE2N	ASB6			PCGF5
MDFIC	ASB7			MAGT1
SSR1	ASB8			SNHG12
CNOT8	ASCC2			FN1
SDCBP	ASCC3			ASAH1
RAB5C	ASF1A			TP53BP2
ERP44	ASL			MDK
MPZL2	ASMTL			LPP
HMGXB3	ASPSCR1			PFKFB3
RSRC2	ASS1			AC118553.2
KPNA1	ASXL1			CD55
HSPA9	ASXL2			SEMA3D
AKR1C3	ATAD3A			IFI44L
ALCAM	ATAD3B			LINC02701
CTNNB1	ATF1			LIPA
GAPDH	ATF3			NNT
GSN	ATF4			ANP32E
TFCP2L1	ATF6B			CIT
PDCD4	ATF7			ACAA2
DYNLT1	ATG101			JMJD1C
QSOX1	ATG16L1			SNPH
GOLGA1	ATG16L2			CD63
ESYT2	ATG2A			SARAF
HSP90AA1	ATG2B			ODAM
RBBP9	ATG4A			DARS2
AAGAB	ATG4B			MAP4
CD46	ATG4C			MED1

TAP1	ATG9A			SERINC5
HIGD1A	ATN1			SUCO
CLDN7	ATOX1			CXCL3
GM2A	ATP10A			PA2G4P4
KIF21A	ATP11A			PCLO
MARK3	ATP12A			FA2H
PAICS	ATP13A1			GALNT1
NFKB1	ATP13A2			DUSP8
HNRNPA2B1	ATP1A1			BCL2L14
EIF5B	ATP1A3			H3-3B
WEE1	ATP2A3			PPP2R3A
CDC42SE2	ATP2B1			LMAN1
USP48	ATP2B1-AS1			PSMA6
ANKRD36B	ATP2B4			NKX3-1
HNRNPL	ATP2C2			DEPP1
RCAN3	ATP5F1A			AHCYL2
ALDH3A1	ATP5F1B			GTF2B
SRPRA	ATP5F1D			SNIP1
FMO3	ATP5F1E			MORF4L1
CLCN3	ATP5IF1			ZNF697
UGP2	ATP5MD			SREBF2
NIPAL2	ATP5MF			PDXK
NCKAP1	ATP5MPL			H2BC14
RAB5A	ATP5PB			RPL7
SLC38A2	ATP5PD			HES4
EIF1AX	ATP5PF			LSS
DECR1	ATP5PO			HLA-DRB1
SLC25A3	ATP6V0A1			GPT2
SLC44A2	ATP6V0A2			ARHGAP11A
CP	ATP6V0A4			PRR15
PSAP	ATP6V0D1			SNORA68
GRN	ATP6V0E1			IGFBP4
SLC20A1	ATP6V1B2			MKI67
MARCHF5	ATP6V1C1			SEMA3E
IGFBP3	ATP6V1F			GPI
SON	ATP6V1G1			PLCH1
GOT1	ATP6V1H			ABCD3
SLC15A2	ATP7A			ERBB2
ACADM	ATP8B2			HK2
SLC30A7	ATRN			STC2
CCT5	ATRX			ZFP36L2
SORT1	ATXN10			KRT19
TMEM87B	ATXN1L			PPP1CB
VAV1	ATXN2			BAG1
PLRG1	ATXN2L			IER2
FBP1	ATXN3			CELSR3
EIF2S3	ATXN7L3			MASTL
IARS2	AUNIP			PDGFRL
TMBIM6	AUP1			RPL27A
MAOA	AURKAIP1			LIFR
HSPA5	AURKB			NOD1
B4GALT5	AUTS2			NT5DC2
COX6C	AXIN1			PAM
CHL1	AXIN2			C1S
MFSD1	AXL			PCYOX1
LINC01578	AZIN1			CLDN10
BUB3	B2M			H1-10
TRA2A	B3GAT3			STON2
ATP5PB	B3GNT2			CD68
POLR2B	B3GNT5			ATP1B1

ACSL3	B3GNT6			ADAM9
SRSF3	B4GALT2			SLIT2
DHX29	B4GALT3			RPS6
RAB1A	B4GALT7			IGF2
SCNN1A	BAALC			MYO1C
WASHC2C	BACE1			RFX5
IMPAD1	BACE2			NAMPTP1
GALNT12	BAG1			MNS1
LAMP2	BAG3			AFDN
ATXN10	BAG6			ABTB2
SPTLC1	BAHD1			BUB1B
FDX1	BAIAP2			NIBAN2
TM4SF1	BAIAP2L1			IL7R
TMEM50B	BAK1			LMO4
ATP5F1A	BANF1			AC083837.1
RBM6	BANP			SRI
B2M	BAP1			RN7SL1
NPC2	BARD1			SUMF1
PERP	BASP1			COL16A1
ELOC	BATF2			TSPAN6
ANXA3	BAX			LDLR
SLBP	BAZ1B			DENND4A
TMED10	BAZ2A			TMA7
CPA4	BAZ2B			HNRNPR
EFTUD2	BBOF1			FAM53C
LDHB	BBS1			NCMAP
MMUT	BBS12			OR52K3P
EEF1A1P6	BBS2			PSMA4
SRI	BBS4			MIR3609
LINC00511	BCAP29			PRC1
SSB	BCAP31			INSIG1
SKP1	BCAR1			USP5
AKIRIN1	BCAR3			LINC02542
DERL1	BCAS1			ZNF620
ATRX	BCAT1			AC132217.2
UNC5B	BCAT2			IRAK1
TMEM30B	BCDIN3D-AS1			ICAM1
SLC44A1	BCKDK			SLC25A28
LTA4H	BCL11A			HELB
SEPHS2	BCL2A1			MAP3K20
ACTG1	BCL2L1			VDAC1
IRF2BPL	BCL2L13			HLA-DMB
AQP5	BCL2L14			TGOLN2
NDUFS8	BCL2L2			PBXIP1
STT3B	BCL3			CDCA3
A4GALT	BCL6			SNHG1
TUBA1A	BCL7A			CHAC1
COPS8	BCL7B			OVOL1
NDUFA10	BCL7C			AIF1L
PRXL2A	BCL9			ARRDC4
GDE1	BCL9L			AC022034.1
DARS1	BCLAF3			ARC
ANXA1	BCOR			VCL
NUCB2	BCR			AL109918.1
ID1	BCS1L			ANXA11
FMO2	BDKRB2			ARSD
COG2	BECN1			VPS35
PSMC5	BEND7			LMNB1
RSRP1	BEST1			LYZ
NDFIP1	BET1L			C15orf48

B4GALT1	BEX1			AC107959.1
RPL6P27	BEX4			CPEB4
KIFAP3	BHLHE40			AKR1C3
F3	BICD1			FGFR3
KARS1	BICD2			PNRC1
OSTC	BICRAL			GRINA
IDH1	BID			ATP5F1A
JAG1	BIN1			RGMB
GLT8D1	BIN2			SNORA71A
KYNU	BIN3			AFF4
CMTM6	BIRC5			HSPB8
SDR16C5	BIRC6			SAR1A
EMB	BLCAP			SH3BGRL2
CCDC47	BLMH			RNF128
PROM1	BLOC1S1			AC025580.2
APP	BLVRB			GCNT4
MIPEP	BLZF1			CTDSPL
C2CD2	BMERB1			PAXIP1-AS2
TMEM33	BMP1			AC018644.1
CD164	BMP2			AC106886.6
MDH1	BMPR1AP2			DDB1
TFCP2	BMS1			AGO1
TMPRSS11D	BMS1P1			PLEKHB1
NR2F2	BMS1P17			ANXA9
VPS26A	BMS1P22			IKBKE
CAST	BMS1P8			AHCY
GSTK1	BNIP1			ALDH7A1
YWHAB	BNIP3P1			PRDX5
TXNDC17	BNIP3P11			MYC
CCN2	BOD1			OAZ1
CKMT1A	BOLA3			DAG1
PSMD11	BRAF			GNB4
PKM	BRAT1			IFRD1
METTL21A	BRCA2			LARS1
LAPTM4B	BRD1			ERRFI1
PMPCB	BRD2			MARCKS
PRNP	BRD3			HRH1
SEC63	BRD3OS			C4A
TMEM165	BRD8			PODXL
CAV2	BRD9			EZR
PYGL	BRI3			NCOA3
ARPC5	BRK1			ROR1
TMEM150C	BRMS1			SMARCC1
CD63	BRWD1			GPAT4
PLS3	BRWD3			PTPRS
HLF	BSDC1			PRR11
AC087473.1	BST1			CH25H
ATP6V0D1	BTBD1			ITGAM
PSMB3	BTBD19			PTMA
NOMO1	BTBD3			SQSTM1
GTF2E2	BTBD7			SAMD4A
OAT	BTF3L4			INO80D
CD151	BTF3L4P2			TRIO
ALDH2	BTG2			HLA-DPA1
FBXO3	BTN2A2			SLC44A4
MORN2	BTN3A2			CTNNA1
CTBS	BTNL9			RTL9
STOM	BUB3			MBNL3
RNF149	BUD13			SLC9A7
MAP3K5	BUD23			FTL

ERLIN1	BX248409.1			SLC25A25
ERMP1	BX284668.2			FOSL2
SMIM15	BX679664.1			RPS4X
CCDC80	BX679664.3			IL12A
HSP90B1	BYSL			SNRPD2
CKMT1B	BZW1P2			RBL2
TSPAN3	C10orf91			PDGFA
STAM2	C11orf1			CNN3
ABHD5	C11orf24			KIAA1217
MSMO1	C11orf49			TCN1
RBM5	C11orf71			ARID5B
TM9SF3	C11orf86			CHD8
ATP1B1	C11orf95			MPC2
SARAF	C11orf96			CASP7
AMFR	C12orf10			B3GNT5
DYNC2LI1	C12orf43			CHEK2
H3-3A	C12orf49			Z93241.1
RNF145	C12orf57			AC005515.1
ERG28	C12orf75			SLC26A2
TUBA1C	C14orf132			ZNF217
RBM3	C15orf39			AL162581.1
GLB1	C15orf48			RN7SK
PGD	C16orf58			SNORA49
USP47	C16orf91			HLA-K
TAGLN2	C17orf58			PIP5K1A
RETREG2	C17orf80			SYNPO
TMX4	C17orf97			NUSAP1
EXOC1	C18orf21			MSH6
MAT2B	C19orf25			TMEM59
VPS25	C19orf48			SPAG5
NUP107	C19orf84			DPYSL3
ADAM15	C1GALT1			STT3B
ARPC3	C1orf109			H2BC11
SYPL1	C1orf112			PFN2
TGFBR1	C1orf116			HTATSF1
DSG2	C1orf162			UQCRH
RPL4P4	C1orf194			PDGFD
SELENOP	C1orf198			CLINT1
EPCAM	C1orf21			AL121594.1
S100A6	C1orf216			PDE1C
COG6	C1QBP			SNORD10
ANKRD10	C1QL4			CUX1
WDR33	C1QTNF1			ZNF787
LRRC8D	C1QTNF6			DHRS2
MMP14	C1S			SCARNA9
RWDD4	C2			OPHN1
SDF4	C20orf194			ITPRIPL2
DLD	C20orf27			YWHAQ
FHL2	C20orf96			NXF1
SLC35F5	C21orf91			BMP2
TACSTD2	C22orf39			INPP4B
ASAH1	C22orf46			ARPP19
GUF1	C2CD2			DNAJA4
ENO1	C2CD4A			DNAJC22
LUC7L3	C2orf42			SCP2
MTDH	C3			SNAI1
JKAMP	C3AR1			KLF10
H2AZ2	C3orf38			CPEB2
ERAP1	C3orf62			LINC00472
TMEM192	C4A			PGRMC1

RCN1	C4B			OTUD4
WASH6P	C4orf3			MAP3K13
ITGB5	C4orf33			SCARNA12
SLC35A2	C5AR1			PLS3
TTC19	C5AR2			APOBEC3F
OCIAD1	C5orf30			STX19
ADAM28	C5orf51			APOL4
P4HB	C6orf120			USP43
SFN	C6orf132			C3orf52
SLC39A7	C6orf136			ROS1
EIF4A3	C6orf47			TPM4
TMEM30A	C6orf62			DDX39B
GPD2	C7orf25			ZNF441
CD59	C7orf26			HOMER2
GTF2H2B	C7orf50			RNF111
PON2	C8orf33			HLA-L
HACD3	C8orf34			HSD17B4
GSS	C8orf37			NCALD
TUFM	C8orf44			PLK3
ATP2C1	C8orf58			SLC39A10
CACHD1	C8orf82			PTP4A2
CA12	C9orf116			PHF11
MBOAT2	C9orf64			PIWIL4
THYN1	C9orf72			ADAM8
PDIA6	CA1			NFIA
LEMD3	CA12			RPS3A
RPN1	CAB39			ITGB5
PTTG1IP	CAB39L			CDK1
PRDX1	CABIN1			TMSB4X
GAA	CABLES1			CYP2B6
ABCC3	CACNA1A			AGRN
IER3IP1	CACNA1C			ERLIN2
EGFR	CACNG6			AC027290.2
ANXA4	CACNG7			ATP13A3
IGFBP2	CACTIN			CHMP3
SLC27A2	CACUL1			GSR
TRAPPC3	CAD			MOB3C
OS9	CADM1			RPL41
SLC18B1	CADM4			DNPEP
ALOX15	CADPS2			SBNO2
GNS	CALCOCO1			MMAB
RRN3	CALCOCO2			MSI2
PIGX	CALHM2			C15orf39
ARL6IP1	CALHM6			PRKAR2A
RPL7AP66	CALML3-AS1			TTC26
AC004069.1	CALR			CD83
LGR4	CAMKK1			ESYT1
LRG1	CAMKK2			AC245033.1
SPARCL1	CAMP			AC073548.1
TMEM87A	CAMSAP1			MAP2
ALDH1A1	CAMSAP2			TRA2A
LMAN2	CAMTA1			HS2ST1
AC007318.1	CAMTA2			PAICS
DDOST	CANT1			BTN2A1
CHPF	CANX			HNMT
CTSB	CAP1			PTPN11
RASA1	CAPN10			EPS8
SLC2A1	CAPN8			DEPDC1B
CFH	CAPRIN2			HDGF
TM9SF2	CAPZA1			GSN

EML3	CAPZB			UGGT1
CTSH	CARD16			CPSF1P1
HNRNPA1P4	CARD17			SRFBP1
ERLIN2	CARD6			SNORA21
MTRNR2L9	CARD8			ZNF627
LRP5	CARHSP1			AKAP8L
SEMA3A	CARS2			ZNF292
GFM2	CASC3			RCN2
HNRNPA1L2	CASC7			JPT2
PIGG	CASC9			AC007952.4
RRM1	CASKIN1			ACHE
TSPAN13	CASP1			ZNF704
TMEM68	CASP10			SAPCD2
KRT5	CASP4			RPS8
ACVR1	CASP5			LAMA2
ATP1A1	CASP7			MBTPS1
PRODH	CASP8			SRCAP
RPAP2	CASP9			ZNF317
MFSD11	CASTOR3			MLF1
FDFT1	CAT			PIEZO1
MFSD14C	CAV1			MRPL3
ANKRD36	CAV2			ARPC5
SGK1	CAVIN1			MYLK
NEU1	CAVIN3			NR4A1
CLDN4	CBFA2T2			CTH
SCGB2A1	CBFA2T3			AC093010.3
ERLEC1	CBL			ZNF442
EEF1A1P5	CBLB			ZBTB21
AL391121.1	CBLN3			SYDE1
POR	CBR1			ECH1
RAB3IP	CBWD5			JADE2
FAM3B	CBX4			HMGCS1
CALR	CBX6			ODF3B
AC073333.1	CBX7			NR2F1
EBPL	CBY1			SPAG17
NPTN	CC2D1A			H2BC18
HSD17B13	CC2D1B			HADHA
RPS21	CCAR1			ASNS
NIFK	CCAR2			STARD5
ALS2CL	CCDC102B			ZFYVE26
PSEN2	CCDC113			RPL5
RCN2	CCDC114			JUND
CHKA	CCDC115			GIGYF2
GTF2H2C	CCDC12			CASP4
TMA7	CCDC124			HIPK3
ALDH7A1	CCDC130			TBC1D22B
CD81	CCDC137			FLRT3
RTN4	CCDC14			IFT122
DHX36	CCDC148			CHSY3
SNRPA1	CCDC151			EBP
DNAJC1	CCDC184			LUCAT1
HNRNPA1P35	CCDC186			ACTN2
FTH1P10	CCDC22			AL358075.4
HNRNPAB	CCDC3			ARHGAP24
FUCA2	CCDC30			SPC25
ADK	CCDC38			ABLIM1
FMO5	CCDC43			SYNPO2
RTN3	CCDC57			DCLRE1C
ASCC3	CCDC59			CLK4
PSME1	CCDC60			LGALS3

WASH4P	CCDC66			IFNL4
TFB2M	CCDC68			CYFIP2
REEP5	CCDC69			TMED10
TMEM51	CCDC71			PAPOLA
WSB1	CCDC71L			ITGA6
SREK1	CCDC74A			MTF2
RTN3P1	CCDC84			IRF2
RARS1	CCDC85C			ETFB
HADH	CCDC86			RRM1
MBOAT1	CCDC88A			ATP5PB
PGAP4	CCDC9			C4B
FAAH2	CCHCR1			AC097625.2
SMG1P4	CCK			BDP1
AC008810.1	CCL2			SPATS2L
TLR2	CCL3L3			TMEM41B
ZMPSTE24	CCL4			IFT80
HNRNPA1P10	CCL7			CDKN2B
PAPSS2	CCL8			PLA2G4C
AGL	CCM2			CREBRF
TMEM9	CCN2			RPS24
TMEM9B	CCN5			MAFG
TSPAN1	CCNA1			ERMP1
CLN5	CCNB1			HEXD
TMEM59	CCND1			EGR2
AL592114.1	CCND3			TEX15
GUSB	CCNDBP1			AVPI1
ANAPC4	CCNG2			GCH1
COMMD7	CCNI			ALDH1A3
MAP3K6	CCNJ			RACGAP1
ANKRD66	CCNJL			DDX17
DSE	CCNL1			GPR160
GCLC	CCNL2			RUBCN
PRSS8	CCNT1			AC068831.8
CYP4F12	CCNT2			SPTLC2
ABCE1	CCNT2-AS1			NDUFV1
ENPP4	CCPG1			DDX5
ABCA5	CCR1			KIF14
GLUD2	CCR5AS			CCDC85C
CALM2P2	CCR7			FGFR4
KLHL42	CCRL2			ARPC2
PRMT7	CCS			KIF23
ITGB6	CCT4			PRXL2A
SESN1	CCT5			ST13
FTH1P2	CCT7			C1orf116
CAMK2G	CCT8			RPL6
STAM	CCZ1			OXCT1
MANF	CCZ1B			PCDH9
THOC3	CD151			RAB11A
MSTO1	CD164			CASP10
ZPR1	CD24			XRCC6
INPP1	CD248			USP13
NOMO2	CD27-AS1			ITM2A
LRRN1	CD2BP2			ATP6AP2
SRSF11	CD320			MDGA1
SELENOI	CD37			AC040162.1
TOPORS	CD38			ENAH
PLTP	CD4			TNF
SLC39A6	CD47			RALBP1
TMEM106C	CD53			KLK6
CD47	CD55			AC099489.1

SORD2P	CD59			CDC14A
AL158206.1	CD63			SLC38A1
KRT10	CD74			TANK
EIF4A1P4	CD82			EIF4EBP2
UNC93B3	CD86			NPLOC4
CXADR	CD8A			G0S2
CUEDC1	CD9			KIF15
IL10RB	CD93			FRYL
YWHAZP4	CD99L2			PCSK9
ATP5F1B	CDA			RBBP6
PRKAR2B	CDADC1			SLC16A7
SMC4	CDC14A			BTF3
PTDSS1	CDC16			AC005070.3
COPG2	CDC25B			PJA2
GTF2F2	CDC27			PPTC7
LONRF1	CDC34			LINC02432
RPL10P9	CDC37			TMEM167A
HLA-B	CDC40			CARHSP1
RFNG	CDC42			YEATS2
MAN1B1	CDC42BPA			ACTN1
EIF4HP1	CDC42BPB			RPS20
H3P36	CDC42EP1			ELF1
LACTB	CDC42EP2			TRAF4
CNIH1	CDC42EP4			NAALADL2
MAPKAPK3	CDC7			SNORA71D
JPT1	CDCA2			FAM168A
SMAP1	CDCA7			YRDC
CYP3A5	CDH11			ZBTB10
SPTSSA	CDH17			ARID3B
TTC29	CDH2			SAA2
C1D	CDH23			FAM168B
PA2G4P6	CDH5			ZBTB43
DPAGT1	CDHR4			CYB5A
SMARCAD1	CDHR5			PLBD1
RPL7AP6	CDIP1			CORO1C
BX679664.3	CDK10			RNVU1-31
PLS1	CDK11B			CERK
F11R	CDK13			AC022506.1
RMDN3	CDK17			PLK1
DDAH2	CDK18			IFI30
BCAP29	CDK4			COX7C
FSCN1	CDK5RAP1			MVD
AKAP1	CDK5RAP3			UHMK1
ITFG1	CDK6			PLEC
ARVCF	CDK7			NPFFR2
LOXL4	CDK9			RPS17
AC090498.1	CDKAL1			HDX
FAM3D	CDKL3			CWC25
HNRNPA1P7	CDKN1A			PVR
HNRNPA1P12	CDKN1B			MGAM2
RHBDL2	CDKN2AIP			MAZ
MPP7	CDKN2B			ZNF7
UBE2E3	CDR1			MAST4
SCPEP1	CDR2L			EREG
DNAJB11	CDV3			ARPIN
SDHA	CEACAM1			FNBP4
LAMB3	CEACAM3			HMGA1
VARS1	CEACAM6			ARL14EPL
NT5DC1	CELF1			FLNA
PBXIP1	CELSR1			SNORA57

TAP2	CEMIP2			TKT
HLA-F	CENPB			EEF1B2
CDH1	CENPC			TOR1B
PRELID1	CENPN			ATP5F1C
UPK1B	CENPT			ZNF92
PSMD1	CENPX			RBBP7
TMTC4	CEP120			ANGPT2
ITGAV	CEP164			NIPAL3
NUDT12	CEP170			SNORA74D
PGAM4	CEP19			LDHB
RNF26	CEP192			CAPZB
CCDC65	CEP250			HMGN3
FARSB	CEP295			MTHFD2
PARP2	CEP63			OLFM4
VIPR1	CEP68			COPA
ARL3	CEP83			IRS2
ENY2	CEP85L			SLC28A3
SDCBPP3	CEP95			SOX4
BCAP31	CERK			ERGIC1
B4GALT4	CERS2			HDAC1
RAMAC	CERS3			SUPT6H
C5orf15	CERS5			CBX3
FTH1P8	CERS6			HSCB
MAD2L1BP	CERT1			MTF1
RAC1P2	CFAP161			CDKN3
NAAA	CFAP20			CCND3
GLULP4	CFAP300			ANP32A
EXOSC8	CFAP36			ARHGAP27
MT-TY	CFAP52			ANGPTL4
BLOC1S4	CFAP58-DT			ACP6
AC144530.1	CFAP77			SNORA48
TMEM45B	CFAP97			ATF4
ABHD3	CFDP1			SLC3A1
HLA-L	CFL1			RNU4-2
AC113935.1	CFLAR			ICMT
PRPF39	CGB2			ATP5PD
CTNNAL1	CGB5			CCNG1
VPS35P1	CGN			AC020916.1
HSP90AB2P	CGRRF1			NDUFB6
UNC93B7	CHAC1			RACK1
CHPT1	CHCHD1			CHP1
TNC	CHCHD10			TUBA1B
DDX50	CHCHD2			PARK7
MLYCD	CHCHD3			SLC1A5
AQP3	CHCHD4			TRIB1
LRRCC1	CHCHD6			HGSNAT
TPBG	CHCHD7			DNM1
GJB3	CHD1			CYP1B1
PABPC1P4	CHD2			SYT16
SLC25A17	CHD3			PRKCI
SLC44A3	CHD4			AL049629.2
SLC26A4	CHD6			TRHDE
PRDX4	CHD7			AP2B1
GGH	CHD8			RPS9
HNRNPA1P8	CHFR			TJAP1
LSAMP	CHI3L1			CPXM1
CDS2	CHI3L2			WDR74
FAT1	CHID1			NIPSNAP2
ATP6AP2	CHIT1			ARHGDIB
MRAP2	CHKA			PROM1

G6PC3	CHL1			C6orf58
ERO1B	CHMP1A			BCAS1
CLK1	CHMP1B			CCNA2
SNX14	CHMP4B			RNU4-1
PTGES3P3	CHMP5			PLAAT2
CCT6A	CHMP6			LINC02605
AC006511.4	CHN1			KHDC4
KLF9	CHPF			TMEM181
FKBP1C	CHPF2			FSD1L
PIPSL	chr22-38_28785274-29006793.1			SH2B3
FAM162A	CHRAC1			ANXA1
AC024293.1	CHRNB4			KANSL1
TMEM147	CHROMR			WFDC5
ALG1	CHST10			SPTA1
CCDC59	CHST11			TGIF2
DBP	CHST2			TLCD4
TF	CHST6			PCM1
FKBP9	CHSY1			FRAS1
RPL14P1	CHTOP			CDC20
TMEM43	CHUK			GSPT1
ACAD10	CIAO1			VANGL1
BORCS5	CIAO2A			ARFGEF1
EMC7	CIAPIN1			TICAM1
ACTA1	CIB1			USP15
UNC93B6	CIC			LMO2
EDEM2	CICP23			KIF1C
PTK7	CIITA			CERT1
ARSDP1	CIPC			DSC3
TMED7	CIR1			FNBP1L
TUSC3	CIRBP			AKR1B10
PGRMC1	CISD2			THRA
UBLCP1	CISD3			GPR107
AC026271.1	CITED2			MTHFD1
BMI1	CKB			CALM1
DYNC1I2P1	CKS1B			CENPI
ARMT1	CLCA3P			RNU6ATAC
TLR1	CLCF1			CDK17
GPC1	CLCN2			PLEKHB2
VDAC1P1	CLCN3			NUDT21
HNRNPKP4	CLCN6			CTSE
RRAGA	CLCN7			NAP1L1
KIT	CLDN1			CHD3
ST13P3	CLDN2			CTNNB1
AC136632.1	CLDN4			AC013394.1
AL391244.2	CLDN5			SET
ETHE1	CLDN7			PFKFB2
TMED1	CLDND1			PLAC8
RPL7P10	CLEC1A			H1-5
HEXB	CLEC4A			IL15
MOSPD1	CLEC4E			GALNT2
SLC52A2	CLEC7A			AC087721.2
KDSR	CLIC3			RASGRF2
SELENOS	CLIC4			RNU2-2P
HLA-A	CLIP2			PER2
ADH1A	CLIP4			INO80
RPL22P1	CLK1			CACYBP
DNAJB9	CLK3			FARP1
AC083873.1	CLK4			TRIB3
DENND10P1	CLMP			ETFA
FUCA1	CLPP			CTTN

HSPA8P5	CLPTM1L			B3GNT2
LPCAT3	CLPX			MRPS16
SLC35B2	CLSTN1			SMC2
EXOSC9	CLSTN3			ATP5F1E
TAGLN2P1	CLTB			PHKB
ATP5MC3	CLUH			HARS1
EIF3FP3	CLUHP3			ATL3
COL6A3	CMAHP			GIMAP2
CHCHD1	CMC1			TFPI
TMCO1	CMC2			SLC30A9
PABPC3	CMIP			CKAP5
TP63	CMPK2			NDUFA4
CTSC	CMTM3			SNORD3B-1
HSD17B12	CMTR2			RPL35A
CEACAM3	CMYA5			BCL3
SMG1P1	CNBP			TTLL11
PSMC1P1	CNGB1			HNF4A
ELMO3	CNIH4			TBC1D5
AL158801.6	CNKSR1			DIAPH2-AS1
UQCRC1	CNOT1			G6PD
HLA-V	CNOT2			CEP55
MGST1	CNOT3			KLHL29
NTS	CNOT7			AC104389.5
AL133477.1	CNPY2			RPS21
H3P44	CNRIP1			SCARNA7
PSENEN	CNST			PLEKHG7
RPL7AP34	CNTLN			ZNF207
PSPC1	CNTN1			DESI1
LAMC1	CNTROB			AK4
AL121769.1	COA5			GM2A
NDUFA12	COASY			VILL
MT-TL1	COG1			H2BC6
AC106795.1	COG3			LMCD1
AC022968.1	COG4			OR4C6
LRRIQ1	COG6			PLOD2
LRRC17	COG7			TSPAN13
UFD1	COL12A1			EIF4B
UXS1	COL16A1			PPP2R5D
AC008065.1	COL1A1			MFSD12
KRT6B	COL20A1			TNFRSF1B
HMGN2P17	COL4A1			SPNS2
ALOX15P1	COL4A4			PGD
RPL37AP1	COL5A1			PRDX3
ATP6AP1	COL5A2			SEPHS1
AC099670.1	COL5A3			CBX5
BMP3	COL8A1			TCF12
FKSG70	COL9A2			ACTN4
FAM171A1	COLGALT1			ACSF2
RPN2	COMMD10			ANKRD10
HLA-H	COMMD3			ARHGAP44
EEF1A1P38	COMMD4			PARP3
THNSL2	COMMD7			ERVK3-1
PDIA3	COMTD1			SLC25A6
AC064799.1	COP1			LYST
KTN1	COPA			CAV1
AC209007.1	COPB1			ATP11B
AC104619.3	COPE			PRDX2
SYT8	COPS3			SPRY4
COQ5	COPS5			PPP1CA
MSH2	COPS7A			SNORA23

NPC1	COPS7B			NIN
UQCRFS1P1	COPZ1			CMPK1
MORF4L1P1	COPZ2			CDCA7
UBBP4	COQ2			SNORA54
GPR89A	COQ4			TNNT1
MFSD5	COQ8A			ZNF689
SERPINB4	COQ9			XPO6
HNRNPA1P48	CORIN			AC010197.2
AP000936.3	CORO1C			GPC4
SIRT3	COX10-AS1			ZNF300P1
AC092115.2	COX16			AC124319.3
RPS27P29	COX18			FKRP
CYP26A1	COX4I1			SELENOF
ATP13A5	COX5A			MBOAT2
AL109918.1	COX5B			TTN
PIGO	COX6A1			TRIOBP
ITM2B	COX6A1P2			GEM
RPL9P32	COX6B1			PPP2R5C
PFN1P1	COX6C			TPGS2
EIF2S2P4	COX7A1			PBK
HNRNPA3P6	COX7B			SHMT1
HSP90AB3P	COX7C			GNPNAT1
PLLP	COX8A			NECTIN3
CLDN1	CPAMD8			PHC2
ANXA8L1	CPD			RNF144A
RPL23P8	CPE			CLMN
AC113404.3	CPLX1			SERF2
HSP90AA6P	CPLX2			ATP8B1
RPL7P47	CPNE3			LCP1
SETP20	CPNE8			DBN1
PHF14	CPSF1			AKIRIN1
SRD5A3	CPSF3			FYB1
SCAMP3	CPSF4			LMO7
AC244034.1	CPSF6			NIPSNAP1
TRIAP1	CPSF7			LMNB2
F2RL1	CR1			NRIR
PPIAP87	CR392039.1			SLC9A8
AP002784.2	CRABP2			SERPING1
SUMO2P1	CRACR2B			ATP5MG
AC005000.1	CRAMP1			GSDMD
HLA-C	CRAT			ECE1
MTATP8P1	CRCT1			TCIM
KRT18P16	CREB3			CXADR
SERBP1P5	CREB3L2			MAFA
RPL4P5	CREBZF			SUMF2
DCAF13	CRELD2			DTYMK
SRSF2	CREM			SLC25A5
SETSIP	CRIP2			ATRAID
FKBP9P1	CRIPAK			ARHGAP5
PRCP	CRISP3			ELK4
CD9	CRK			APPL1
IFNGR1	CRNDE			MORF4L1P1
RPL7P9	CROCC			LPGAT1
TMEM212	CRTC2			KAT6A
EIF4BP3	CRTC3			RIPK1
PSCA	CRY2			NPNT
AL049597.1	CRYAB			CCDC18
RPL7P32	CRYBG3			AC079781.5
YWHAZP5	CRYL1			HEXIM1
AC012085.1	CRYZL2P			AC008038.1

HNRNPCP2	CS			DCAF11
FTLP3	CSDE1			CDCA7L
APLP2	CSE1L			AC016831.1
SETP14	CSF2RA			RANBP3L
UBE2I	CSF2RB			MYL6
CDC42P6	CSF3R			MGAT4B
ANXA8	CSNK1A1			RAPGEF5
AL354702.1	CSNK1D			GLUD1
FGFR3	CSNK1E			SSRP1
H3-5	CSNK2A2			VDAC3
TSPAN6	CSNK2B			FANCD2
EIF4A1P2	CSPG4			TNS3
RPL7P1	CSRNP1			CDC42
GAPDHP65	CST7			IRS1
AC105250.1	CSTA			NKILA
EPHA1	CSTB			TMEM132A
AC002075.2	CSTF2			SH3TC1
RPS7P10	CTA-342B11.3			MICB
EEF1A1P19	CTBP1			CCDC88A
EIF5AL1	CTBP1-DT			MIR100HG
RPL10AP2	CTBS			AC245014.3
ARSD	CTC1			LSM3
XRCC6P2	CTCF			RNU12
RPL13AP20	CTD-2369P2.2			CALM2
AC092597.1	CTD-2521M24.9			SLC25A22
EIF4BP6	CTD-3195I5.1			COA3
AC099560.2	CTDNEP1			LCLAT1
RPL12P38	CTDP1			MMD2
RPS26P6	CTDSP1			ADIRF-AS1
LDHBP2	CTDSP2			HTATIP2
KRT18P11	CTDSPL			WASHC5
AC004057.1	CTDSPL2			MCM6
RPL34P26	CTF1			CENPA
BZW1P2	CTNNA1			LSM4
AC092683.1	CTNNB1			ERN1
S100A4	CTNNBIP1			DLC1
HLA-DRB6	CTNNBL1			SETD2
C1GALT1C1	CTNND1			SERTAD1
RPS26P31	CTPS1			GBF1
SLC5A8	CTPS2			FZD8
MTCO1P40	CTR9			MARCKSL1
GAPDHP44	CTSA			ANO9
RPL7AP11	CTSC			ACTL6A
PPIC	CTSE			OIP5-AS1
TMX1	CTSH			FAM20B
ITGA6	CTSL			CCR1
GAPDHP61	CTSS			OAF
PSMC1P5	CTSZ			SP140
EIF4BP7	CTTN			PRSS12
PPIAP16	CTXN1			ZUP1
EEF1A1P7	CUL1			RPL18
RPSAP19	CUL4A			ZNF3
AC005480.2	CUL9			CYB5B
UNC50	CUTALP			BCL2L13
MIR22HG	CWC22			STK19
RPS26P8	CWF19L2			SYNE2
AC115223.1	CXCL10			ZBTB38
HSPA8P1	CXCL11			DSC2
DPYD	CXCL16			JUP
AC126120.1	CXCL17			MEG8

AC016734.1	CXCL2			CD38
RARRES1	CXCL6			PAH
MTCO3P12	CXCR2			CXXC5
DPY30	CXorf21			GNPAT
ALG5	CXorf40A			RNPEP
PPIAP66	CXorf40B			EXOC3L4
PPIAP43	CXXC1			GANAB
AC112187.1	CXXC5			AC084816.1
PPIAL4C	CYB561			E2F7
MTND4P12	CYB561A3			SCARB2
TMEM183B	CYB5A			KIF12
ACTBP2	CYB5B			GNAS
HLA-G	CYB5D2			STRBP
RPS7P11	CYB5R1			VMA21
NACA3P	CYB5R2			MFSD2A
EEF1A1P29	CYBB			IFNL3P1
AC068522.1	CYBRD1			LRRC20
RPS26P11	CYC1			TUFT1
AC034236.1	CYFIP1			APEH
MTCO2P2	CYFIP2			HOGA1
AC020898.1	CYHR1			TUBB
CLCA4	CYP19A1			SLC16A9
LYPD3	CYP1A1			ATP5MPL
EEF1A1P4	CYP24A1			USP25
AC004552.1	CYP2J2			MYO9A
NAMPTP1	CYP2R1			MICAL3
PPIAP13	CYP2S1			MAPRE1
RPS4XP22	CYP3A5			TPI1
EEF1A1P25	CYP4F11			SFSWAP
TUBAP2	CYP4F3			UROD
CROT	CYP7B1			LONP2
AP000281.2	CYREN			TLK2
RPL10P12	CYSLTR1			SIX4
FTH1P15	CYSTM1			GALNT12
RPL3P4	CYTH1			TNFRSF10D
AC104339.1	CYTH3			RNVU1-27
RPS23P8	CYTIP			BTC
PPIAP31	DAAM1			CHROMR
AC135178.7	DAAM2			C5
RPL10P4	DAB2IP			NR4A3
AC104563.1	DACT1			KNL1
RPS26P47	DAG1			IFNAR1
H3P16	DAGLB			ZFC3H1
FTH1P11	DANCR			GAS5
EEF1A1P16	DAP			TRIM2
YWHAZP3	DAP3			EIF4A1
GAPDHP73	DAPK2			ZHX2
GAPDHP63	DAPK3			CREBL2
AL627402.1	DAPP1			AGR2
AC009245.1	DARS1			AL645929.1
RPS3AP5	DARS-AS1			MAD2L1
H3P6	DAXX			DNAH9
RPL27AP5	DAZAP1			HNRNPUL2
RPS26P15	DAZAP2			KRT7
H3P47	DBF4B			UBASH3B
AC078819.1	DBN1			FBN2
TCN1	DBNDD1			STIM2
HSP90AA2P	DBNL			DIRAS3
FTH1P7	DBR1			OR1F1
RPS7P1	DCAF10			PRKAR1A

RPS27AP16	DCAF11			TMPO
RPL7P19	DCAF13			C11orf58
TMSB4XP2	DCAF15			DMBX1
RPS26P28	DCAF16			RPS10-NUDT3
RPL10AP6	DCAF4			TNRC6A
PPIAP22	DCAF5			GRIP2
EEF1A1P8	DCAF6			FYB2
ADH1B	DCAF8			JAKMIP3
ATP1B3	DCAKD			TC2N
PPIAP6	DCBLD1			CHSY1
RPL3P2	DCHS1			GABRE
RPL13AP25	DCLRE1A			IL22RA1
MTND6P4	DCP1A			MARS1
RPS24P8	DCP1B			KLHL3
AC092865.1	DCPS			VAMP5
RPL15P20	DCTD			RUFY4
AL133260.1	DCTN2			ACOT13
FTH1P16	DCTN4			TM7SF2
EEF1A1P11	DCTN5			ASPH
RPS15AP1	DCTN6			GRWD1
FTH1P3	DCUN1D3			NECAP1
APOD	DCUN1D4			FEM1A
AL596275.1	DDAH1			KMO
RPL15P18	DDB1			PKP4
RPL17P22	DDB2			GGCT
KRT6C	DDHD2			AC091133.1
EIF4A1P10	DDIT4			ADPRHL2
PDIA3P1	DDOST			FAM149A
FTH1P20	DDR2			AMER1
EEF1A1P22	DDT			LNPK
ANXA2P2	DDTL			SESN3
AC073072.1	DDX1			TNFSF9
AC025518.1	DDX17			AC015849.1
S100A2	DDX23			U62317.1
RPL7P23	DDX31			TMEM41A
MTND6P3	DDX39A			IK
AL009174.1	DDX39B			PBX1
AC092670.1	DDX41			SNORD13
PPIAP29	DDX42			B4GALT4
FTH1P12	DDX5			NOTCH3
AC090543.3	DDX51			TMEM245
H3C9P	DDX54			CSDE1
RPL17P36	DDX55			AIFM2
MTND5P11	DDX56			NUP62
MT-TE	DDX58			ANAPC16
MTCO2P12	DDX6			CEP57
MTRNR2L1	DDX60			NYNRIN
HLA-J	DDX60L			AL355075.4
EEF1A1P12	DEAF1			RPPH1
EEF1A1P14	DEF6			PPIAP22
AC091429.1	DEF8			GBP7
FTH1P5	DELE1			GMFB
TPT1P9	DENND11			YWHAE
EEF1A1P13	DENND2A			HMGXB3
AC006386.2	DENND2B			EGLN1
AC012005.1	DENND2C			UGT1A10
RPL41P2	DENND2D			ARL4A
MT-TA	DENND3			G3BP2
	DENND4B			AL021707.6
	DENND4C			HOOK1

	DENND6A			AC008760.2
	DENR			SERPINA4
	DEPDC5			HNRNPU
	DEPP1			FUT8
	DERL2			TLN1
	DESI1			ZNF433
	DESI2			IPO8
	DGAT1			SAMD1
	DGCR2			CPNE2
	DGCR6			WDR75
	DGCR8			RMND5A
	DGCR9			IL1RN
	DGKD			AL138787.2
	DGKE			TTYH3
	DGKG			ADD3
	DGUOK			MRPL24
	DHCR24			OBSL1
	DHDDS			ZNF44
	DHODH			ZNF532
	DHPS			DENND4B
	DHRS12			NBEA
	DHRS4L1			EEF1A2
	DHRS4L2			RNF5
	DHRS7			CDC25C
	DHRS9			HGD
	DHRSX			SBNO1
	DHX15			LSM5
	DHX16			SMCR8
	DHX29			ZSWIM8
	DHX30			PDCL3
	DHX32			IL11
	DHX35			KLHDC7A
	DHX38			DUSP2
	DHX57			OPA1
	DHX8			BCAR1
	DHX9			UPF1
	DIAPH1			THUMPD3-AS1
	DIDO1			AL591222.1
	DIP2A			RAD21
	DIP2C			CXCL9
	DIPK2A			AL645929.3
	DIS3			SNRPD3
	DIS3L2			SPATA2
	DISP1			RPS10
	DKC1			KLC4
	DKK1			HP1BP3
	DKK3			DNMBP
	DLC1			EPB41L1
	DLEU1			KHNYN
	DLG4			ACLY
	DLGAP1-AS1			ITGA5
	DLL1			POLR3D
	DLL3			RAET1L
	DLL4			PLAT
	DLST			CALU
	DLX1			PYGB
	DM1-AS			ZEB1
	DMAC2			AC083862.2
	DMBT1			RECQL5
	DMBX1			ZNF222

	DMKN			LEPROT
	DMPK			PXMP4
	DMXL2			ARHGAP42
	DNAAF1			SLC39A7
	DNAAF2			UNC119B
	DNAAF3			PSMA7
	DNAH1			COPS9
	DNAH14			SNRNP200
	DNAH7			PRR3
	DNAJA3			ATP2C1
	DNAJB12			ADH5
	DNAJB14			HDAC2
	DNAJB2			GATA6
	DNAJB5			RAPGEF2
	DNAJC10			SNCA
	DNAJC11			SYAP1
	DNAJC16			TBCC
	DNAJC18			MAD2L1BP
	DNAJC2			HK1
	DNAJC3			MIER3
	DNAJC4			CDC42BPA
	DNAJC5			CCDC34
	DNAJC7			ERI3
	DNALI1			EP300
	DNASE1			UGT2B7
	DNM1P51			ZFAS1
	DNM2			DYNC2H1
	DNMBP			ZFX
	DNMT1			ODF2
	DNPEP			GET4
	DNTTIP1			LINC00641
	DNTTIP2			LINC00265
	DOCK1			LRRFIP1
	DOCK10			CHCHD2
	DOCK2			IKBIP
	DOCK4			JAZF1
	DOCK5			HSBP1
	DOCK6			WIF1
	DOCK8			OR10AB1P
	DOCK8-AS1			FNIP1
	DOCK9			NR4A2
	DOHH			BBX
	DOK1			EXPH5
	DOK3			FARSB
	DOK4			LYSMD2
	DOLK			FH
	DONSON			IER3
	DOP1A			MIR6891
	DPF2			RNVU1-15
	DPH1			DYRK1A
	DPH2			HSP90AA1
	DPH5			SEPTIN11
	DPH7			MIR320A
	DPM1			ACOX1
	DPM3			RPS23
	DPP7			DCAF6
	DPT			TRPC4
	DPY19L1			RBBP4
	DPY30			LRRN3
	DPYSL2			HFE

	DPYSL3			TENT5C
	DPYSL5			CMBL
	DRAP1			KMT2E
	DRG1			SEC24D
	DROSHA			MMUT
	DSCC1			RPL15
	DST			AAR2
	DSTN			C2CD4A
	DSTYK			SLC9A1
	DTWD1			ZBED5-AS1
	DTX2			SMARCC2
	DTX3			RNVU1-6
	DTX3L			EIF3L
	DTX4			NQO1
	DUOX1			FAM107B
	DUOX2			RANBP17
	DUS2			REPS2
	DUS3L			BCL10
	DUSP1			SYT12
	DUSP10			QARS1
	DUSP16			LMAN2
	DUSP18			SUPT7L
	DUSP23			SND1
	DUSP3			HOXB6
	DUSP7			CD109
	DUSP8			PLEKHA2
	DUX4L2			SSTR2
	DUX4L5			TREX1
	DUX4L6			NDC1
	DUX4L7			HLA-G
	DUXAP9			SNORA80B
	DVL1			AKT1S1
	DYM			ANKFY1
	DYNC1H1			RHBDF2
	DYNC2LI1			ZNF629
	DYNLL1			H2AC12
	DYNLRB1			C12orf49
	DYNLT1			LEKR1
	DYRK2			THEM6
	DYRK3			TACC1
	DYSF			CCDC117
	DZIP1L			CCDC174
	E2F4			DNAJA1
	E2F8			NUP98
	E4F1			SCCPDH
	EBNA1BP2			PHKA2
	EBP			RPS19
	ECE1			METRNL
	ECHDC1			TIPRL
	ECHDC2			CBX4
	ECHDC3			MAP4K5
	ECHS1			PSMD10
	ECI2			PIGK
	ECM1			TMEM97
	ECM2			PTPRB
	ECPAS			EFNA1
	ECSIT			ADPGK
	EDC3			UQCRQ
	EDC4			HSPA6
	EDEM2			PPM1L

	EDF1			PSORS1C1
	EDNRA			DGLUCY
	EDRF1			SMARCA5
	EED			DAZAP2
	EEF1A1			TPD52L1
	EEF1A1P5			BSDC1
	EEF1A1P6			PIP4K2B
	EEF1AKMT1			E2F8
	EEF1AKMT3			NCAPD3
	EEF1B2			PRPF8
	EEF1D			VPS13C
	EEF2			COPG2
	EFCAB11			G2E3
	EFEMP2			NFX1
	EFHC1			CHD1
	EFHD2			TTC3
	EFL1			SWAP70
	EFNA1			FDCSP
	EFNA2			PPFIBP1
	EFNB1			AF127936.5
	EFNB2			PPP2R5A
	EFTUD2			AC068299.2
	EGFL7			LINC02044
	EGFLAM			HCFC1R1
	EGLN1			HAPLN3
	EGLN2			SCARNA21
	EGR1			TASOR2
	EGR2			EHMT2
	EHBP1			ASAP1
	EHBP1L1			STRAP
	EHD2			TTC39C
	EHD4			GTSE1
	EHMT1			BRAP
	EIF1			UFC1
	EIF1AD			RASAL1
	EIF1B			BRPF1
	EIF2AK1			KCNN1
	EIF2AK2			AL645608.7
	EIF2AK4			EIF4H
	EIF2B1			MMGT1
	EIF2B4			H2AC13
	EIF2B5			ZFP36L1
	EIF2D			SKAP2
	EIF2S3			DENND5A
	EIF3A			AKT3
	EIF3B			HCG4B
	EIF3C			AP1M2
	EIF3CL			TUBB6
	EIF3D			KIAA1549L
	EIF3F			LRCH3
	EIF3G			PDHA1
	EIF3H			URI1
	EIF3J-DT			MVP
	EIF3K			H4C8
	EIF3L			PAIP2B
	EIF4A1			AASS
	EIF4A2			MYORG
	EIF4A3			SYTL5
	EIF4E2			PINLYP
	EIF4E3			HEATR9

	EIF4EBP1			FILIP1L
	EIF4ENIF1			ARFIP2
	EIF4G1			GATA4
	EIF4G3			KNSTRN
	EIF4H			PLA2R1
	EIF5A			STEAP4
	EIF5AL1			SOCS2
	EIF6			RUVBL1
	EIPR1			SMARCA1
	ELAC2			SFXN3
	ELF1			RSU1
	ELF2			INTS5
	ELF4			UACA
	ELK1			CD69
	ELK3			STK38
	ELMOD2			BROX
	ELMOD3			ANKRD1
	ELMSAN1			SREK1
	ELOB			RBBP8
	ELOVL1			HLA-DRB5
	ELP1			HSPA2
	ELP3			TNIK
	EMB			RPL31
	EMC3			MAPK3
	EMC4			PKN1
	EMC7			NADSYN1
	EMC9			TTL
	EME2			ACSL4
	EMG1			STX16
	EMILIN2			ATP5MF
	EML3			RPLP0P6
	EMP1			CEP70
	EMP2			PPL
	EMP3			F3
	ENC1			DDX52
	ENDOD1			PAPSS1
	ENDOV			EXOC4
	ENG			WDR73
	ENGASE			C18orf25
	ENKD1			AL137077.2
	ENKUR			IGF2BP3
	ENO1			SELENOK
	ENO2			TRAK2
	ENO3			POLR2B
	ENOSF1			NR1D2
	ENOX2			CADPS2
	ENSA			WDFY1
	ENTPD4			PARPBP
	ENTPD6			RAB1B
	ENTPD7			STK38L
	ENTR1			MAVS
	EOMES			ARMC10
	EP400			CCDC167
	EP400P1			ARHGEF9
	EPAS1			RAB8B
	EPB41L1			HELLS
	EPB41L2			FBXO6
	EPB41L4A			SNX14
	EPB41L4A-AS1			GGA3
	EPC1			KDM5A

	EPDR1			PXK
	EPGN			RPS3AP6
	EPHA2			EIF3E
	EPHA4			EXTL3
	EPHB4			TMEM230
	EPM2AIP1			NR1D1
	EPN1			PSMD2
	EPN2			STAT3
	EPN3			CCNT2
	EPOP			SRP9
	EPOR			ZNF267
	EPS8L2			SPINK1
	EPS8L3			SMOX
	EPSTI1			PIMREG
	ERBB2			GALM
	ERC1			RABGAP1L
	ERCC1			LAPTM4B
	ERCC5			MYEOV
	ERGIC1			TNFAIP8L1
	ERH			RAB10
	ERI1			NRDC
	ERN1			APPL2
	ERP44			MXRA5
	ERRFI1			H4C2
	ERVK13-1			AC012020.1
	ESF1			STARD8
	ESS2			PLAU
	ESYT1			SNORA84
	ETS1			MIR3651
	ETS2			CREBBP
	ETV1			DNAJC10
	ETV3			NOSTRIN
	ETV6			AC243919.1
	EVA1C			ANO10
	EVI2B			MIB1
	EVI5			ZNF140
	EVL			CLIP1
	EWSR1			MAP4K3
	EXO1			PDK1
	EXOC2			NSDHL
	EXOC3			TRERF1
	EXOC4			AC140912.1
	EXOC6B			HLA-DMA
	EXOSC3			DHX40
	EXOSC4			SCD5
	EXOSC7			ANO6
	EXOSC8			TOMM20
	EXOSC9			TSPAN17
	EXT2			SEC14L2
	EXTL3			AC008079.1
	EYA1			PCDH1
	EYA3			UBE2C
	EZR			GRIN3A
	F11R			TRHDE-AS1
	F2R			AC119150.1
	F5			PCNP
	F8A1			C19orf48
	F8A3			LY75
	FAAH2			SNHG20
	FAAP100			RIMKLB

	FABP3			DDIT4
	FABP6			ZFPM2
	FADS1			RDH11
	FADS3			CENPU
	FAHD2A			EPS8L2
	FAHD2B			MCCC1
	FAM104A			SPSB1
	FAM110B			COL8A1
	FAM111A			CHPF2
	FAM114A1			ZDHHC2
	FAM118A			PIGA
	FAM118B			FAM122C
	FAM120B			MEGF6
	FAM122A			IP6K2
	FAM122B			ABCF1
	FAM126A			TMEM19
	FAM126B			SEC24A
	FAM131A			NCAPH
	FAM135A			AGPS
	FAM13A-AS1			AP5B1
	FAM13B			HSD17B12
	FAM151B			EDC3
	FAM156A			EBF4
	FAM156B			SEPTIN10
	FAM157B			MIPEP
	FAM157C			TESK2
	FAM160A2			UQCRB
	FAM160B1			NUMA1
	FAM160B2			COMMD9
	FAM162A			HAS3
	FAM167A			RBMS3
	FAM168A			HAVCR1
	FAM168B			SESN2
	FAM171A2			RUVBL2
	FAM171B			PLCE1
	FAM174B			VBP1
	FAM174C			PPIP5K2
	FAM177A1			VEGFA
	FAM177B			FKBP9
	FAM183A			TEX261
	FAM189A2			KIAA0930
	FAM193A			UBE2Q2
	FAM198B-AS1			NEK6
	FAM199X			AC008957.3
	FAM207A			ENC1
	FAM20B			NCAM1
	FAM20C			IARS1
	FAM214A			ZNF562
	FAM214B			HELZ
	FAM219B			HMGB1P5
	FAM220A			PTPRU
	FAM222B			SNRPE
	FAM231D			TMEM219
	FAM234A			FAM169A
	FAM234B			DPY30
	FAM32A			CARD16
	FAM3A			LCORL
	FAM3C			ANKRD17
	FAM49A			ATRIP
	FAM49B			CEBPD

	FAM50A			NMRAL2P
	FAM53B			LSR
	FAM72A			DENND3
	FAM72B			PEBP1
	FAM83B			AC138811.2
	FAM83G			METTL7B
	FAM86JP			RLF
	FAM8A1			NDST1
	FAM92A			NPC2
	FANCA			FAM43A
	FANCC			CTNND2
	FAR2			TMC4
	FARP1			MYO6
	FARP2			IRGQ
	FARS2			OSBPL8
	FARSA			IRF6
	FARSB			NFE2L2
	FAS			RNU1-2
	FASN			SNHG15
	FASTK			NEDD8
	FASTKD2			PEX16
	FASTKD5			MGST3
	FAT4			CEP78
	FAU			RELA
	FAXDC2			BX119927.1
	FBH1			PATJ
	FBL			HADH
	FBLN1			RNU4ATAC
	FBLN5			DNTTIP2
	FBN2			SMG7
	FBRS			OAZ2
	FBXL12			NAV3
	FBXL15			DHX30
	FBXL3			CCDC47
	FBXL4			FYTTD1
	FBXL5			DMAC1
	FBXL7			RSF1
	FBXO21			ID3
	FBXO25			KCNH8
	FBXO27			RPL34
	FBXO31			COX7A2
	FBXO32			BLVRB
	FBXO39			RPL22
	FBXO42			SGK2
	FBXO6			UBXN6
	FBXO9			PMVK
	FBXW11			STRN4
	FBXW2			AC010733.2
	FBXW4			AFF1
	FBXW5			SPDEF
	FCER1G			RNF11
	FCGR1A			PSAT1
	FCGR1B			ACTG1
	FCGR2A			ANKRD9
	FCGR2B			NDC80
	FCGR2C			SDCBP2
	FCGR3A			CDKN2AIP
	FCHO2			LYN
	FCRLA			LIG1
	FDCSP			STK32A

	FDFT1			CHML
	FDPS			TYMS
	FEM1C			IMPDH1
	FEN1			RNVU1-29
	FER			AC007192.1
	FERMT2			KPNA1
	FES			AL365436.2
	FEZ1			DSTN
	FFAR2			FANCI
	FGD4			QKI
	FGD5			KMT2B
	FGD5-AS1			KCNC4
	FGF1			PPCS
	FGF2			LAMTOR5
	FGF5			ANKLE2
	FGF7P6			ILRUN
	FGFR1			COX7A2L
	FGFR1OP2			PRKCA
	FGFR2			CNOT6LP1
	FGFR4			HMGB2
	FGG			NDUFS1
	FGL1			ALDH3A2
	FGR			ANKS6
	FHL1			SIK2
	FHL3			MAP3K14
	FHOD1			AP002381.2
	FIBIN			TMEM9
	FIBP			RPS7P1
	FILIP1			SLC7A5
	FIP1L1			SLC35G2
	FIS1			CIAO2A
	FIZ1			LINC01963
	FJX1			CCN1
	FKBP10			WAC
	FKBP14			SLC44A2
	FKBP1A			STT3A
	FKBP1C			RPS18
	FKBP4			VGLL1
	FKBP8			ANK3
	FKSG62			BBS9
	FKTN			ITSN2
	FLAD1			RPTOR
	FLG			SUCLG1
	FLG2			BZW1
	FLI1			STX10
	FLII			TINF2
	FLNA			TSPYL2
	FLNB			SLC25A23
	FLNC			C11orf96
	FLOT1			LAMC1
	FLRT2			FGD5-AS1
	FLT1			AKAP13
	FLT4			BAHCC1
	FLVCR2			AC069224.2
	FLYWCH1			MGST1
	FLYWCH2			SAA1
	FMR1			RPL13
	FNBP4			AC016831.6
	FNDC3B			USP11
	FNDC4			ALDH1A2

	FOS			KDM7A
	FOSB			BCAP31
	FOSL1			SNORD3C
	FOXA1			RAB5B
	FOXD1			MYOM1
	FOXD3-AS1			SQOR
	FOXE1			GORAB
	FOXJ2			THBS1
	FOXJ3			COPB2
	FOXK2			INS-IGF2
	FOXN2			CYBA
	FOXN3			USH1C
	FOXO1			OSER1
	FOXO4			PPARA
	FOXP4-AS1			RBM33
	FOXRED1			STK39
	FOXRED2			C12orf75
	FP236383.1			EPC2
	FP236383.9			TACC3
	FP565260.1			CAP1
	FP565260.3			SLC40A1
	FP565260.6			RAN
	FP671120.2			CKAP4
	FP671120.6			GPATCH8
	FPGS			AP000873.2
	FPR1			MPHOSPH9
	FREM2			GGH
	FRMD4A			ACP2
	FRMD6			BCO1
	FRMD8			TRAPPC6A
	FRS2			DNPH1
	FRYL			RNF114
	FSCN1			PNPLA2
	FSIP2			LATS2
	FSTL3			TPRKB
	FTH1			SORBS2
	FTH1P10			SNRK
	FTH1P11			HOMER1
	FTH1P12			RHOA
	FTH1P15			AL590867.2
	FTH1P16			CARS1
	FTH1P2			REEP3
	FTH1P20			IGF2-AS
	FTH1P23			CD151
	FTH1P3			MIR22HG
	FTH1P4			TYW5
	FTH1P5			LAMB3
	FTH1P7			RN7SL396P
	FTH1P8			PROCR
	FTL			KRT23
	FTLP3			KIFC1
	FTO			TTC9B
	FTSJ3			NHSL2
	FUBP1			ZNF296
	FUBP3			NSUN6
	FUCA1			PPT1
	FUNDC2			RPL24
	FURIN			MRPL30
	FUT1			CEBPA
	FXR1			MEG9

	FXR2			MRPL33
	FXYD6			PELO
	FYB1			PDE5A
	FYCO1			IL18R1
	FYN			MYO5C
	FYTTD1			STC1
	FZD4			AC079594.2
	FZD8			GULP1
	FZR1			LRP8
	G0S2			HIRA
	G2E3			ATXN2L
	G6PD			ZMPSTE24
	GABARAPL1			CD99L2
	GABARAPL2			H1-2
	GABBR1			SKA2
	GABPB1			RPL13A
	GABPB1-AS1			SNHG19
	GABRA5			ITM2C
	GABRB3			SIK3
	GABRE			PIH1D1
	GABRP			ZMIZ2
	GABRQ			MAN1A2
	GADD45A			FAM161A
	GADD45B			NPTX1
	GADD45G			PAK3
	GADD45GIP1			FKBP8
	GAK			SRSF6
	GAL			DHX37
	GALC			KDELR2
	GALNT1			POLK
	GALNT10			ADAMTS1
	GALNT11			UQCR10
	GALNT18			TPM2
	GALNT2			SRD5A3-AS1
	GALT			METTL7A
	GAN			COL18A1
	GAPDH			GATA3
	GAPVD1			TMEM47
	GARS-DT			RLIM
	GART			IFNA7
	GAS1			ABCC4
	GAS2L3			AC244517.11
	GAS5			NME1
	GAS6			TCEAL4
	GATA2			UBASH3A
	GATA6			HNRNPUL1
	GATD1			ZBTB17
	GATD3A			MED15
	GBA			GHITM
	GBA2			TSKU
	GBF1			NFIX
	GBGT1			NDUFB3
	GBP1			EIF3K
	GBP1P1			CLCN2
	GBP2			PDZK1IP1
	GBP3			SYS1
	GBP4			CMTM6
	GBP5			CDCA2
	GCA			ADAP1
	GCC1			USF3

	GCFC2			ID1
	GCH1			SLC16A3
	GCN1			MPZL1
	GCNT2			SPA17
	GCNT3			RDX
	GDE1			GSTM3
	GDF11			CAPN5
	GDF15			LST1
	GDI1			TBC1D16
	GDPD5			PLEKHG6
	GEM			SENP5
	GEMIN2			FOPNL
	GET3			CEP250
	GFER			TTC39B
	GFOD1			BTG2
	GFOD2			GSTP1
	GGA1			MRPL51
	GGA2			AJ003147.2
	GGA3			AC005332.6
	GGH			BISPR
	GGNBP2			AC105105.4
	GID8			ABCA5
	GIGYF1			KBTBD8
	GIMAP5			AC092117.1
	GIPC1			DTNA
	GIT1			NELFE
	GIT2			VANGL2
	GJA4			C4orf3
	GJA5			MTFR1
	GJC1			SVIP
	GK			VOPP1
	GK5			SELL
	GKN2			ERG28
	GLDN			EML4
	GLG1			SUDS3
	GLI3			DCAF13
	GLIPR1			BRF2
	GLMP			C11orf24
	GLOD4			CRY2
	GLRX			SSR1
	GLT8D1			ADK
	GMCL1			SEC62
	GMDS			TMED3
	GMEB2			RPL7A
	GMFG			NOD2
	GMPPA			IPO5
	GMPPB			OSGIN1
	GMPR			HMGN2P46
	GMPS			ESRP1
	GNA11			FAM214A
	GNA12			KIF2C
	GNAI3			RPA1
	GNAS			RNU11
	GNB1			FNDC3B
	GNB2			COX11
	GNB4			PPARGC1A
	GNB5			EIF2S3
	GNG11			MST1R
	GNG2			SRD5A3
	GNG5			STK17B

	GNG5P2			BAZ2A
	GNL1			PARP1
	GNL2			LSMEM1
	GNL3			AC073957.3
	GNL3L			CTSV
	GNLY			H1-6
	GNPAT			DCTN1
	GNPDA2			FAT1
	GNPNAT1			FSIP2
	GNPTG			ABCD1
	GNS			RNF130
	GOLGA1			FAM172A
	GOLGA2			SLC9A3R1
	GOLGA3			KLHDC7B
	GOLGA4			CHMP4B
	GOLGA6L4			MAPK13
	GOLGA6L5P			DENR
	GOLGA6L9			SLC7A1
	GOLGA7			CACNA1D
	GOLGA8A			NGRN
	GOLGA8B			F11R
	GOLGB1			CCDC71L
	GOLM1			KNOP1
	GOLPH3L			TMEM106C
	GON4L			TBK1
	GOT2			RAB4A
	GPAA1			PPFIA1
	GPANK1			BAAT
	GPAT4			SLC25A40
	GPATCH11			SAE1
	GPATCH2			AC011466.3
	GPATCH3			BTG1
	GPATCH4			RHBDD3
	GPATCH8			FLCN
	GPBP1			LPIN2
	GPBP1L1			IQCE
	GPC3			ROMO1
	GPD1			DIP2C
	GPD1L			APIP
	GPD2			ATP10D
	GPKOW			CERS6
	GPLD1			APRT
	GPM6B			AZIN2
	GPN1			TRIM36
	GPN2			HNF1B
	GPN3			HLA-DRB6
	GPNMB			AGPAT1
	GPR107			HSPE1
	GPR108			AL049766.1
	GPR132			BAIAP2L2
	GPR141			HINT1
	GPR155			BDNF
	GPR160			SATB2
	GPR161			TBCA
	GPR180			DOCK1
	GPR37L1			TERC
	GPR65			REC8
	GPR68			MXD4
	GPR84			EPHA2
	GPR89A			CAV2

	GPR89B			POLR1C
	GPRC5A			BTN2A3P
	GPRC5B			SMC1A
	GPS1			SBF2
	GPSM3			RAD54L
	GPX1			DHRS7
	GPX2			ZNF473
	GPX4			IL6-AS1
	GRAMD1A			IRGM
	GRASP			UBXN2B
	GRB10			HIP1
	GRB7			BMPR2
	GRHL1			AC007991.2
	GRHPR			KCTD3
	GRIN2D			PTGER2
	GRINA			AL360012.1
	GRK3			CUL7
	GRK5			MORF4L2
	GRPEL2			TMEM239
	GRSF1			IL18
	GRWD1			MAML3
	GS1-358P8.4			SEMA6A
	GSAP			RPS7
	GSDMD			PAGR1
	GSDME			CC2D1B
	GSK3A			INTS6L
	GSN			FOCAD
	GSN-AS1			PSRC1
	GSR			PSMD3
	GSS			CPNE3
	GSTA4			RNF216
	GSTM3			SNX12
	GSTO1			CHRDL1
	GSTP1			ATPSCKMT
	GSTT2B			EVPL
	GTDC1			RASD1
	GTF2B			CSAG3
	GTF2F1			AL031777.1
	GTF2H1			MAU2
	GTF2H2			TOMM40L
	GTF2H2B			ERO1B
	GTF2H2C			MICU1
	GTF2I			HMGN5
	GTF2IP1			GTF2F1
	GTF2IP13			SCAMP1-AS1
	GTF2IP4			TNFRSF12A
	GTF2IP7			TNFRSF11B
	GTF3C1			MAP3K10
	GTF3C3			ARL3
	GTF3C5			BTBD19
	GTPBP1			CYFIP1
	GTPBP10			HMGN1
	GTPBP2			OSTM1
	GTPBP4			MOB3B
	GTPBP6			MCM2
	GTPBP8			COX17
	GUCD1			NAA60
	GUCY1A2			SEMA4G
	GUF1			HPRT1
	GUK1			NHP2

	GUSB			RPL12	
	GYG1			WNT7B	
	GYPC			FZD5	
	GYS1			LINC02100	
	H1-0			TAOK3	
	H1-10			TTK	
	H19			DTX4	
	H2AC11			POC5	
	H2AC18			ZNF79	
	H2AC19			DLX2	
	H2AC20			RGS20	
	H2AC6			SMAD5	
	H2AC7			STIP1	
	H2AJ			AC006042.2	
	H2AX			MIS18BP1	
	H2BC12			STX3	
	H2BC18			SRP14	
	H2BC21			DNAJB5	
	H2BC4			MTCH2	
	H2BC5			SMAD2	
	H2BP1			AC104073.1	
	H2BU1			SLC25A1	
	H3-3A			PALM3	
	H3-3B			AC104532.1	
	H3-5			PKD2	
	H3C10			HID1	
	H3C4			NAPSB	
	H3P14			USP30-AS1	
	H3P16			PRDX1	
	H3P36			MTPN	
	H3P6			SRSF7	
	H4C14			NDUFA1	
	H4C15			ZBTB44	
	H4C9			COG3	
	HABP4			LAMB1	
	HACD2			NFKBIB	
	HACD3			APAF1	
	HACL1			WDR24	
	HAGH			WSB2	
	HAPLN3			PIK3C2B	
	HARS1			ACAT1	
	HARS2			ZNF75A	
	HAS2			NACC2	
	HAT1			NT5DC1	
	HAUS2			AC068860.1	
	HAVCR1			RN7SKP71	
	HBA1			SYCP2	
	HBA2			NDUFB5	
	HBEGF			IPO11	
	HBP1			MYO5A	
	HBS1L			MBTD1	
	HCAR1			HNRNPM	
	HCAR2			RRAD	
	HCFC1R1			ZMYND11	
	HCG18			CBX1	
	HCK			USP42	
	HCLS1			OGFRL1	
	HCST			WDR54	
	HDAC11			VDR	
	HDAC3			TXNDC12	

	HDAC4			SACS
	HDAC5			MTCO1P11
	HDAC6			AC022149.1
	HDAC7			C11orf68
	HDGF			AC005062.1
	HDHD2			KIAA0355
	HDHD5			HMGB1P6
	HDLBP			NDUFB1
	HEATR3			AC078802.2
	HECA			SLC2A4RG
	HECTD1			SMARCA2
	HECTD3			ATAD2B
	HECTD4			STYK1
	HELB			C1orf61
	HELLS			SPTBN2
	HELZ			TINAG
	HELZ2			ZNF672
	HERC1			MMP1
	HERC2			TASOR
	HERC2P2			TNFRSF19
	HERC2P9			SLC2A6
	HERC3			RYK
	HERC5			AC004130.2
	HERC6			GUK1
	HERPUD1			HS3ST1
	HES1			MYLIP
	HES2			GNL3L
	HES7			AF279873.2
	HESX1			LYRM7
	HEXA			FADS2
	HEXB			DRAM1
	HEXIM1			BMPR1B
	HEYL			WHAMM
	HGD			COTL1
	HGH1			ARRB2
	HGS			AL021707.3
	HHLA1			H2BC19P
	HHLA2			THAP12
	HID1			OS9
	HIF1A-AS1			LY75-CD302
	HIF1AN			AC011287.1
	HIGD2A			SRPK1
	HIKESHI			GPD1L
	HILPDA			AC110079.1
	HINFP			PRMT5
	HINT3			NECAP2
	HIP1R			CFI
	HIPK1			SNORA79B
	HIPK2			TRIQK
	HIPK3			PDZD8
	HIRA			NR2F2
	HIRIP3			SERPINH1
	HIST2H2BC			SAV1
	HK1			SART3
	HLA-A			CASC4
	HLA-B			PCCB
	HLA-DOB			PKM
	HLA-DPA1			FAF2
	HLA-DPB1			SNORD3D
	HLA-DQA1			SNORD3D

	HLA-DQB1			ADAM28
	HLA-DRB1			GPX8
	HLA-DRB5			UTP6
	HLA-H			NBPF10
	HLA-L			AC092153.1
	HLCS			HIGD1A
	HLTF			ATP4A
	HLX			VASP
	HM13			TECR
	HMBOX1			ZRSR2P1
	HMCES			ST6GAL1
	HMG20B			TCF3
	HMGB1P6			PCBP1-AS1
	HMGB2			UBOX5
	HMGCS1			C16orf58
	HMGN2			IFT27
	HMGN3			WDR1
	HMGXB3			CSE1L
	HMOX1			ACTR1B
	HMOX2			LMNA
	HNF1A			ARAP3
	HNF4G			KLHL20
	HNRNPA0			CXCL16
	HNRNPA1			PPP1R7
	HNRNPA1P48			SGSM2
	HNRNPA1P7			SPIN1
	HNRNPA2B1			PPM1H
	HNRNPA3P6			HNRNPH2
	HNRNPAB			DBF4B
	HNRNPC			INTS13
	HNRNPD			RELT
	HNRNPDL			DLD
	HNRNPH1			SNORA7B
	HNRNPH3			MYSM1
	HNRNPK			IFT57
	HNRNPL			VPS26B
	HNRNPLL			PSIP1
	HNRNPM			PGM2
	HNRNPR			C7orf31
	HNRNPUL1			NUF2
	HNRNPUL2			FER1L4
	HNRNPUL2-BSCL2			SH3RF1
	HOMER3			PLD1
	HOOK2			RPL30
	HOOK3			CNOT4
	HOPX			ESD
	HOTAIRM1			MAFB
	HOXA5			RPL24P4
	HOXC10			IMPAD1
	HOXC13			COX6B1
	HP			ZNF436
	HPCAL1			CYTH1
	HPF1			CDKN2A
	HPGDS			ZNF646
	HPR			CACNA1I
	HPRT1			MYH14
	HPS3			COL4A1
	HPS4			SLMAP
	HPSE			FSTL3
	HRAS			NRAS

	HS1BP3			PCF11-AS1
	HS2ST1			WDR25
	HS3ST3A1			RHOB
	HS6ST1			IP6K1
	HS6ST1P1			PISD
	HSBP1			CRIM1
	HSD17B10			CIB1
	HSD17B13			TMEM171
	HSD17B7			DCHS1
	HSD17B8			TSPAN3
	HSDL1			HSPG2
	HSF1			RPS15A
	HSH2D			TSTD1
	HSP90AB1			TLE4
	HSP90AB3P			CCT7
	HSPA12A			MED18
	HSPA12B			BUB3
	HSPA1B			RNF31
	HSPA2			RC3H1
	HSPA4			BCL9L
	HSPA4L			PITPNM1
	HSPA5			NUP155
	HSPA6			TMEM237
	HSPA7			AC009220.1
	HSPA8			PACSIN2
	HSPB6			ANKRD11
	HSPB7			RGL2
	HSPB8			EFHC1
	HSPD1			CLTC
	HSPG2			SLC6A9
	HSPH1			LINC01184
	HTATIP2			VKORC1
	HTATSF1			CCL3
	HTRA1			LGALS8
	HTRA2			SMOC1
	HTRA3			GNAI2
	HTT			NDUFB2
	HUS1			NARS1
	HYAL1			CPVL
	HYAL2			MGAT5
	HYI			KLF7
	HYKK			LRCH1
	HYOU1			SLC4A4
	IARS2			MYADM
	IBTK			AC087239.1
	ICA1			IGSF8
	ICAM1			MAGI1
	ICAM2			GALNT11
	ICE1			MRPS27
	ICOSLG			BRD4
	ID1			STAP2
	ID2			CTPS2
	ID3			NDUFA2
	ID4			PPA2
	IDH2			NSRP1
	IDH3A			NCAPG2
	IDO1			CLK3
	IER2			PLK2
	IER3			ARID1A
	IER5L			ASH1L

	IFFO1			NFKBIL1
	IFFO2			MED24
	IFI16			GCC1
	IFI27L1			BTAF1
	IFI27L2			C16orf70
	IFI44			ADD1
	IFI44L			MAP3K21
	IFI6			VWA2
	IFIH1			RINT1
	IFIT1			NDE1
	IFIT2			STAT4
	IFIT3			LINC02032
	IFIT5			CENPE
	IFITM1			GET1
	IFITM10			SLC4A7
	IFITM2			SAMM50
	IFITM3			ELL2
	IFNAR2			SERTAD2
	IFNGR1			POFUT1
	IFNGR2			TMCO3
	IFRD2			ZNF543
	IFT172			HNRNPA1
	IFT43			AC022966.1
	IFT46			AC011939.1
	IFT57			SZT2
	IGF1R			B3GALNT1
	IGF2BP1			PHTF2
	IGF2R			AC010168.2
	IGFBP1			NADK
	IGFBP2			CEP192
	IGFBP3			AC099343.4
	IGFBP6			ZNF707
	IGFL1			MMP7
	IGFL2-AS1			CRYZL2P
	IGFL4			NBPF14
	IGFLR1			ABCC9
	IGHMBP2			MUC4
	IGIP			MTMR12
	IGSF22			ALDH18A1
	IGSF3			NAALADL1
	IGSF6			APBA3
	IK			FBXO9
	IKBKB			AC009093.4
	IKBKGP1			AC132192.2
	IL10RB-DT			RWDD1
	IL11			N4BP3
	IL11RA			GK5
	IL12RB2			ZNF318
	IL13RA1			PKHD1L1
	IL15			MYRF
	IL15RA			TRAF7
	IL17C			BEX3
	IL18BP			CCT6P1
	IL18RAP			AC006458.1
	IL1A			USF2
	IL1B			CCDC85B
	IL1R1			PHLDB3
	IL1R2			MFSD1
	IL1RL1			HIVEP1
	IL1RN			DCAF7

	IL27RA			SMAD3
	IL2RG			RPL21
	IL3RA			AC116407.2
	IL4R			RSAD1
	IL6ST			LRRC45
	ILF3			ILVBL
	ILF3-DT			PFDN5
	ILKAP			USP53
	ILRUN			SRSF1
	ILVBL			CKLF
	IMMT			SIPA1L1
	IMP3			GADD45A
	IMP4			ZSCAN32
	IMPAD1			UVRAG
	IMPDH1			MAP1LC3B
	IMPDH2			PYGL
	INA			ELOB
	INAFM1			ARL1
	INF2			KALRN
	ING2			SLC8A2
	ING3			CLIC1
	ING4			NSD2
	ING5			NLN
	INHBB			RPL9
	INIP			LINC02328
	INKA2			TPI1P1
	INMT			PRTFDC1
	INO80			CLIP4
	INO80D			C2
	INO80E			NSA2
	INPP1			PRRC2A
	INPP4A			FAM162A
	INPP5A			TRAF2
	INPP5K			NSUN4
	INSIG1			IL6ST
	INSIG2			LRP1
	INSR			GALNT5
	INSYN2B			PLA1A
	INTS1			NEDD8-MDP1
	INTS10			EGLN3
	INTS11			ZNF324
	INTS12			SCARNA16
	INTS13			GCNT2
	INTS3			ADI1
	INTS5			SLCO3A1
	INTS6L			KDM3B
	INTS7			TMEM165
	IP6K1			KCNMB4
	IP6K2			IER5L
	IPO5			TXNRD1
	IPO8			ERBB4
	IQCA1			DPY19L4
	IQCB1			CLDN23
	IQCE			SNRPG
	IQGAP1			LIN54
	IQUB			NOP2
	IRAK3			CIC
	IRF1-AS1			SYNJ1
	IRF2			HSDL2
	IRF2BP1			PITX1

	IRF2BPL			SPATA6L
	IRF5			COX20
	IRF7			RNVU1-14
	IRF9			SAMD4B
	IRGQ			KANSL2
	IRS2			DEDD2
	IRX3			AC010967.1
	ISCA1			SPCS3
	ISCU			AL356234.1
	ISG15			TMEM170B
	ISG20L2			MGST2
	ISOC2			PHB
	IST1			LIMS1
	ISY1			AC009133.1
	ISYNA1			LTA
	ITCH			CACTIN
	ITGA10			ARPC3
	ITGA2			ZNF106
	ITGA3			BSG
	ITGA5			SOX9
	ITGA7			MYO3B
	ITGAE			KDM5B
	ITGAM			PPP1CC
	ITGAX			UNC45A
	ITGB1BP1			CCT5
	ITGB2-AS1			ZNF619
	ITGB3			LACTB2
	ITGB3BP			DOCK11
	ITGB4			HOOK3
	ITM2A			AGPAT5
	ITM2C			DNAJB9
	ITPA			ECHDC1
	ITPKB			PCYOX1L
	ITPKC			DDX6
	ITPR1			SYK
	ITPR3			MFF
	ITPRID2			KIDINS220
	ITPRIP			RALGAPA1
	ITSN1			CSF3
	IVD			IKZF2
	IVL			AL592437.2
	IVNS1ABP			TDP2
	IWS1			AC093510.1
	IYD			CHD6
	JADE1			BAK1
	JAG1			SLC2A12
	JAG2			FBXW2
	JAK2			THSD7A
	JAK3			AP000640.2
	JAM3			VEPH1
	JCAD			NDUFS5
	JMJD1C			SPOP
	JMJD1C-AS1			ZNF14
	JMJD6			DGKH
	JMY			TRMT112
	JOSD1			PLA2G6
	JOSD2			UGP2
	JPT1			NTN4
	JPX			AL353795.3
	JUN			S100A13

	JUNB			MIEN1
	JUND			PDE10A
	KALRN			GADD45G
	KANK2			CUEDC2
	KANK3			PPP1R18
	KANSL1			NRP1
	KANSL2			SETD7
	KANSL3			MIR1291
	KARS1			DCTPP1
	KAT2A			PMS2
	KAT5			SLC41A2
	KAT8			KLHL24
	KATNB1			GTF2IP13
	KATNBL1			CSRNP2
	KAZN			TTC19
	KBTBD11			MZT2B
	KBTBD2			SCNN1A
	KCMF1			SIRPB2
	KCNAB2			HMCES
	KCNAB3			STX11
	KCNC4			TCTN1
	KCND1			PLB1
	KCNE1			SLC16A5
	KCNE1B			UGT1A6
	KCNE4			SEH1L
	KCNJ2			HSD17B11
	KCNJ2-AS1			MATN2
	KCNK1			CDK18
	KCNK3			RPL35
	KCNK5			CDC42EP3
	KCNMA1			GMPS
	KCNQ3			ZFAND2A
	KCNS3			IER3IP1
	KCTD10			IQCK
	KCTD11			ADAMTS9
	KCTD12			SGK1
	KCTD15			NDRG2
	KCTD17			KDM1A
	KCTD18			PHF3
	KCTD2			MME
	KCTD20			CETN3
	KCTD3			DPP4
	KCTD5			WDHD1
	KCTD7			TMTC3
	KDELR1			LANCL1
	KDELR3			VWA1
	KDM1A			SPAG16
	KDM2A			EIF3A
	KDM3A			LINC00886
	KDM3B			DCP2
	KDM4C			ATP2B1
	KDM5C			SLC52A2
	KDM6B			ARL4D
	KDSR			UBA6
	KEAP1			ARMC1
	KHDRBS1			IL17RB
	KHDRBS3			TRIM52
	KHNYN			DENND4C
	KHSRP			NDUFA13
	KIAA0100			SUCLG2

	KIAA0355			ISG20L2
	KIAA0513			CDH1
	KIAA0556			MRPS35
	KIAA0895L			EYA1
	KIAA0930			MTMR3
	KIAA1217			CNIH4
	KIAA1324			PROX1
	KIAA1328			RBPJL
	KIAA1522			RPL3L
	KIAA1671			UBE2E3
	KIF14			RSPRY1
	KIF18B			IFNGR2
	KIF1C			YOD1
	KIF21B			CFAP36
	KIF22			MRFAP1
	KIF23			MDH2
	KIF3A			RPP38
	KIF3B			RBMS1
	KIF5B			HEBP2
	KIF9-AS1			ZNF184
	KIFBP			PIGN
	KIFC2			SUGT1P1
	KIFC3			TBCD
	KIRREL1			PTPN18
	KIZ			BAIAP2-DT
	KLC1			MTCH1
	KLC4			ARSB
	KLF10			GOLGA2P10
	KLF11			RBMX
	KLF12			STK26
	KLF13			AC002059.3
	KLF4			PRR15L
	KLF6			MATR3
	KLF7			VEZF1
	KLF9			IFNW1
	KLHDC2			KDM5C
	KLHDC3			H6PD
	KLHDC4			GTPBP4
	KLHDC7B			RAB6A
	KLHL21			ZNF618
	KLHL22			AC018521.1
	KLHL23			PPP2R1A
	KLHL24			RETREG2
	KLHL25			ZNF746
	KLHL3			TESC
	KLHL36			ALDH4A1
	KLHL42			HSPA1B
	KLHL9			DDX18
	KLK8			IMPACT
	KLRC2			PCK2
	KMT2A			SGPL1
	KMT2B			AGFG2
	KMT2E			AL671883.2
	KMT5A			AC020978.5
	KMT5B			AC021205.1
	KPNA2			LAMB2
	KPNA5			HOXB7
	KPNA6			ELOVL7
	KPNB1			YTHDC1
	KPRP			SHCBP1

	KRBOX4			DHX15
	KREMEN2			CNKSR3
	KRI1			TIMM23B-AGAP6
	KRIT1			LXN
	KRR1			PNPO
	KRT15			TMEM135
	KRT16P1			NDUFA6
	KRT16P2			RRBP1
	KRT17			TFDP2
	KRT17P6			AC020915.1
	KRT18			TXLNA
	KRT19			ADGRF1
	KRT23			MIGA1
	KRT31			LRIG3
	KRT42P			DPF2
	KRT81			YTHDF1
	KSR1			FLT3LG
	KTI12			MIR1248
	KXD1			ZFAND5
	KYNU			AMOT
	L1CAM			SLC35F5
	L2HGDH			IDS
	L3HYPDH			NDUFA5
	L3MBTL2			WDR34
	L3MBTL3			PRDX4
	LACC1			UBL5
	LAGE3			RNF25
	LAMA2			TBCB
	LAMA4			GRIPAP1
	LAMB1			AC007728.2
	LAMB2			PLOD3
	LAMC3			MATR3
	LAMP2			GAPDH
	LAMP3			PFDN4
	LAMTOR1			SLC39A6
	LAMTOR2			SNORA2C
	LAMTOR4			TRMT13
	LAMTOR5			CENPW
	LAMTOR5-AS1			CSF2
	LANCL3			ACSM3
	LAPTM4A			ARHGEF2
	LAPTM5			TSPAN15
	LARGE1			DNAJC8
	LARP1			RPL4
	LARP1B			NPC1
	LARP4			INCENP
	LARP4B			GOLPH3L
	LARP6			CXorf38
	LAS1L			ZNF408
	LASP1			PRDM1
	LAYN			LTA4H
	LBH			NACA
	LBR			AL021155.5
	LCAT			EIF2AK2
	LCE1C			GBP6
	LCN2			RAB27B
	LCP1			LIMCH1
	LCP2			PHF6
	LDB1			CDC27
	LDHA			MARK3

	LDHAP4			SESTD1
	LDHAP7			AC022916.2
	LDLR			PHGDH
	LDLRAD3			CNOT7
	LDLRAP1			BCLAF1
	LENG1			SLC31A2
	LENG8			S100P
	LEPROTL1			KLHDC2
	LERFS			EXOC3L1
	LETM1			TPRA1
	LFNG			PON1
	LGALS1			DDX3X
	LGALS3			TULP4
	LGALS3BP			OFD1
	LGALS9			TBC1D8B
	LGALS9B			OSTC
	LGALS9C			STAU2
	LGI4			IMPDH2
	LGMN			LINC01004
	LGR4			ZNF335
	LHFPL6			ZFHX2
	LIF			RFPL1
	LIFR			GNA13
	LILRA6			EGR4
	LILRB2			GUCY1B1
	LILRB3			RNU5F-1
	LILRB5			ARFGEF3
	LIMA1			CALM3
	LIN54			COX8A
	LINC00243			GPATCH4
	LINC00312			CEACAMP10
	LINC00342			RNU6-531P
	LINC00470			HROB
	LINC00504			ING3
	LINC00539			UQCRHL
	LINC00589			ALDH3B1
	LINC00605			EXOC5
	LINC00623			NEU4
	LINC00641			AC108134.2
	LINC00649			EHBP1L1
	LINC00665			TMEM268
	LINC00667			DDX56
	LINC00907			CDK19
	LINC00908			RHOBTB1
	LINC00923			RPL13AP5
	LINC00937			FNIP2
	LINC00958			ATXN10
	LINC01001			AKAP11
	LINC01002			E2F3
	LINC01126			TTBK2
	LINC01128			EPPK1
	LINC01136			RNF103-CHMP3
	LINC01138			RCAN3
	LINC01145			KCTD20
	LINC01234			AL645941.2
	LINC01270			UBR2
	LINC01278			EIF4E3
	LINC01303			PTPRJ
	LINC01347			PDGFC
	LINC01366			TM7SF3

	LINC01436			TSC22D1
	LINC01521			MED26
	LINC01550			AC115223.1
	LINC01559			TMOD3
	LINC01684			ENPP4
	LINC01695			DNAH3
	LINC01783			SNORA81
	LINC01806			CRK
	LINC01814			RNA5SP221
	LINC01833			MOCOS
	LINC01836			RNF141
	LINC01842			HAP1
	LINC01873			FAM120A
	LINC01979			LPA
	LINC02068			MCPH1-AS1
	LINC02352			CDK2AP1
	LINC02362			AP003498.3
	LINC02422			FOXC1
	LINC02535			AC025263.1
	LINC02541			ZSWIM6
	LINC02560			SPTBN5
	LINC02582			DDX1
	LINC02649			IVNS1ABP
	LINC02728			AZI2
	LINC02731			ATG2A
	LINC02762			FBXO7
	LINC02821			RPGRIP1L
	LINC02861			ATP2A2
	LINP1			SEL1L
	LINS1			RNVU1-7
	LIPA			IGHMBP2
	LITAF			TAF8
	LLGL2			AC107959.2
	LMAN2			PIF1
	LMAN2L			SNORA17B
	LMBR1			LINC00921
	LMBR1L			TWF1
	LMBRD1			ZNF850
	LMCD1			TMEM139
	LMNA			GPATCH2
	LMNB1			COX16
	LMNB2			TMEM265
	LMO1			CNTRL
	LMO2			AP3B2
	LMO7			H2AC21
	LMOD1			HKDC1
	LMTK2			B4GALT1
	LNPEP			SUMO1
	LNPK			KCNT2
	LNX2			DNAJC13
	LOH12CR2			ATP5PO
	LONP1			EDN1
	LONRF1			CRTAP
	LPAR6			PM20D2
	LPCAT1			E2F4
	LPCAT3			TLR5
	LPCAT4			RASSF8
	LPIN1			RASGRP1
	LPXN			SERPINB1
	LRATD2			BCYRN1

	LRCH1			GYS1
	LRCH3			ATXN7
	LRFN1			LINC00887
	LRFN3			SRSF5
	LRIG1			ALG14
	LRIG2-DT			AK2
	LRIG3			ARHGAP26-IT1
	LRP4			EEF2K
	LRP5			GNPTAB
	LRP5L			TIMM23B
	LRP6			SLC39A8
	LRPAP1			AC011195.1
	LRRC1			AC018638.4
	LRRC15			RN7SL5P
	LRRC23			BCL2L11
	LRRC27			PLAA
	LRRC32			VTI1B
	LRRC37A9P			TSLP
	LRRC37BP1			ASPHD2
	LRRC40			AC068305.2
	LRRC42			TMEM229B
	LRRC47			AL662844.4
	LRRC59			ZWINT
	LRRC8A			FAM219A
	LRRC8E			AC020915.5
	LRRFIP1			IVD
	LRRFIP2			RHNO1
	LRRK2			HAND1
	LRRK2-DT			H2AC4
	LRSAM1			FGF2
	LSG1			MYL6B
	LSM1			AC245033.4
	LSM10			USP22
	LSM12			ATP5MC3
	LSM14A			SPTLC1
	LSM3			KIAA0586
	LSM4			INTS12
	LSM7			SLC35B2
	LSS			SLC50A1
	LST1			ABCG1
	LTA4H			ZNF285
	LTB4R			DDHD2
	LTBP1			RARS1
	LTBP2			SSPN
	LTBP3			MRPL45
	LTF			PPP4C
	LTO1			ZADH2
	LUARIS			CEBPZOS
	LUC7L			ALDH6A1
	LUC7L2			FAM171B
	LUC7L3			MAGED2
	LURAP1L			MARCHF5
	LUZP1			GINS2
	LY6E			PDCD1LG2
	LY6G5B			AL078599.3
	LY6G5C			RP1
	LY75			PI4KB
	LY96			AC244197.3
	LYAR			PRPF31
	LYN			AC119674.1

	LYPLA1			EXOC8
	LYPLA2			C21orf91
	LYRM1			HCG4
	LYSMD2			RNF20
	LZTR1			Z93930.2
	LZTS1			GOLM1
	LZTS2			PTGFRN
	LZTS3			TSPAN12
	MACF1			LRRC8E
	MACO1			GGT1
	MAD2L2			LYRM2
	MADD			SNORA17B
	MAF1			PIM3
	MAFF			AXL
	MAFG			SNX17
	MAFK			RERG
	MAGEF1			ATP7A
	MAIP1			GPN3
	MAL			ABCA2
	MALL			ARL4C
	MALSU1			VGLL3
	MAMDC2			ACTR1A
	MAML1			CYBRD1
	MAMLD1			SUGT1
	MAN1B1			CTSA
	MAN2A1			SKP2
	MAN2A2			SERINC3
	MAN2C1			CERCAM
	MANBA			HJURP
	MAOA			POLA1
	MAP1LC3B			MRPL19
	MAP1LC3B2			ADAM22
	MAP1S			SLC41A1
	MAP2K2			ADM2
	MAP2K3			RNFT2
	MAP2K5			FYTTD1P1
	MAP2K6			MICAL2
	MAP2K7			CAB39
	MAP3K12			PLEKHA3
	MAP3K13			SHC3
	MAP3K14			MUC13
	MAP3K20			PDE4B
	MAP3K2-DT			NAA50
	MAP3K3			HMG20B
	MAP3K6			COL22A1
	MAP3K7CL			TANC2
	MAP4			TBILA
	MAP4K5			CYP27A1
	MAP7D1			KLF2
	MAP7D2			TIMMDC1
	MAPK1			VCPIP1
	MAPK11			FBXW10
	MAPK12			COA4
	MAPK14			MYO18A
	MAPK3			TTC37
	MAPK7			DCPS
	MAPK8			INF2
	MAPK9			CHDH
	MAPKAP1			TMEM134
	MAPKAPK2			AL354740.1

	MAPKAPK3			SLC44A3
	MAPKAPK5			AC090541.1
	MAPKBP1			ERGIC2
	MAPRE1			GAREM1
	MAPRE3-AS1			CDK16
	MARCHF1			KMT2A
	MARCHF2			UQCRC1
	MARCHF4			TYRO3
	MARCHF5			USP34
	MARCHF7			AEBP2
	MARCKS			IGHEP2
	MARF1			MRPL11
	MARK3			LRRC8A
	MARK4			CYP4F3
	MARS1			TES
	MAST1			SCAF1
	MAST2			BET1L
	MAT2A			ECI2
	MAU2			APP
	MAX			PAFAH1B2
	MBD1			AMIGO2
	MBD2			SUMO2
	MBD3			RBIS
	MBIP			THSD1
	MBNL1			HCCS
	MBNL3			AC005622.1
	MBOAT7			LNPEP
	MBP			AMZ2
	MBTD1			BX276092.9
	MBTPS2			TFCP2L1
	MCAM			MIR210HG
	MCAT			PPIA
	MCC			LCAL1
	MCCC1			PRMT2
	MCCC2			EGR3
	MCF2L			CNIH1
	MCFD2			DPH5
	MCIDAS			OPRK1
	MCL1			PROS1
	MCM3AP			AL022311.1
	MCM7			NME1-NME2
	MCOLN1			AC125807.2
	MCOLN2			CHPT1
	MCRIP1			BTG3
	MCRIP2			CYTH3
	MCTP2			ISOC1
	MCTS1			P3H2
	MDC1			CCDC115
	MDFIC			N4BP2L2-IT2
	MDH1			RBM22
	MDH1B			GNL1
	MDH2			TEPSIN
	MDK			LSM7
	MDM1			NUP160
	MDM2			POLR3A
	MDN1			MYRFL
	ME2			CCDC86
	ME3			BCL7C
	MECP2			AC020907.3
	MED1			AC005840.4

	MED10			TTC30A
	MED11			AC092868.2
	MED12			PAFAH1B3
	MED13L			FAHD1
	MED15			AC097059.1
	MED16			MSANTD4
	MED17			AC083880.1
	MED18			AC084018.2
	MED20			VPS29
	MED22			GPC3
	MED23			USP49
	MED24			PPP4R1L
	MED27			CEACAM20
	MED4			HYOU1
	MED6			SP8
	MED8			BIN1
	MED9			CCND1
	MEF2D			ARL14EP
	MEG3			TXNDC11
	MEIS1			SRPRA
	MEIS2			DNAH11
	MEPCE			LINC00431
	MERTK			ZNF638
	MESD			ZNF654
	METRNL			PHYH
	METTL16			LIMA1
	METTL17			AL671277.1
	METTL23			MECOM
	METTL26			ANXA13
	METTL2A			GPR161
	METTL3			KLHDC3
	METTL5			CARNMT1
	METTL7A			UBXN4
	METTL7B			EPHB2
	METTL9			PLEKHG5
	MFAP1			LINC00881
	MFGE8			RPP25
	MFHAS1			REXO1
	MFN2			AL031587.5
	MFNG			FN3KRP
	MFSD1			CCDC171
	MFSD10			GCNA
	MFSD14A			LAMP2
	MFSD2A			AL450384.2
	MFSD5			SOAT1
	MFSD6			MIS12
	MGA			SNX16
	MGAM			HECTD1
	MGAT1			NLRP3
	MGAT4B			RHOF
	MGAT4C			C5orf24
	MGAT5			ALS2
	MGLL			NDUFC2
	MGMT			PRXL2B
	MGRN1			CAPN1
	MGST3			FREM2
	MIA2			RNU6-9
	MIA3			PVT1
	MIB1			ACTR2
	MICA			ZKSCAN2

	MICAL1			ZNF702P
	MICAL3			DNHD1
	MICALL2			MTG2
	MICB			GMEB2
	MICOS10			SUSD3
	MICOS13			AP001160.1
	MID1			CCDC130
	MID1IP1			SMARCD2
	MID2			MIER1
	MIDN			PET100
	MIEF1			ADAMTSL4-AS1
	MIEN1			KRT18
	MIER1			DYNLT1
	MIER2			AC009495.3
	MIF4GD			WASF2
	MIGA2			EPB41L2
	MILR1			FAH
	MINDY3			AC126283.2
	MINK1			AIG1
	MIOS			TPMT
	MIPOL1			CRLF2
	MIR22HG			AHCYL1
	MIR29B2CHG			CAPZA2
	MIR3189			CCDC191
	MIR3945HG			YWHAB
	MIR4697HG			MPI
	MISP			MTDH
	MKI67			STX5
	MKNK2			ADGRF4
	MKRN2			AC024293.1
	MLEC			TXNDC17
	MLF2			RHEBL1
	MLH1			RPL17-C18orf32
	MLH3			TCN2
	MLKL			AC025539.1
	MLLT1			GRK6
	MLLT10			CRYBG2
	MLLT11			ZMAT3
	MLNR			STAT6
	MLST8			ATP6V1C1
	MLX			SH3BGRL
	MLXIP			PIK3IP1
	MLXIPL			PLAGL2
	MMAA			SNX33
	MMAB			PHLDB2
	MMADHC			SCARNA22
	MMP1			OIP5
	MMP10			AP003499.4
	MMP12			TMEM44-AS1
	MMP14			NAA38
	MMP24OS			TOP3B
	MMP25			ZKSCAN8
	MMP25-AS1			PBDC1
	MMP28			WDFY2
	MMP3			BMP2K
	MMS19			ODF2L
	MNAT1			CSF1R
	MND1			ABCA6
	MOAP1			RB1CC1
	MOB1A			GPD2

	MOB1B			FBXL12
	MOB2			SLC43A2
	MOB3C			RAB40B
	MOCOS			ACNATP
	MOCS1			TET3
	MOGS			PPP2R2A
	MON1A			AKTIP
	MON1B			KRCC1
	MON2			LRIF1
	MORC2			XDH
	MORC3			MROH8
	MORC4			FAM225B
	MOSMO			LOX
	MOSPD1			MRPL47
	MOSPD2			NFATC4
	MOV10			ZNF652
	MPDU1			PCYT2
	MPG			FBXO17
	MPHOSPH10			CDK5RAP3
	MPHOSPH8			IGF1R
	MPLKIP			AKAP10
	MPP1			TAF4B
	MPP3			UBTF
	MPP4			NUP58
	MPPE1			HMCN1
	MPRIP			POU2F2
	MPST			VPS28
	MPV17			ZFP62
	MPZ			NDUFAB1
	MPZL1			HIBADH
	MPZL2			MALAT1
	MR1			ACAD10
	MRE11			MGLL
	MRFAP1			NFKBID
	MRFAP1L1			SIAH2
	MRGBP			AL450338.1
	MRI1			Z95114.1
	MRM2			GNS
	MRM3			ABCA10
	MRNIP			PARP4
	MRO			SH3TC2
	MROH1			ZNF597
	MROH8			TRDMT1
	MRPL1			DMKN
	MRPL10			TMEM205
	MRPL11			HEXB
	MRPL14			MIR3143
	MRPL17			SPAG1
	MRPL18			FGFR2
	MRPL2			RPE
	MRPL20			OASL2P
	MRPL22			MKKS
	MRPL23			PDCD4
	MRPL28			YBX1P10
	MRPL3			NABP2
	MRPL30			TNKS2
	MRPL32			CEP97
	MRPL33			FITM2
	MRPL34			PLXNA3
	MRPL37			RPL36A-HNRNPH2

	MRPL39			MRTFA
	MRPL4			RANGAP1
	MRPL40			BIRC2
	MRPL41			PBX2
	MRPL44			NSD3
	MRPL48			AC009779.4
	MRPL49			ALDOA
	MRPL51			KCTD5
	MRPL52			SCO2
	MRPL54			REXO2
	MRPL55			ATP5ME
	MRPS10			NUDT16
	MRPS15			HSD17B2
	MRPS18A			PRCP
	MRPS2			ARSL
	MRPS21			ZNF764
	MRPS26			SEC16A
	MRPS33			AC108010.1
	MRPS34			RIOK3
	MRPS5			TMBIM1
	MRPS7			UNK
	MRS2			BZW2
	MRTFA			ZFPM2-AS1
	MRTFB			PTPRK
	MSANTD3			LINC01554
	MSANTD4			AC004477.1
	MSC			ZNF711
	MSH5			ZNF888
	MSH6			NFIB
	MSL3			STK40
	MSMO1			SLPI
	MSR1			C2orf42
	MSRB2			IL1B
	MSTO1			UHRF1
	MSX2			SLC16A6
	MT1XP1			BRAF
	MT2A			APMAP
	MT2P1			RPS29
	MTA2			CA12
	MTA3			GDF11
	MTCH1			OGT
	MTCH2			OAT
	MT-CO1			SLC25A39
	MTCO1P12			P4HA1
	MT-CO2			MIR7-3HG
	MT-CO3			ALX1
	MT-CYB			AL445685.3
	MTDH			ADGRL2
	MTERF2			AC005747.1
	MTF1			ZNF891
	MTFR1L			WDR72
	MTHFD1			CKS2
	MTHFD1L			ERBIN
	MTHFD2			RPL10A
	MTHFD2L			AGBL5
	MTHFR			SLC39A9
	MTHFSD			BRCC3
	MTIF3			AC051619.7
	MTLN			SLC9A6
	MTM1			C4BPB

	MTMR10			POLA2
	MTMR12			FAM71A
	MTMR14			DHFRP1
	MTMR2			AC009133.6
	MTMR9LP			LINP1
	MT-ND1			NT5C3AP1
	MTND2P28			SCO2
	MT-ND6			KLHL23
	MTOR			PSMA5
	MTR			CA8
	MTREX			CHD9
	MTRF1L			EPHA7
	MTRNR2L12			ALDH2
	MTRNR2L8			CASK
	MTSS2			TTC17
	MT-TC			SLC19A3
	MT-TP			OXTR
	MTURN			DPCD
	MTX1			CYB561
	MTX1P1			EPHX1
	MTX3			RPN2
	MUC1			RPP14
	MUC20-OT1			UBR4
	MUC4			POLD4
	MUC5AC			AC099518.3
	MUC5B			ATP6V1G2-DDX39B
	MUL1			AC008750.8
	MVB12B			FBL
	MVD			LINC01612
	MVP			PGAM1P7
	MX1			OLFM3
	MX2			NUDCD1
	MXD1			KIAA1143
	MXD4			IPMK
	MXI1			CTDSP2
	MXRA5			SEC22C
	MXRA7			ARHGAP19
	MXRA8			ARID5A
	MYADM			PPP3CA
	MYBBP1A			RTF2
	MYC			TBC1D15
	MYCBP2			DFFA
	MYCT1			ALG8
	MYD88			CLNS1A
	MYDGF			TNFRSF21
	MYH14			FBXO22
	MYL12A			AC243772.2
	MYL12B			GPBAR1
	MYL6			MYO19
	MYL6B			LPCAT2
	MYLK			LINC01358
	MYO10			G3BP1
	MYO15B			GID8
	MYO18A			AC005154.5
	MYO1C			GATM
	MYO1D			SGPP1
	MYO1G			PLS3-AS1
	MYO6			TMPRSS3
	MYO9A			SCAF4
	MYO9B			CLU

	MYOF			NCK1
	MYOM2			SH3RF3
	MYZAP			SRP72
	MZB1			MUC20
	MZT2A			SPCS1
	MZT2B			ZNF212
	N4BP1			POLG
	N4BP3			SNHG17
	NAA10			C6orf62
	NAA20			TMC7
	NAA35			PDXDC1
	NAA38			SAP30BP
	NAA60			ZCRB1
	NAB1			CCNB1IP1
	NAB2			PPP1R9A
	NACA			NFRKB
	NADK			EIF2AK1
	NADK2			NME4
	NADSYN1			CA11
	NAGK			DENND11
	NAIF1			USP19
	NAMPT			C22orf39
	NAMPTP1			ATP6V0E1
	NANP			RAF1
	NAP1L4			LINC00992
	NAP1L5			BTD
	NAPA			NUDT2
	NAPG			RHOC
	NAPSA			AL023755.1
	NARF			ASS1P11
	NARS1			AGBL1-AS1
	NASP			CMYA5
	NAT10			AC073869.1
	NAT14			MRPL42
	NAT9			TMEM168
	NATD1			AC007923.1
	NAXD			CETN2
	NAXE			TMEM129
	NBAS			AC104837.2
	NBL1			OSR2
	NBN			AC006960.4
	NBPF11			HTD2
	NBPF14			IDH3B
	NBPF15			HABP4
	NBPF19			TLN2
	NBPF20			RNVU1-18
	NBPF25P			PRPF38B
	NBPF26			RPL37A
	NBPF8			LRPAP1
	NBPF9			OR4V1P
	NBR1			SLC25A24
	NCALD			DSG2
	NCAPH2			CACNG4
	NCBP2AS2			FRMD4B
	NCBP3			PACERR
	NCCRP1			THYN1
	NCDN			GTF3A
	NCEH1			USP47
	NCF2			KIFC2
	NCF4			AC091167.2

	NCKIPSD			BDH1
	NCL			ALDH5A1
	NCOA3			OLA1
	NCOA4			AC245041.2
	NCOA7			SUCLA2
	NCOR2			VSIG1
	NCS1			CHKB-CPT1B
	NCSTN			MYO1D
	NDEL1			LINC-PINT
	NDN			PTPRG-AS1
	NDNF			TRIM67
	NDRG1			DUSP15
	NDRG2			AL451165.1
	NDRG3			AL603832.3
	NDST1			TMEM64
	NDUFA10			STAC
	NDUFA12			ANKRD28
	NDUFA4			INTS1
	NDUFA4L2			CAPRIN2
	NDUFA5			DDX55
	NDUFAB1			ACTR3B
	NDUFAF2			PRPF38A
	NDUFAF5			LTB
	NDUFAF8			ARF5
	NDUFB1			PGM1
	NDUFB10			SELENOT
	NDUFB3			SNORD12B
	NDUFB4			GRB14
	NDUFB4P12			ARMC5
	NDUFB8			TMEM213
	NDUFB9			MGAT1
	NDUFC2			ERCC6L2
	NDUFS2			DBR1
	NDUFS4			PAX8-AS1
	NDUFS5			RNVU1-19
	NDUFS6			TK1
	NDUFS7			MRPL44
	NDUFS8			MIR222HG
	NDUFV1			AC079466.1
	NDUFV2-AS1			MYBL2
	NEAT1			FBRS
	NEBL			USP10
	NECAP1			JADE1
	NECAP2			AL136164.3
	NECTIN2			ARHGAP1
	NEDD1			GLB1
	NEDD4L			H2BC8
	NEDD8			PARP6
	NEDD9			RRP9
	NEIL2			C17orf75
	NEK3			PEPD
	NEK5			DDC
	NEK7			ZNF382
	NEK9			NSUN5
	NELFA			RAD9A
	NELFB			PDLIM1
	NELFCD			WNT2B
	NELFE			ALAD
	NEMP2			PCLAF
	NENF			RAB13

	NES			FRMD3
	NEURL1B			TACSTD2
	NEXN			BRK1
	NF2			PCMTD2
	NFASC			ZMYND8
	NFATC1			ECHDC2
	NFATC2			ZFP69B
	NFATC4			GANC
	NFE2L1			AIFM1
	NFE2L3			KLHL18
	NFIB			AP005482.2
	NFIL3			TBC1D32
	NFIX			LAMTOR4
	NFKBID			MDFIC
	NFKBIL1			NCOA5
	NFRKB			RPL11P3
	NFX1			RNF187
	NFYC			PAQR4
	NGRN			CCNDBP1
	NHLRC2			HOXB3
	NHP2			SNORA20
	NIBAN1			YBX1P1
	NIBAN2			CAVIN1
	NID1			MEF2D
	NIF3L1			FKBP3
	NIN			AGTPBP1
	NINJ1			ATRN
	NIPA2			SLC10A7
	NIPAL3			NIPBL
	NIPAL4			SERPINB8
	NIPBL			MMP24OS
	NISCH			FBXO38
	NIT1			USP9X
	NKD1			ZNF280C
	NKIRAS2			ZNF592
	NKTR			GINS4
	NLE1			GATD1
	NLN			YDJC
	NLRP1			PPP1R13L
	NLRP3			HSPB11
	NMD3			ZSWIM3
	NME1			CLEC4O
	NME3			MYO1E
	NME5			MRPL43
	NME8			TPD52
	NMI			SERPINB5
	NMRAL1			CHMP2A
	NMRAL2P			TFAM
	NMT1			EIF3I
	NMT2			SNHG8
	NMU			MAGED1
	NNT			COMMD4
	NOC2L			TOPBP1
	NOC3L			SLC38A6
	NOC4L			JDP2
	NOD1			DDR1
	NOL6			CGAS
	NOL8			TMED9
	NOLC1			ARFRP1
	NOMO3			FOXN3

	NOP10			SLCO5A1
	NOP16			AP000331.1
	NOP2			CDH18
	NOP53			SMG1
	NOP56			GYG2
	NOP58			C1orf21
	NOP9			ITGB1BP1
	NORAD			STOML2
	NOSIP			IMPDH1P10
	NOSTRIN			STYX
	NOTCH1			POU2F1
	NOTCH2			AC141586.1
	NOTCH4			PLPBP
	NOVA2			KHDRBS1
	NOXRED1			DEK
	NPAS1			FUT10
	NPC1			GALC
	NPC2			AMFR
	NPHS1			HIGD2A
	NPIPA1			TCEA1
	NPIPA3			POU5F1
	NPIPB11			C11orf54
	NPIPB12			H2BC5
	NPIPB13			LCN2
	NPIPB3			HSPA8
	NPIPB4			C1orf52
	NPIPB5			TMEM141
	NPIPP1			AP000944.5
	NPL			RGS2
	NPLOC4			NCS1
	NPM1			BRD1
	NPR1			HIBCH
	NPR2			MYNN
	NPR3			IGFBP2
	NPRL2			E2F1
	NQO1			ZC3H4
	NQO2			NDUFA10
	NR1D2			LPAR1
	NR1H2			TNFRSF14
	NR1H3			AC112907.3
	NR2C2			AC007255.1
	NR2C2AP			TXNL4A
	NR2F1-AS1			DYNC1I2
	NR4A1			SERTAD3
	NR4A2			PON2
	NR5A2			MAFK
	NRBP1			SAYSD1
	NRCAM			BBS4
	NRDC			SELENOH
	NRF1			CHCHD1
	NRGN			TSNARE1
	NRIP1			DCBLD2
	NSD2			UTP18
	NSF			MCM5
	NSMAF			IL1R1
	NSMCE1			PRELID3B
	NSMCE4A			SDE2
	NSMF			TIMM23
	NSRP1			EDEM1
	NSUN2			RPL27

	NSUN4			MYCL
	NSUN5			C2CD5
	NSUN5P1			MMP24
	NSUN7			CSTF3
	NT5C			TMEM217
	NT5C3A			LINC00842
	NT5C3AP1			TDRKH
	NT5C3B			ANKRD18B
	NT5DC2			CCDC25
	NTHL1			GLT8D1
	NTMT1			FCF1
	NTNG2			STK25
	NUAK1			SCAMP1
	NUB1			GSDMC
	NUBP1			CERS2
	NUCKS1			DNAH12
	NUDC			EPB41L4A-AS1
	NUDCD3			AC245041.1
	NUDT12			HIST1H3B
	NUDT16L1			CDC42P6
	NUDT4			XPO7
	NUMA1			HPS5
	NUMB			SNORA7A
	NUP133			MCM3AP
	NUP153			TNPO1
	NUP155			MCM3AP-AS1
	NUP188			LATS1
	NUP43			CKLF-CMTM1
	NUP62CL			MEFV
	NUP85			UGCG
	NUPR1			ALKBH1
	NUS1			HELQ
	NUTF2			ENO3
	NVL			ZNF35
	NXF1			MPRIP
	NXNL2			RPL17
	NXT1			H4C13
	NXT2			PTK7
	NYNRIN			CTSL3P
	OAF			KDM4A
	OARD1			PPP1R14B
	OAS1			COMMD7
	OAS2			TAF2
	OAS3			STRIP1
	OASL			DMD
	OAT			PANX1
	OAZ2			RAP1A
	OBSCN			TTLL1
	ODC1			MRPL23
	ODF2			ATM
	ODF2L			SCRN3
	ODF3B			SNORA21B
	OGA			HNRNPK
	OGDH			CCDC152
	OGFOD1			FUNDC2
	OGG1			ACBD5
	OLA1			AF196969.1
	OLIG1			SETDB1
	OLR1			MED8
	OMA1			ECD

	OPHN1			TPT1
	OR7E14P			FYN
	OR7E36P			TMEM72-AS1
	OR7E37P			AL365203.2
	OR7E38P			AKAP2
	ORAI1			RNPEPL1
	ORAI2			RAB42
	ORC2			AC091271.1
	ORC3			MZF1-AS1
	ORMDL3			RO60
	OS9			MARS2
	OSBPL10			SLC17A9
	OSBPL1A			CXCR4
	OSBPL2			NKIRAS2
	OSBPL5			MYBBP1A
	OSBPL7			CPNE8
	OSBPL9			ANO3
	OSER1			ID2
	OSGEP			TOGARAM1
	OSGIN2			BRCA1
	OTUB1			TNFRSF8
	OTUD3			CCNF
	OTUD4			CCR4
	OTUD7B			OR1F2P
	OTULINL			CLEC7A
	OTX1			ZFAND6
	OXA1L			PUS7
	OXCT1			LNX1
	OXR1			ATP5MC2
	OXSR1			TBRG4
	P2RX4			YIF1B
	P3H1			RPL6P27
	P3H2			DHX38
	P3H3			RPL7P1
	P3H4			ADCY7
	P4HA2			GLOD4
	P4HB			SNX27
	PA2G4			SCGN
	PAAF1			ROGDI
	PABPC1			FAM199X
	PACC1			NELFB
	PACS2			MTR
	PACSIN2			SCIN
	PACSIN3			CHRNA5
	PADI2			POLR2L
	PAF1			METTL5
	PAFAH1B2			ZNF395
	PAIP1			KIF3B
	PAK1			STXBP6
	PAK2			CYP21A1P
	PALD1			HSPA14
	PALLD			TGFBR1
	PALM			UGT8
	PALMD			METRN
	PAM			GTF2A2
	PAMR1			EWSR1
	PAN2			ZDHHC14
	PAN3			VSIG10
	PANK3			ZBTB40
	PANK4			MALSU1

	PAPOLA			PPIL4
	PAPPA			GSTK1
	PAPSS2			RAB1A
	PAQR6			YIF1A
	PAQR9			IFT172
	PARD3			AGGF1
	PARG			FBLN1
	PARK7			TMEM14B
	PARL			VIM-AS1
	PARN			ERP29
	PARP11			TMCO1
	PARP12			STK3
	PARP14			ADAM20
	PARP15			LINC02827
	PARP16			NCDN
	PARP2			SPCS2
	PARP3			PCED1A
	PARP4			BMI1
	PARP4P2			WDFY3
	PARP8			METTL1
	PARP9			AC022106.1
	PART1			YARS1
	PARVA			AC010619.3
	PARVB			NGDN
	PARVG			LINC02842
	PASK			ABCC10
	PATL1			BORCS8
	PATL2			SLC35A5
	PAX8-AS1			PRR34-AS1
	PAX9			NRBF2
	PAXBP1			CDH3
	PAXBP1-AS1			SLC35B1
	PAXX			PLCB1
	PBRM1			NPHP1
	PBX1			NABP1
	PBX2			SNHG22
	PBXIP1			CEP68
	PCBD1			AC092117.2
	PCBP1			GMNN
	PCBP1-AS1			TBX18
	PCBP2			NRIP1
	PCBP4			PITPNB
	PCCA			BCR
	PCCB			GFPT2
	PCDH1			POF1B
	PCDH12			H2BC13
	PCDHA11			SNUPN
	PCDHGB5			KLHL36
	PCED1A			NUCB1
	PCED1B			WWP1
	PCED1B-AS1			RASA1
	PCGF2			MTX3
	PCGF3			BLACAT1
	PCGF5			CD302
	PCID2			LAMTOR2
	PCMT1			ZNF316
	PCNX1			LRPPRC
	PCNX2			PCDHAC2
	PCNX3			PRPF6
	PCP4L1			URB1

	PCSK9			MED13L
	PCTP			UCHL5
	PCYOX1			DOP1A
	PCYT1A			GDE1
	PCYT1B			COL12A1
	PDCD11			MAP3K4
	PDCD1LG2			COMMD6
	PDCD2			CRY1
	PDCD4			MPP7
	PDCD4-AS1			ATXN7L1
	PDCD5			NDUFS6
	PDCD6			SPDL1
	PDCL			CLSPN
	PDCL3			SH3RF2
	PDE11A			TCF19
	PDE1C			ATP7B
	PDE2A			PAXBP1-AS1
	PDE4B			PHF19
	PDE4DIP			AL353719.1
	PDE5A			RABGGTA
	PDE6D			STX1B
	PDE8B			MAPKAP1
	PDE9A			PAFAH2
	PDGFB			COPZ1
	PDGFC			NDOR1
	PDGFRA			TUBB2A
	PDGFRB			ACSL5
	PDGFRL			ATP5MF-PTCD1
	PDIA4			XPO1
	PDIA5			KHSRP
	PDIA6			RBM23
	PDK4			POC1B-GALNT4
	PDLIM2			DDX28
	PDLIM3			GTF2E2
	PDLIM4			BNIP5
	PDLIM5			PCMTD1
	PDPK1			RALGAPA1P1
	PDPN			TEFM
	PDRG1			TBX3
	PDS5A			NAB1
	PDSS1			RPN1
	PDXDC1			AC073109.1
	PDXK			CAPN15
	PDZD11			H2AC15
	PDZD8			ABI2
	PEAK1			ZDHHC20
	PEAR1			PPRC1
	PEBP1			C3orf38
	PELI1			RBMXL1
	PELO			DYNLL1
	PELP1			AL732366.1
	PEPD			LMBR1L
	PER1			CHST4
	PER2			AC005090.1
	PER3			LIMK2
	PES1			SIRPA
	PEX1			MSL3
	PEX11B			ATP6V0E2
	PEX16			MMP25-AS1
	PEX2			PPM1A

	PEX5			AC092279.1
	PFAS			BCKDHB
	PFDN1			FECH
	PFDN2			PPAN
	PFDN5			LAGE3
	PFDN6			MANEA
	PFKFB4			KCNK5
	PFKL			COX6A1
	PFKM			TKFC
	PFKP			ARFIP1
	PFN1			AC006064.6
	PGAM4			SLC2A13
	PGAP3			THAP6
	PGAP4			NUDT5
	PGAP6			OSBPL1A
	PGBD1			MARK2
	PGBD4			AL645608.8
	PGC			RNASEL
	PGD			ACSS2
	PGGHG			AC135050.2
	PGLYRP1			POLR2A
	PGM5			GSK3A
	PHB			CASP3
	PHB2			BCCIP
	PHC1P1			CNPPD1
	PHC2			ARHGEF11
	PHETA2			NHLRC2
	PHF10			LINC01588
	PHF11			ETV1
	PHF12			RAB2A
	PHF13			GLRX5
	PHF20			ATXN7
	PHF20L1			MIRLET7A1HG
	PHF23			RPS27A
	PHF5A			AC022400.3
	PHF6			BOC
	PHKB			AC074050.2
	PHKG2			AKAP7
	PHLDA1			COA5
	PHLDA2			PTPRZ1
	PHLDA3			ATP5F1B
	PHLDB1			AC093425.1
	PHLPP1			THBS3
	PHLPP2			FOXK1
	PHPT1			CXCL6
	PHRF1			SH3GLB2
	PHTF2			QSOX1
	PI4K2A			TROAP
	PI4K2B			KRBA1
	PI4KA			TERF2IP
	PI4KAP1			AC009951.4
	PI4KAP2			TRIM29
	PI4KB			AC008074.2
	PIAS2			DIAPH1
	PIAS3			GALNT4
	PIEZO1			RBAK
	PIGC			RNU5D-1
	PIGL			AC093512.2
	PIGO			RFFL
	PIGR			ELP2

	PIGS			SEPSECS
	PIGU			OTUB1
	PIGV			MEA1
	PIH1D1			RYBP
	PIK3AP1			UBA1
	PIK3C2B			TYSND1
	PIK3IP1			FAM217B
	PIK3R1			PHRF1
	PIK3R4			RPA3
	PILRA			HEATR1
	PIM1			CNKSR1
	PIM2			SLC66A3
	PIN4			DCTN2
	PINX1			MRPL18
	PIP			CCDC186
	PIP4P1			PKIB
	PIP4P2			PDK3
	PIP5K1A			KIF21A
	PIR			STMP1
	PISD			BCKDK
	PITHD1			ETS1
	PITPNC1			IFNA21
	PITPNM1			CLEC17A
	PITPNM2			LINC02158
	PITRM1			PANK3
	PITX1			RAB11FIP4
	PJA1			COG4
	PJA2			SMYD3
	PKD2			ELOVL3
	PKIA			TCTEX1D2
	PKIG			NPM1P27
	PKN1			DDAH2
	PKN3			ZNF175
	PKP4			ATP6V0A4
	PLA2G15			ULK4
	PLA2G4B			MIR300
	PLA2G7			MAF1
	PLAAT2			FGD4
	PLAAT3			MPND
	PLAAT4			POLR2E
	PLAC8			SPINDOC
	PLAC9			TCFL5
	PLAGL1			MAP3K2
	PLAUR			REEP6
	PLBD1			DCTN5
	PLBD2			BRWD1P2
	PLCB1			IPPK
	PLCB2			EIF4E
	PLCB3			LAMA3
	PLCD1			SLC5A3
	PLCG1			SEC61G
	PLCG2			RPL23A
	PLCL2			AC090527.3
	PLD2			RAB3D
	PLD5			SIM2
	PLEC			FLII
	PLEK			AQP4
	PLEKHA2			CRPPA
	PLEKHA3			SYNGR1
	PLEKHA4			RTKN2

	PLEKHA5			PLCD1
	PLEKHA7			LMF2
	PLEKHB2			GABPA
	PLEKHG1			NAA25
	PLEKHG2			ZCCHC8
	PLEKHG3			CAVIN2
	PLEKHH2			NUDT14
	PLEKHJ1			CIP2A
	PLEKHM1			RPAP1
	PLEKHM2			TRAPPC2L
	PLEKHO1			LINC02029
	PLEKHO2			RB1
	PLGRKT			RMDN1
	PLIN2			KLF5
	PLK2			AL021707.1
	PLK3			TP53I11
	PLLP			ATP13A4
	PLOD1			AOC3
	PLP2			CDKAL1
	PLPBP			ELOVL5
	PLPP1			ABCB10
	PLPP3			CASP6
	PLPPR2			ZNF484
	PLSCR1			SRP19
	PLSCR4			TWSG1
	PLXNA1			AL096711.2
	PLXNA2			C2CD4B
	PLXNA3			FAM193B
	PLXNB1			AC092803.1
	PLXNB2			MIR3685
	PLXNC1			COLEC11
	PLXND1			AC131011.2
	PMEPA1			PDK2
	PML			UBE2S
	PMPCA			TCAP
	PMPCB			DXO
	PMS1			CAPN12
	PMS2CL			PSMD1
	PMS2P6			MAL2
	PMS2P7			RSL1D1
	PMVK			MIR3142HG
	PNISR			PLPP3
	PNLDC1			DARS1
	PNN			DIS3L
	PNP			CHMP4C
	PNPLA2			TIMM13
	PNPLA4			KANK2
	PNPLA6			DNAH5
	PNPO			MICOS13
	PNPT1			TMEM87B
	PNRC1			SETD1A
	PODXL			PRADC1
	POF1B			EMC4
	POFUT2			MIF4GD
	POGLUT1			HNRNPH3
	POGZ			PSMB6
	POLA1			TNKS1BP1
	POLA2			NUDT16L1
	POLB			ZNF529
	POLD2			ACVR1

	POLDIP3			CDC42BPG
	POLE			GRAMD1B
	POLG			WASHC4
	POLM			MCFD2
	POLR1A			ZBTB39
	POLR1D			DCP1B
	POLR1E			ANKRD45
	POLR2B			NPL
	POLR2D			IGSF3
	POLR2E			DHX34
	POLR2G			AL355987.3
	POLR2K			SNORA74B
	POLR2L			HMG20A
	POLR2M			PRDM6
	POLR3C			PITPNM3
	POLR3GL			SPINT2
	POM121			MGMT
	POM121C			ACP1
	POMGNT1			MAPK1
	POMT1			AL358472.5
	POMT2			ALDH16A1
	POMZP3			PLXNA1
	POP4			SERPINB6
	POP5			GPRC5B
	POP7			ITGB4
	POPDC3			COP1
	POR			AP000944.4
	POU2AF1			L3MBTL3
	POU2F2			CRACD
	POU6F1			CCDC157
	PPA1			STX7
	PPARA			CLN5
	PPARD			CDC123
	PPARG			RBFOX2
	PPARGC1B			ABCA3
	PPDPF			SLC8B1
	PPFIA1			APEX1
	PPFIA4			FAM86C2P
	PPFIBP2			FAM210B
	PPHLN1			AC011939.2
	PPIA			LSM11
	PPIB			RIC1
	PPID			AL450992.2
	PPIE			SIX1
	PPIG			ZBTB32
	PPIH			SPTB
	PPIL1			AL645939.2
	PPIL4			SLC49A4
	PPL			LINC01215
	PPM1A			KIF26B
	PPM1F			ID4
	PPM1H			MTCO3P11
	PPM1K			LPXN
	PPM1M			TMEM106A
	PPME1			TMEM14A
	PPOX			TINAGL1
	PPP1CA			ASH1L-AS1
	PPP1R10			GRM8
	PPP1R11			ZNF394
	PPP1R12A			MTIF3

	PPP1R12B			PPP2R5B
	PPP1R12C			RARS2
	PPP1R13B			SIAH1
	PPP1R14A			MUC5AC
	PPP1R14B			HES1
	PPP1R14B-AS1			KCNQ3
	PPP1R15A			AC108749.1
	PPP1R16B			MTFR1L
	PPP1R18			CIB2
	PPP1R2			C5orf63
	PPP1R3C			VCPKMT
	PPP1R8			TRAK1
	PPP1R9A			BEST3
	PPP1R9B			ZZEF1
	PPP2CA			MTMR2
	PPP2R2C			GNAQ
	PPP2R2D			AL163051.1
	PPP2R3C			RAD52
	PPP2R5B			FXYD5
	PPP2R5D			RAD54L2
	PPP3CC			GNG10
	PPP3R1			ACADSB
	PPP4C			POLR2J
	PPP4R1			KBTBD2
	PPP4R2			SLC25A20
	PPP4R3A			TRIM15
	PPP4R3B			SETX
	PPP5C			CDK5RAP2
	PPP6C			PIR
	PPP6R2			SNX25
	PPP6R3			SMIM20
	PPRC1			DOCK7
	PPTC7			TMEM126B
	PQBP1			MTCL1
	PRADC1			AC242842.3
	PRAF2			SDHC
	PRAME			EFL1P1
	PRDM1			PLXNB1
	PRDM11			SERPINA1
	PRDM15			MCM4
	PRDM2			PTOV1
	PRDM4			GEMIN8
	PRDX1			TRAPPC6B
	PRDX2			KIF13B
	PRDX5			SDHD
	PRDX6			PWP1
	PREB			ILF2
	PRELID1			CORO1B
	PRELID2			AC005072.1
	PREPL			SLC9A2
	PRICKLE2			FAM126A
	PRKAB1			AL161421.1
	PRKACB			AC008517.1
	PRKAG2			RPSA
	PRKAR1B			AGBL2
	PRKAR2A			TBC1D2B
	PRKCA			CDCA8
	PRKCE			NME2
	PRKCZ			MIF
	PRKD1			MAN2A1

	PRKRIP1			TAOK1
	PRKX			PDE3B
	PRKY			RRP7A
	PRMT1			AP000944.2
	PRMT2			NUDCD3
	PRMT5			CALB2
	PRMT7			NUBPL
	PRMT9			TAF1C
	PRPF19			SCRN1
	PRPF31			ABHD15
	PRPF38A			GPX4
	PRPF38B			NUDCD2
	PRPF39			ACTR6
	PRPF4			ST7-OT4
	PRPF4B			SSR2
	PRPF6			PACSIN3
	PRPF8			CYS1
	PRPS1			ATAD2
	PRPS2			ZBTB42
	PRPSAP2			AAMP
	PRR13			AC083973.1
	PRR13P5			FLOT2
	PRR14			JAZF1-AS1
	PRR14L			REEP1
	PRR3			AC090844.3
	PRRC1			SZRD1
	PRRC2A			DHRS3
	PRRG1			MARF1
	PRRG4			AL138963.2
	PRSS16			AL590068.3
	PRSS23			GSTO2
	PRSS8			PDP1
	PRTG			RRM2
	PRX			AP001372.2
	PRXL2B			ZNHIT2
	PSAP			B4GALT6
	PSAT1			FAM126B
	PSEN1			CREG1
	PSEN2			AC010336.8
	PSENEN			TRAF3
	PSMA4			STAT5A
	PSMA7			EIF2B5
	PSMB1			SCRIB
	PSMB10			NBDY
	PSMB2			FOXO3
	PSMB3			AC008870.3
	PSMB4			CLK2
	PSMB5			EVI5
	PSMB7			SERPINE1
	PSMB8			GTSF1
	PSMB9			UBE4A
	PSMC1			ARHGEF39
	PSMC1P1			B4GALNT3
	PSMC3			BAP1
	PSMC4			KIF1A
	PSMC5			CCNH
	PSMC6			WASH3P
	PSMD1			TIMM21
	PSMD11			SLC20A1
	PSMD13			CTDSP1

	PSMD2			ZNF783
	PSMD3			VAMP2
	PSMD4			SUPT16H
	PSMD5			PALM2AKAP2
	PSMD6			TOP3A
	PSMD7			AC092120.1
	PSMD8			FAM102B
	PSME3			S100A10
	PSMF1			CCNY
	PSMG2			AP000845.1
	PSPC1-AS2			AKAP6
	PSTPIP2			LINC01126
	PTAFR			C14orf132
	PTBP1			COQ9
	PTBP2			FBXL6
	PTBP3			BMP8B
	PTCD3			ICA1
	PTDSS2			ASRGL1
	PTEN			TBL1X
	PTENP1			C19orf54
	PTGER2			ACADVL
	PTGIS			NF2
	PTGR1			INSL4
	PTH1R			YY1AP1
	PTK2			H2AC11
	PTK2B			USP21
	PTK6			CLDND1
	PTK7			FEZ2
	PTMAP5			LHFPL2
	PTMS			CLDN12
	PTOV1			ZNF605
	PTP4A1			RABGAP1
	PTP4A3			CHST10
	PTPA			TOMM5
	PTPDC1			TMEM53
	PTPN1			AL118516.1
	PTPN13			ANKHD1-EIF4EBP3
	PTPN2			MCTP1
	PTPN21			KDELR3
	PTPN3			NUP43
	PTPN6			TRMT2B
	PTPN9			C19orf53
	PTPRB			AC147651.1
	PTPRC			ME2P1
	PTPRD			AL359878.1
	PTPRE			HEATR5B
	PTPRG			TRPV4
	PTPRK			TRMT44
	PTPRM			AL161454.1
	PTPRN2			NAE1
	PTPRZ1			DHTKD1
	PTS			SH3YL1
	PTTG1IP			PTCD3
	PUDP			RNVU1-30
	PUF60			RNU6-33P
	PUM2			TPRG1L
	PUM3			MEF2A
	PURPL			AL354920.1
	PUS1			HNRNPUL2-BSCL2
	PVR			SLC37A4

	PWP2			CSNK1A1
	PWWP2A			SNTA1
	PWWP3A			GABPB1
	PWWP3B			SRP9P1
	PXDC1			SURF1
	PXDN			ZNF519
	PXK			TP53RK
	PXN			ABCB1
	PYCR2			RPL7P9
	PYGB			SMG5
	PYGL			PRUNE2
	PYGO2			IFNA8
	PYROXD1			HRC
	QARS1			CEP112
	QKI			MIR3190
	QPCT			AP000311.1
	QPCTL			AC116366.2
	QRICH1			AC002377.1
	QSER1			CDKL5
	QSOX1			GNAI3
	QSOX2			RPS29P14
	QTRT1			AC092958.3
	R3HCC1			PPP1R3B
	R3HDM2			BAD
	R3HDM4			LEPR
	RAB10			CSGALNACT1
	RAB11B			PKD1L1
	RAB11FIP1			BRIP1
	RAB11FIP3			PEX13
	RAB11FIP5			AP002992.1
	RAB13			TMEM256
	RAB15			IL17RD
	RAB17			ACAD9
	RAB1B			SLC35E2B
	RAB20			MPST
	RAB21			SLC6A13
	RAB24			MRPS21
	RAB25			BLM
	RAB27A			SAFB
	RAB29			NSFL1C
	RAB2B			MIA
	RAB30			LINC00326
	RAB32			TRPA1
	RAB33B			GRIK5
	RAB34			PDE4D
	RAB35			SMIM19
	RAB3GAP2			STX16-NPEPL1
	RAB40B			MIR5047
	RAB40C			ZNF185
	RAB4A			TLCD2
	RAB4B			ACOT9
	RAB5C			STK17A
	RAB6A			RPSAP58
	RAB6C			CHID1
	RAB7A			HSPE1P11
	RAB7B			ATG12
	RAB8A			CCDC69
	RAB8B			MOCS3
	RABAC1			SNORA28
	RABGAP1			MAML2

	RABGAP1L			LINC01271
	RABGGTA			AL731571.1
	RABIF			MTOR
	RABL2A			PLEKHG3
	RABL3			DPH2
	RABL6			C1QTNF1
	RAC2			CRKL
	RACK1			AL137781.1
	RAD18			AL160408.2
	RAD23A			AP001062.2
	RAD54L2			FAM83F
	RAE1			AP1S3
	RAET1G			ANKMY1
	RAET1L			AC067968.1
	RAF1			LGALS1
	RAI2			ZNF862
	RALB			PEX2
	RALBP1			H2AC16
	RALGAPA2			LYPD3
	RALGAPB			TRIP12
	RALGDS			B4GALT3
	RALGPS2			MYL5
	RALY			RSRP1
	RAN			AC004264.1
	RANBP1			ART3
	RANBP10			STEAP2
	RANBP3			PTMS
	RANBP9			SGMS1-AS1
	RANGAP1			TP53INP2
	RAP1A			PTMAP2
	RAP2C			LRRCC1
	RAPGEF1			DNAL1
	RAPGEF2			PPCDC
	RAPGEF3			UCP2
	RAPGEF5			P4HTM
	RAPGEFL1			EIF2B1
	RASA1			AC092868.3
	RASA3			AGAP1
	RASA4			WNK3
	RASA4B			EIF2AK3
	RASD2			TMEM254
	RASGEF1B			NAGA
	RASGRF2			CEP63
	RASIP1			GSE1
	RASL11A			FEZ1
	RASL12			ABCE1
	RASSF1			PNKD
	RASSF10			PIK3C3
	RASSF2			YKT6
	RASSF7			GRK3
	RBBP4			NUTF2
	RBBP6			ANXA2R
	RBBP8			CC2D2A
	RBBP9			AC007780.1
	RBCK1			ZBTB41
	RBFOX2			FAM189B
	RBM10			MPHOSPH8
	RBM11			NCL
	RBM12			HAUS1
	RBM14			HENMT1

	RBM15B			UQCRC2
	RBM17			SCOC
	RBM19			ZC3H3
	RBM22			PSMD11
	RBM26			PNRC2
	RBM28			RBM8A
	RBM3			AC011448.1
	RBM33			LINC01285
	RBM34			GOLPH3
	RBM39			TRPC1
	RBM43			ENTPD3
	RBM47			TTI2
	RBM5			DUS3L
	RBM6			PHF12
	RBM7			PLD3
	RBMS1			UQCR11
	RBMX2			QPCT
	RBMXL1			COX19
	RBP4			C8orf31
	RBPMS			LRIG2
	RBSN			MCM8
	RBX1			CNKSR2
	RC3H2			NUPR1
	RCAN1			RFPL4A
	RCBTB1			AC019117.3
	RCBTB2			AL035460.1
	RCC1L			LDC1P
	RCC2			AC009949.1
	RCE1			GMDS
	RCOR1			NFATC2
	RDH10			MMP15
	RDH11			MZT2A
	RDH14			SNHG25
	RDM1			NEK7
	RDX			TM9SF2
	REC8			SPAST
	RECK			RNVU1-25
	RECQL4			DUT
	REL			CDK11B
	RENBP			COPRS
	REPS2			COX4I1
	RERE			COPS5
	RESF1			BLOC1S1
	REST			MT1X
	RETREG1			EMB
	RETREG3			POMGNT1
	RETSAT			NDFIP1
	REV1			SLC25A10
	REX1BD			NEK10
	REXO4			BTBD2
	RFC1			IGHE
	RFLNB			SDSL
	RFNG			AP1S2
	RFT1			GLG1
	RFTN1			FOXP2
	RFX1			ANKHD1
	RFX7			ARHGAP17
	RFXANK			PCNA
	RGCC			TJP3
	RGL1			SETD1B

	RGL2			PTN
	RGL4			RBM25
	RGMB			PRPS1
	RGP1			OMA1
	RGPD1			GPC1
	RGPD2			DHX29
	RGPD3			LINC01534
	RGPD5			MRPS34
	RGPD6			RASSF4
	RGPD8			LIPG
	RGS12			RASSF6
	RGS18			RALGPS1
	RGS2			NFATC3
	RGS3			IGBP1
	RGS4			ETNK1
	RGSL1			CD99P1
	RHBDD1			RNA5S9
	RHBDD2			ZNF720
	RHBDF1			TCF7L2
	RHBDF2			PLEKHN1
	RHOA			ERO1A
	RHOB			NBPF26
	RHOC			TOR3A
	RHOG			TPR
	RHOH			CACUL1
	RHOJ			AC105219.1
	RIBC1			DDX42
	RIC1			MORN2
	RIC8B			AC025431.1
	RICTOR			AC007032.1
	RILPL1			DOK4
	RIMKLB			PPOX
	RIMS2			EMSY
	RIMS3			SPR
	RIN2			PGF
	RING1			MAK
	RIOK2			TOMM70
	RIOK3			ECPAS
	RIPK1			RANBP3
	RIPK2			RPAIN
	RIPOR1			AP003419.1
	RIPOR2			TMEM38B
	RIPOR3			UBR5
	RIPPLY3			NDUFB9
	RLF			VPS39
	RLIM			GARS1
	RMDN1			SKA3
	RMDN3			ARID1B
	RMND1			CENPM
	RMND5A			DDX50
	RMND5B			IL20RB
	RN7SL396P			CCT2
	RN7SL689P			LRFN1
	RNA5S9			ST6GALNAC2
	RNA5SP141			LRRC41
	RNA5SP145			CHKB
	RNA5SP225			ENTPD6
	RNA5SP298			ZNF827
	RNA5SP481			SPC24
	RNASE2			TCEA3

	RNASEH1			URB2
	RNASEH2B			ZMYM1
	RNASEH2C			UBE2G1
	RNASEL			SIDT1
	RND1			DLG1
	RND3			LRCH4
	RNF113A			RAP1GDS1
	RNF121			SYNM
	RNF122			DDX24
	RNF13			NDUFB8
	RNF139			LURAP1L-AS1
	RNF144A			AC034236.1
	RNF144B			NPM3
	RNF145			CHRNA7
	RNF149			CASR
	RNF152			MARC1
	RNF166			TSPAN18
	RNF167			NLRP4
	RNF168			OR52K1
	RNF170			AC092168.1
	RNF175			AC022092.1
	RNF185			HSPB9
	RNF19B			ADPGK-AS1
	RNF20			AC010501.2
	RNF213			AC011369.2
	RNF216			ARMC10P1
	RNF216P1			CAVIN4
	RNF220			POP5
	RNF224			ZNF594
	RNF227			ZHX3
	RNF25			ANAPC15
	RNF34			BMS1P4
	RNF4			AC015813.2
	RNF40			GRM5
	RNF44			ERCC6L
	RNF5			TIMM8B
	RNF8			LONRF3
	RNGTT			UBR3
	RNH1			SNORA47
	RNMT			HMGB1P3
	RNPEP			CNTF
	RNPS1			TMEM65
	RNU4-1			STK24-AS1
	RNU4ATAC			DCUN1D4
	RNU5A-1			NANS
	RNU6-1			LINC00240
	RNU6-2			SNORC
	RNU6-36P			FANCF
	RNU6-37P			HABP2
	RNU6-5P			ALKBH5
	RNU6-9			AL034430.1
	RNU6ATAC			MBOAT7
	RNVU1-28			IL17RC
	RNVU1-2A			PQBP1
	RNVU1-31			SOX13
	ROBO3			ZNF664
	ROBO4			RPS26
	ROCK1			ZNF777
	ROCK2			RPL23AP82
	ROMO1			CSNK1G1

	RORA			WNK1
	RP11-173M1.8			SNORD15B
	RP11-175O19.4			LINC02035
	RP11-206L10.3			IPO9
	RP11-211G3.2			PHLDA2
	RP11-401P9.4			COPB1
	RP11-435O5.2			AC120024.1
	RP11-474N8.8			SUPT20H
	RP11-485G4.2			AC090114.3
	RP11-680G24.5			TBC1D9B
	RP1-168P16.2			CCL22
	RP11-706O15.3			LINC00511
	RP11-706O15.5			EXOC6B
	RP1-178F10.3			AC019186.1
	RP11-872J21.5			CSF2RA
	RP11-89K10.1			SLC39A1
	RP1-71H24.6			YES1
	RP2			SEC11A
	RP3-323A16.1			RPUSD1
	RP3-368A4.5			MORC3
	RP5-1039K5.19			CDC6
	RP9P			NDUFA3
	RPA1			PROM2
	RPA2			CINP
	RPA3			TOX4
	RPAIN			VPS9D1-AS1
	RPAP2			ENSA
	RPAP3			AC016405.1
	RPGR			C2-AS1
	RPH3A			MAP2K5
	RPH3AL			LINC00973
	RPIA			C1orf198
	RPL10			FASTKD1
	RPL10A			CDC42BPB
	RPL10P16			EIF3H
	RPL10P3			STN1
	RPL10P6			CROT
	RPL12			PINK1
	RPL12P4			KIAA0408
	RPL13			RFC4
	RPL13A			LRRC75B
	RPL13AP20			ARRB1
	RPL13AP25			WDR46
	RPL13AP5			SEPTIN4
	RPL13AP7			CYP21A2
	RPL14			HSPA14
	RPL15			AL390719.1
	RPL15P2			FGF18
	RPL18			MTMR11
	RPL18A			CDR2
	RPL19			TRIM34
	RPL21			SCAND2P
	RPL22P1			PJA1
	RPL23AP53			TWNK
	RPL23AP61			ZNF574
	RPL24			MED12
	RPL26			ALAS1
	RPL26P19			MCUR1
	RPL3			UBB
	RPL31			AC005258.1

	RPL31P63			MAT2A
	RPL32			TBC1D4
	RPL32P3			DAD1
	RPL34			COLEC10
	RPL34P18			TACC2
	RPL35A			CDS1
	RPL36			EPS15
	RPL36AL			NIBAN1
	RPL36AP26			CDH2
	RPL37P2			CPSF1
	RPL38			THRB-IT1
	RPL39			CCDC124
	RPL39P3			UGT2A3
	RPL4			LINC01002
	RPL41P2			ZFHX4
	RPL5			RBM7
	RPL6			ZNF281
	RPL6P27			FBXW7
	RPL7			ZNF649
	RPL7A			USP46
	RPL7AP66			TCF3P1
	RPL7P6			CIAPIN1
	RPL7P9			H2AC5P
	RPL8			AL355377.1
	RPL9			SERGEF
	RPL9P29			BDNF-AS
	RPL9P7			CREB3L4
	RPLP0			NPC1L1
	RPLP1			MIIP
	RPLP1P6			CYP20A1
	RPLP2			DERL1
	RPN1			CFAP298
	RPP25			ZGPAT
	RPRD1A			CALCA
	RPRD1B			CAPZA1
	RPRD2			ZFYVE27
	RPS10			ALG5
	RPS11P5			ZFP41
	RPS13			RIPOR1
	RPS13P2			PLSCR2
	RPS14			LYPLA1
	RPS15A			LINC01089
	RPS15AP1			ST6GALNAC1
	RPS16			MRPS5
	RPS18			AC068338.2
	RPS19			APOM
	RPS19BP1			TMED8
	RPS23			ZC3H12D
	RPS23P8			AL805961.1
	RPS24			ANKRD36C
	RPS24P8			AC104472.1
	RPS26P11			CLASRP
	RPS26P15			FADD
	RPS26P31			DMTF1
	RPS26P47			COQ10B
	RPS27			MAP7
	RPS27A			CD163
	RPS27P29			CREB3L2
	RPS28			TOMM7
	RPS29			TMSB4XP8

	RPS2P35			ZSCAN20
	RPS2P5			CMTM7
	RPS2P55			SSH3
	RPS3			LAPTM4A
	RPS3A			ABLIM3
	RPS5			RALGAPA2
	RPS6			THAP1
	RPS6KA2			TBC1D9
	RPS6KA5			HTR3E
	RPS6KA6			WDR5B
	RPS6KB1			ADAMTSL2
	RPS6KB2			TAF9B
	RPS6KC1			ZBTB33
	RPS7P1			TICRR
	RPS7P10			AC067945.1
	RPS7P11			ANGPTL1
	RPS8			SYNJ2BP
	RPS9			RN7SKP9
	RPSA			TMEM236
	RPSAP12			ARL15
	RPSAP4			FZD1
	RPSAP47			RANBP6
	RPSAP54			EPHB4
	RPTOR			TXN2
	RPUSD2			SYNRG
	RRAD			AC073508.2
	RRAS			BBS2
	RRAS2			TOMM6
	RRBP1			LINC01467
	RREB1			AJ003147.1
	RRM2			CCL4
	RRN3			SGMS2
	RRP1			DDX47
	RRP12			CEP41
	RRP36			ZSWIM4
	RRP7A			NDUFB11
	RRP7BP			AF127936.1
	RRP9			EXD2
	RSAD1			BACE1-AS
	RSAD2			CLOCK
	RSBN1L			GGT1
	RSF1			HYKK
	RSPO3			TTPAL
	RSPRY1			PHF10
	RSRC2			KCNS1
	RTCB			SLC25A36
	RTF1			LINC01226
	RTKN			LITAF
	RTKN2			BICD1
	RTL10			EID2B
	RTL5			FADS3
	RTL8A			SPDYA
	RTN3			GALNT14
	RTN4			DKK3
	RTP4			PYCR1
	RUBCN			KIF13A
	RUFY2			ZBED9
	RUFY3			LIMD2
	RUNDC3A-AS1			UNC13B
	RUSC2			NUP107

	RUVBL2			CLCN5
	RWDD4			CISD2
	RXRA			EI24
	RXRB			PRSS8
	RYK			SLC7A5P1
	S100A11			SPATA6
	S100A12			SF3B5
	S100A13			INO80B-WBP1
	S100A14			SNORA63B
	S100A16			LMBRD1
	S100A2			MLX
	S100A8			MAPK8IP2
	S100A9			NSMCE1
	S100P			VEGFB
	S100PBP			TAGLN2P1
	SAA1			AC037459.2
	SAA2			PHF20
	SACS			SNX30
	SAE1			CTSZ
	SAFB			KLHL15
	SAFB2			SNX11
	SAMD4A			ABCA12
	SAMD9			TTLL5
	SAMD9L			ING1
	SAMHD1			ZNF202
	SAMM50			KIF22
	SAP18			AC092073.1
	SAP30			ERCC6
	SAP30BP			CYREN
	SAP30L			UPF2
	SAR1B			SCYL1
	SARAF			FAM81A
	SARS1			AC008581.2
	SART1			ICAM3
	SASH1			VAMP7
	SAT1			SPTAN1
	SBDSP1			CASZ1
	SBF1			EIF1
	SBNO1			SNORA16A
	SC5D			SNORA16A
	SCAF11			TMED2-DT
	SCAF4			KRT8
	SCAMP1-AS1			NCF2
	SCAMP3			SNHG26
	SCAND1			MANSC1
	SCAP			MYBPC1
	SCARA3			AL139100.2
	SCARB1			SNX6
	SCARB2			CDC20B
	SCD5			AC139491.2
	SCDP1			AC006065.4
	SCGB1A1			C20orf141
	SCLT1			AC098934.4
	SCMH1			ZFP30
	SCML1			PARM1
	SCN2A			AC092747.2
	SCN4B			CSPG5
	SCN9A			ADNP2
	SCNN1B			DCUN1D3
	SCNN1G			TMEM33

	SCO1			SFXN2
	SCOC-AS1			CPAMD8
	SCRN1			CEP162
	SCRN2			AL365181.2
	SCYL1			AC005899.5
	SCYL2			ARFGAP2
	SDAD1			ZYG11B
	SDAD1P1			EPDR1
	SDC3			BYSL
	SDCBP			MAP1S
	SDCCAG8			H3C4
	SDE2			MSN
	SDF2L1			ABTB1
	SDHA			RNVU1-21
	SDHAF1			DTX3
	SDHAF2			AC012513.3
	SDHAF3			TMEM187
	SDHAP1			LONRF2
	SDHB			INTS8
	SDHD			ZFYVE1
	SEC13			SERTAD4
	SEC16A			NOS3
	SEC22B			LINC01003
	SEC22C			RFPL2
	SEC23A			MXI1
	SEC23IP			OGA
	SEC24C			THNSL1
	SEC31A			PLXNA4
	SEC31B			GCNT3
	SEC61A1			C6orf223
	SEC61A2			NID1
	SEC62			ANAPC5
	SEC63			TNNC1
	SECISBP2L			RTL10
	SECTM1			KRT10
	SEL1L			SDHA
	SELENBP1			LTBR
	SELENOH			WDR35
	SELENOM			IMMP2L
	SELENON			SIRT5
	SELENOO			MEAF6
	SELENOS			TTC9
	SELENOW			WWC3
	SELL			AC004870.2
	SELPLG			SNRPGP2
	SEM1			ZNF710-AS1
	SEMA3A			GPR35
	SEMA3B			TANGO6
	SEMA3C			VASH1
	SEMA3F			C7orf50
	SEMA4A			CREB1
	SEMA4B			RN7SKP175
	SEMA4C			KPNA4
	SEMA6B			ZNF488
	SEMA6D			PAIP1P1
	SENP3			HCG25
	SENP5			VEGFD
	SENP6			NDUFA12
	SEPHS1			ATXN7L2
	SEPHS2			RPS19BP1

	SEPTIN11			SLC22A18AS
	SEPTIN4			CNOT10
	SEPTIN6			INHBE
	SEPTIN7P2			VXN
	SEPTIN8			CBX2
	SEPTIN9			SGO1
	SERAC1			TIRAP
	SERBP1			ZNF595
	SERF2			HMGN4
	SERGEF			CCDC50
	SERINC1			AC073046.1
	SERINC5			ZNF195
	SERP1			GLIPR1
	SERPINA1			GPX2
	SERPINA6			BRPF3
	SERPINB1			AP001528.1
	SERPINB2			KRT8P12
	SERPINB6			SMIM25
	SERPINB8			PFKL
	SERPINB9			CD14
	SERPINB9P1			ZBTB46
	SERPINE2			FIG4
	SERPING1			TUT1
	SERTAD1			ZNF491
	SERTAD2			RSPH3
	SERTAD3			TRPS1
	SESN1			FOXN2
	SESN2			RNU7-196P
	SETD1A			AC009242.1
	SETD3			VDAC2
	SETD5			PTPN13
	SETD6			CELF1
	SETD7			GPR83
	SETDB1			PACS2
	SEZ6L2			GALT
	SF1			NTPCR
	SF3A2			AC145207.5
	SF3A3			CCDC9B
	SF3B1			B4GALNT2
	SF3B2			AMN1
	SF3B4			PRKACA
	SF3B5			GSS
	SF3B6			SMARCB1
	SFI1			SERPINA10
	SFMBT1			PSMC1P2
	SFN			CSNK1G2P1
	SFPQ			AC090673.1
	SFR1			AC097636.2
	SFRP4			NDEL1
	SFSWAP			PGPEP1
	SFT2D1			SREK1IP1
	SFTPA1			ADGRG6
	SFTPA2			KCTD9
	SFTPB			CT83
	SFTPC			TALDO1
	SFTPD			FANCL
	SFXN3			LARP4B
	SFXN4			FGD2
	SFXN5			SUPV3L1
	SGCA			TRIM6-TRIM34

	SGCE			AC027309.2
	SGIP1			RNF14
	SGK1			GDAP1
	SGMS1			MAOA
	SGPL1			GLB1L2
	SGPP2			REEP5
	SGSH			ZBED4
	SGSM2			SH3GLB1
	SGSM3			BICD2
	SGTA			AP002387.2
	SH2B1			TNPO3
	SH2B3			AC007849.1
	SH3BGRL2			MPV17L
	SH3BGRL3			PREPL
	SH3BP2			SCAF11
	SH3BP5			FJX1
	SH3BP5-AS1			ZSCAN25
	SH3D19			KPNA5
	SH3GL1			UBA3
	SH3PXD2A			LINC02331
	SH3PXD2B			RPL39
	SH3RF1			PRKCE
	SH3RF3			HHEX
	SH3TC1			MRPL34
	SH3TC2			TXN
	SH3YL1			SYT1
	SHANK3			TCTN3
	SHB			DGKG
	SHC1			RBM4
	SHC2			DNALI1
	SHE			PUM1
	SHFL			TMEM263
	SHISA5			ZNF43
	SHISA9			SLC1A4
	SHKBP1			ITGA9-AS1
	SHOC2			PSMA1
	SHQ1			ITFG1
	SIAH2			ZNF205
	SIDT2			SMIM14
	SIGLEC16			CWC15
	SIK1			PRPF3
	SIK1B			KIFC3
	SIK2			HERC3
	SIL1			NEDD4L
	SIMC1			MMS19
	SIN3A			AC022167.2
	SIN3B			SPRED2
	SIRPA			NUCB2
	SIRPB1			ZNF737
	SIRT1			AL139174.1
	SIRT5			LEPROTL1
	SIRT7			FIZ1
	SIVA1			LRRC37B
	SIX1			RN7SL674P
	SKA2			FAM53B
	SKIV2L			TLE3
	SKP1			SETMAR
	SLAIN2			IRF8
	SLAMF7			AC093155.3
	SLBP			DEPDC7

	SLC10A3			PCMT1
	SLC12A2			SLU7
	SLC12A4			AC092279.2
	SLC12A5			CEBPG
	SLC12A6			RPS19P1
	SLC12A7			RIT1
	SLC13A1			TP53TG1
	SLC15A2			UCK2
	SLC16A4			RPRD2
	SLC16A5			AKR7A2
	SLC16A7			WDR55
	SLC18B1			LINC00158
	SLC19A2			AC078923.1
	SLC22A15			TMED7
	SLC22A17			AC087190.3
	SLC22A23			TMEM109
	SLC24A4			RHOBTB3
	SLC25A1			TMEM120B
	SLC25A11			CTSL
	SLC25A17			HAT1
	SLC25A19			NDUFV2-AS1
	SLC25A21			SMAD6
	SLC25A23			RAB9A
	SLC25A25			NCKAP5
	SLC25A26			SNF8
	SLC25A28			IREB2
	SLC25A29			TMEM218
	SLC25A3			KAT2B
	SLC25A30			FLAD1
	SLC25A33			SLC38A10
	SLC25A36			RPL11
	SLC25A4			MSH2
	SLC25A44			CLDN4
	SLC25A5			AC104035.1
	SLC25A6			PRKAA1
	SLC26A4			IL23A
	SLC26A6			C1GALT1C1
	SLC26A8			AL117339.5
	SLC27A1			DUSP14
	SLC27A3			AP2A2
	SLC28A2			WDR61
	SLC29A1			AARS1
	SLC2A1			RTN4
	SLC30A1			ANKRD36
	SLC30A4			ARHGAP12
	SLC31A1			TXNL4B
	SLC34A2			LINC00205
	SLC35A3			ELOA
	SLC35A4			RPS2P44
	SLC35B2			CLDN2
	SLC35C2			NRBP2
	SLC35E1			FGFR1OP2
	SLC35F1			FAM193A
	SLC35F2			FCGRT
	SLC35F3			RPSAP9
	SLC35G2			DST
	SLC37A4			DYNC1LI1
	SLC38A10			PRIMPOL
	SLC38A2			IFT81
	SLC38A5			AC091959.3

	SLC39A1			AC016747.1
	SLC39A13			FIBP
	SLC39A3			GNB5
	SLC39A6			AC138969.1
	SLC39A7			CHKA
	SLC39A8			SSUH2
	SLC40A1			NBAS
	SLC41A1			SNORD101
	SLC41A2			LIX1L
	SLC41A3			CCRL2
	SLC43A1			SLC43A3
	SLC44A1			ZMYM3
	SLC44A2			KDM1B
	SLC44A3-AS1			NOC3L
	SLC44A4			CBX1P3
	SLC48A1			FDPSP4
	SLC4A1AP			MIR3173
	SLC4A7			H3C5P
	SLC51A			PIGL
	SLC52A2			DTWD2
	SLC5A3			ULBP3
	SLC5A6			PAN2
	SLC66A2			EEA1
	SLC6A4			ECT2
	SLC6A8			AC099518.1
	SLC7A2			FKBP15
	SLC7A5			ANKS4B
	SLC7A7			RNASET2
	SLC8A1			AL358472.7
	SLC8B1			SNRNP25
	SLC9A1			SOCS6
	SLC9A2			ATR
	SLC9A3R2			MFSD6
	SLC9A4			ELOVL6
	SLC9A8			C17orf113
	SLC9B2			ENTPD4
	SLCO2B1			UBR1
	SLCO4A1			OR7E91P
	SLF2			FAT2
	SLFN11			PLXND1
	SLFN13			TGFB2
	SLFN5			FUT11
	SLIRP			PHLDB1
	SLIT2			PXMP2
	SLIT3			DZIP1
	SLK			CCPG1
	SLPI			ANKRD26P1
	SLTM			PLAAT3
	SLU7			RBM48
	SLX1A			AC022784.1
	SLX1B			AL590714.1
	SMAD3			CSTB
	SMAD7			ZNF101
	SMAP2			FAM229B
	SMARCA1			RDH10
	SMARCA4			AL158196.1
	SMARCAL1			PIEZO2
	SMARCB1			AC009226.1
	SMARCD1			GPN1
	SMARCD2			SGCB

	SMARCD3			TLCD4-RWDD3
	SMC1B			NBN
	SMC2-AS1			ONECUT2
	SMC3			LINC01559
	SMCHD1			ERCC2
	SMCO4			IMMT
	SMDT1			AP003499.3
	SMG1P1			WDR26
	SMG1P2			BIN3
	SMG1P3			DNAH14
	SMG1P4			EIF4BP6
	SMG5			H2BC21
	SMG7			PSMB1
	SMG7-AS1			MYRIP
	SMG9			AC004494.1
	SMIM11A			TGFBR3
	SMIM11B			MLPH
	SMIM15			TDP1
	SMIM25			CYP3A4
	SMIM26			AJ003147.3
	SMIM27			DYNC1I2P1
	SMIM3			CPED1
	SMIM4			TBC1D23
	SMNDC1			L3MBTL2
	SMPD1			RAB43
	SMPD2			DPYSL4
	SMPD4			AREL1
	SMTN			PLEKHS1
	SMURF1			CRISPLD2
	SMYD3			MINDY2
	SMYD5			GABARAPL1
	SNAI1			LINS1
	SNAP47			CLDN7
	SNAPC2			SDR42E1
	SNCG			COPS7A
	SNED1			MLLT3
	SNF8			SRGAP2
	SNHG1			NUP133
	SNHG11			SMIM22
	SNHG12			RAC1
	SNHG14			NDUFC1
	SNHG15			SLC29A1
	SNHG17			UNC80
	SNHG19			WWP1P1
	SNHG25			RPL39L
	SNHG28			ATP5MD
	SNHG29			ZNF326
	SNHG3			MIR425
	SNHG32			FAM234A
	SNHG5			TRAF6
	SNHG7			SMIM31
	SNHG8			KTI12
	SNHG9			AC016596.1
	SNN			H3-3A
	SNORA11F			GOT1
	SNORA12			AK7
	SNORA3B			AHRR
	SNORA73B			CAP2
	SNORA79B			LONRF1
	SNRNP200			RIDA

	SNRNP27			SNTB2
	SNRNP35			EAF1
	SNRNP70			FAM83G
	SNRPA1			MED7
	SNRPC			ATAD3B
	SNRPD2			NFS1
	SNRPGP15			PTPA
	SNRPN			MMS22L
	SNTA1			MICU2
	SNU13			KLHDC8B
	SNUPN			PRPF4
	SNW1			RNVU1-24
	SNX10			NDRG1
	SNX11			HOXA-AS3
	SNX14			PRUNE1
	SNX16			HSPA9
	SNX17			AC009090.5
	SNX19			HSPB1
	SNX2			GINS1
	SNX21			NGFR
	SNX25			ADGRL3
	SNX27			COL20A1
	SNX3			AC099489.3
	SNX33			KEAP1
	SNX5			PRELID2
	SNX6			ISCU
	SNX8			CARD10
	SOAT1			SLC7A2
	SOCS1			IPCEF1
	SOCS2			CHGA
	SOCS4			ACTA2-AS1
	SOCS5			TUBA8
	SOCS7			UMLILO
	SOD2			MTND5P14
	SOD3			AL355312.4
	SOGA1			HOXB8
	SON			CFLAR-AS1
	SORBS3			RPL29
	SORL1			SWI5
	SOS1			SERPINA5
	SOS1-IT1			MFSD14C
	SOWAHC			MYL9
	SOX13			SMDT1
	SOX21			FAR2
	SOX4			AC022400.5
	SOX9			PINK1-AS
	SP100			RAB30-DT
	SP110			PSMB5
	SP140			WBP1
	SP140L			RTKN
	SP2			MUS81
	SP6			SPRY1
	SPAG1			RBBP8NL
	SPAG7			PSME2P2
	SPATA13			RPL5P34
	SPATA18			BLOC1S5-TXNDC5
	SPATS2L			CCDC90B
	SPC24			SELENOS
	SPC25			GLUL
	SPCS1			GNPTG

	SPCS2P4			BECN1
	SPDEF			BBS10
	SPECC1			SRGAP2C
	SPECC1L			THAP3
	SPEN			BORA
	SPG21			CARD8-AS1
	SPG7			SULT1C2
	SPHK1			TMEM147
	SPI1			ZFHX3
	SPIDR			AP000350.4
	SPIN1			STAG1
	SPINDOC			SEMA4C
	SPINT2			ARMCX5
	SPOP			TMEM14C
	SPOPL			IWS1
	SPOUT1			H2BC3
	SPPL2A			IRF2BPL
	SPRED1			TFE3
	SPRED2			ZNF586
	SPRR1A			RPS16
	SPRR2B			NIP7
	SPRR2D			CSNK1G2
	SPRY1			SLC25A37
	SPRY2			PFDN6
	SPRY4			HEATR5A
	SPRYD3			TMEM147-AS1
	SPRYD4			CRAMP1
	SPRYD7			HS6ST2
	SPSB1			RAB30
	SPTBN1			TNS4
	SPTLC2			TTLL11-IT1
	SQOR			ZNF773
	SQSTM1			DNAH8
	SRA1			KANK1
	SRBD1			RPS3AP26
	SRCAP			TNFRSF25
	SRD5A3			NMT1
	SREBF2			ANKZF1
	SREK1			FAM117B
	SRFBP1			NDUFB10
	SRGAP1			SENP1
	SRGAP2B			EDC4
	SRGAP2C			PTPRO
	SRGN			SLC35A1
	SRL			IFNA2
	SRP68			AL354702.1
	SRR			AC011586.1
	SRRD			AC093283.1
	SRRM2			AC010271.2
	SRRM3			ADCY3
	SRRT			GPATCH11
	SRSF5			RSRC1
	SRSF6			PAXIP1
	SRSF7			COX6C
	SRSF8			ZDHHC9
	SRSF9			BUD23
	SS18L1			IKBKG
	SSB			KCNJ2
	SSBP2			AGTRAP
	SSBP4			RFPL4AP6

	SSH1			DRAM2
	SSNA1			HPS6
	SSR1			SLC35A4
	SSR2			AL596244.1
	SSR4			WNK2
	SSRP1			SYT11
	SSTR2			EIF4G3
	ST20			BICRAL
	ST3GAL1			CCDC198
	ST3GAL3			AC013717.1
	ST6GAL1			SNX18P7
	ST6GAL2			DPH6-DT
	ST6GALNAC2			STRIP2
	ST6GALNAC4			ATP5IF1
	ST6GALNAC6			CCNK
	ST7L			SNX19
	STAB1			ENKD1
	STAG1			NARS2
	STAM			TAF10
	STAM2			Z68871.1
	STAP1			C17orf80
	STAP2			RPS14
	STARD10			PIGO
	STARD13			Z97832.2
	STARD3			LINC01138
	STARD3NL			CRYBB2P1
	STARD7			AC026403.1
	STARD8			FAM122B
	STARD9			LRRC75A
	STAT1			OSER1-DT
	STAT2			TLR2
	STAT3			RUNX2
	STAT4			COL17A1
	STAT5A			ZNF107
	STAT5B			SNORA38
	STAT6			ZC3H10
	STAU1			PIGX
	STAU2			AL137856.1
	STC1			AC133644.2
	STEAP1			TAF9
	STEAP4			MTHFS
	STIM1			SGMS1
	STIMATE			ATF6B
	STING1			KIAA1211L
	STIP1			METTL9
	STK10			AC008982.1
	STK11IP			RPS6KA2
	STK16			CCDC9
	STK17B			AC097634.4
	STK19			EPB41L4A
	STK25			AP4M1
	STK36			VKORC1L1
	STK38			AC006116.9
	STK38L			SDHB
	STK4			RCBTB2
	STK40			H2AC6
	STMN1			NUS1
	STMP1			PGGHG
	STN1			RAI14
	STOM			RNU5A-1

	STOML2			KREMEN1
	STON1			
	STRAP			
	STRIP1			
	STRN4			
	STT3B			
	STUB1			
	STX11			
	STX12			
	STX17			
	STX18			
	STX4			
	STX5			
	STXBP2			
	STXBP6			
	STYX			
	SUCNR1			
	SUGP1			
	SUGP2			
	SUGT1P4-STRA6LP			
	SULF2			
	SULT1A1			
	SULT1A3			
	SULT1B1			
	SUMF1			
	SUMF2			
	SUMO2			
	SUMO2P17			
	SUN1			
	SUN2			
	SUOX			
	SUPT16H			
	SUPT20H			
	SUPT3H			
	SUPT4H1			
	SUPT5H			
	SUPT6H			
	SURF1			
	SURF4			
	SURF6			
	SUSD1			
	SUSD3			
	SUSD6			
	SUZ12P1			
	SVBP			
	SWAP70			
	SYAP1			
	SYBU			
	SYF2			
	SYN1			
	SYNE1			
	SYNE3			
	SYNGAP1			
	SYNGR1			
	SYNGR2			
	SYNM			
	SYNPO			
	SYNPO2			
	SYPL1			
	SYT11			

	SYT13			
	SYVN1			
	SZRD1			
	TACC1			
	TACC2			
	TAF1			
	TAF10			
	TAF11			
	TAF13			
	TAF1C			
	TAF4			
	TAF5L			
	TAF7			
	TAFA2			
	TAGAP			
	TAGLN			
	TAGLN2			
	TALDO1			
	TANC1			
	TANC2			
	TANGO6			
	TAOK1			
	TAOK3			
	TAP1			
	TAP2			
	TAPT1-AS1			
	TARBP2			
	TARDBP			
	TARS1			
	TARS2			
	TARS3			
	TAS2R14			
	TASOR			
	TASOR2			
	TASP1			
	TATDN2			
	TAX1BP1			
	TAZ			
	TBC1D10B			
	TBC1D15			
	TBC1D16			
	TBC1D17			
	TBC1D2			
	TBC1D20			
	TBC1D22A			
	TBC1D25			
	TBC1D2B			
	TBC1D9			
	TBC1D9B			
	TBCD			
	TBCEL			
	TBKBP1			
	TBL3			
	TBP			
	TBPL1			
	TBRG1			
	TBRG4			
	TBX2			
	TBX3			
	TCAF1			

	TCEA3			
	TCEAL4			
	TCEAL8			
	TCEAL9			
	TCEANC2			
	TCERG1			
	TCF12			
	TCF21			
	TCF25			
	TCF3			
	TCF7L1			
	TCF7L2			
	TCFL5			
	TCN1			
	TCN2			
	TCP1			
	TCTN1			
	TCTN3			
	TDP2			
	TDRD1			
	TDRD7			
	TDRKH-AS1			
	TDRP			
	TEAD2			
	TEAD3			
	TEAD4			
	TECPR1			
	TECRP1			
	TECTA			
	TEDC2			
	TEF			
	TELO2			
	TENT4A			
	TENT4B			
	TENT5B			
	TENT5C			
	TERF2			
	TERF2IP			
	TESK1			
	TET2			
	TEX10			
	TEX2			
	TEX264			
	TEX41			
	TEX9			
	TFAP2A			
	TFB2M			
	TFE3			
	TFEB			
	TFEC			
	TFIP11			
	TFPI2			
	TG			
	TGFA			
	TGFB1			
	TGFB1I1			
	TGFB2			
	TGFBR1			
	TGFBR2			
	TGFBRAP1			

	TGIF2			
	TGM2			
	THAP1			
	THAP12			
	THAP2			
	THAP3			
	THAP4			
	THAP5			
	THAP7			
	THAP9-AS1			
	THBD			
	THBS3			
	THEM4			
	THEMIS2			
	THG1L			
	THOC1			
	THOC3			
	THOC6			
	THOC7			
	THOP1			
	THRA			
	THRB			
	THSD4			
	THUMPD1			
	THUMPD3			
	TIA1			
	TIAM1			
	TICAM1			
	TIFA			
	TIGAR			
	TIMELESS			
	TIMM10			
	TIMM10B			
	TIMM13			
	TIMM17B			
	TIMM23			
	TIMM44			
	TIMM9			
	TIMP1			
	TIMP3			
	TINAGL1			
	TINCR			
	TIPRL			
	TJAP1			
	TJP1			
	TJP2			
	TJP3			
	TKFC			
	TKT			
	TLCD3A			
	TLCD4			
	TLCD5			
	TLDC2			
	TLE1			
	TLE2			
	TLE3			
	TLE4			
	TLL1			
	TLN1			
	TLR1			

	TLR2			
	TLR4			
	TLR6			
	TLR7			
	TM4SF1			
	TM4SF19			
	TM4SF20			
	TM7SF3			
	TM9SF3			
	TMA7			
	TMBIM1			
	TMC6			
	TMCO1			
	TMCO3			
	TMCO4			
	TMED10			
	TMED4			
	TMED5			
	TMED8			
	TMED9			
	TMEM100			
	TMEM109			
	TMEM11			
	TMEM115			
	TMEM123			
	TMEM125			
	TMEM126B			
	TMEM127			
	TMEM131			
	TMEM133			
	TMEM134			
	TMEM141			
	TMEM14B			
	TMEM150A			
	TMEM154			
	TMEM159			
	TMEM160			
	TMEM161A			
	TMEM171			
	TMEM175			
	TMEM176B			
	TMEM18			
	TMEM183A			
	TMEM184B			
	TMEM185A			
	TMEM189			
	TMEM192			
	TMEM198B			
	TMEM201			
	TMEM203			
	TMEM205			
	TMEM208			
	TMEM209			
	TMEM212			
	TMEM214			
	TMEM218			
	TMEM219			
	TMEM222			
	TMEM230			
	TMEM243			

	TMEM245			
	TMEM254			
	TMEM254-AS1			
	TMEM255B			
	TMEM258			
	TMEM259			
	TMEM260			
	TMEM39A			
	TMEM39B			
	TMEM41B			
	TMEM43			
	TMEM45A			
	TMEM45B			
	TMEM50A			
	TMEM50B			
	TMEM51			
	TMEM52B			
	TMEM54			
	TMEM59			
	TMEM60			
	TMEM69			
	TMEM71			
	TMEM74B			
	TMEM87B			
	TMEM88			
	TMEM92			
	TMEM94			
	TMEM97			
	TMEM9B			
	TMLHE-AS1			
	TMOD1			
	TMOD2			
	TMOD3			
	TMPRSS2			
	TMSB10			
	TMSB4XP4			
	TMTC1			
	TMUB2			
	TMX1			
	TMX2			
	TNF			
	TNFAIP1			
	TNFAIP6			
	TNFAIP8			
	TNFAIP8L3			
	TNFRSF10B			
	TNFRSF10D			
	TNFRSF14			
	TNFRSF1B			
	TNFRSF21			
	TNFSF10			
	TNFSF13B			
	TNFSF14			
	TNFSF8			
	TNIK			
	TNIP1			
	TNIP2			
	TNIP3			
	TNNC1			
	TNNT2			

	TNPO2			
	TNRC18P3			
	TNRC6C			
	TNS1			
	TNS2			
	TNS4			
	TNXA			
	TNXB			
	TOB2			
	TOE1			
	TOLLIP			
	TOM1			
	TOM1L2			
	TOMM34			
	TOMM40			
	TOMM40L			
	TOP1MT			
	TOP2A			
	TOP2B			
	TOP3A			
	TOR1A			
	TOR1AIP1			
	TOR1AIP2			
	TOR1B			
	TOR4A			
	TOX4			
	TP53			
	TP53BP1			
	TP53BP2			
	TP53I3			
	TP53INP2			
	TP53RK			
	TP53TG3E			
	TP73			
	TP73-AS1			
	TPBG			
	TPCN1			
	TPD52L2			
	TPH2			
	TPM1			
	TPM2			
	TPM4			
	TPMT			
	TPP1			
	TPPP			
	TPR			
	TPRA1			
	TPRG1L			
	TPRKB			
	TPSAB1			
	TPSB2			
	TPST1			
	TPT1			
	TPT1-AS1			
	TPTEP1			
	TRA2B			
	TRABD			
	TRAF1			
	TRAF2			
	TRAF3IP2			

	TRAF3IP2-AS1			
	TRAF4			
	TRAF5			
	TRAF6			
	TRAK1			
	TRAK2			
	TRAM2			
	TRAP1			
	TRAPPC1			
	TRAPPC10			
	TRAPPC12			
	TRAPPC2B			
	TRAPPC3			
	TRAPPC4			
	TRAPPC9			
	TREM1			
	TREML3P			
	TRGC2			
	TRIB1			
	TRIB3			
	TRIM11			
	TRIM14			
	TRIM16			
	TRIM16L			
	TRIM21			
	TRIM22			
	TRIM25			
	TRIM26			
	TRIM27			
	TRIM29			
	TRIM3			
	TRIM38			
	TRIM5			
	TRIM52-AS1			
	TRIM59			
	TRIM68			
	TRIM69			
	TRIO			
	TRIP10			
	TRIP6			
	TRIQK			
	TRIR			
	TRIT1			
	TRMT1			
	TRMT10A			
	TRMT11			
	TRMT12			
	TRMT1L			
	TRMT5			
	TRMT61A			
	TRMU			
	TRNAU1AP			
	TROAP			
	TRPA1			
	TRPC4AP			
	TRPC6			
	TRPM2-AS			
	TRPM4			
	TRPM7			
	TRPM8			

	TRPS1			
	TRRAP			
	TRUB2			
	TSBP1-AS1			
	TSC1			
	TSC2			
	TSC22D1			
	TSC22D3			
	TSC22D4			
	TSEN34			
	TSEN54			
	TSFM			
	TSHZ1			
	TSHZ3			
	TSIX			
	TSN			
	TSPAN14			
	TSPAN17			
	TSPAN18			
	TSPAN31			
	TSPAN4			
	TSPAN7			
	TSPAN9			
	TSPO			
	TSPY26P			
	TSPYL1			
	TSPYL2			
	TSPYL4			
	TSR1			
	TSR3			
	TSSC4			
	TSSK6			
	TSTA3			
	TTBK2			
	TTC14			
	TTC19			
	TTC3			
	TTC31			
	TTC38			
	TTC39B			
	TTC7A			
	TTC7B			
	TTC9C			
	TTF1			
	TTL			
	TTLL4			
	TTPA			
	TTTY15			
	TUBA1A			
	TUBA1B			
	TUBA1C			
	TUBA3D			
	TUBA4A			
	TUBAP2			
	TUBB			
	TUBB2A			
	TUBB2B			
	TUBB4B			
	TUBB6			
	TUBG1			

	TUBGCP2			
	TUBGCP3			
	TUBGCP4			
	TUBGCP6			
	TUFM			
	TUG1			
	TULP3			
	TULP4			
	TUSC2			
	TUSC3			
	TUT4			
	TVP23B			
	TWF1P1			
	TWF2			
	TWIST2			
	TWNK			
	TXLNA			
	TXLNGY			
	TXNDC11			
	TXNDC12			
	TXNDC15			
	TXNL4A			
	TXNL4B			
	TYMS			
	TYRO3			
	TYW1B			
	U2AF1			
	U2AF1L5			
	U2AF2			
	U2SURP			
	U62317.1			
	UACA			
	UAP1			
	UAP1L1			
	UBA1			
	UBA2			
	UBA52			
	UBA7			
	UBAC2			
	UBAC2-AS1			
	UBALD1			
	UBAP2			
	UBASH3B			
	UBB			
	UBC			
	UBE2A			
	UBE2B			
	UBE2D1			
	UBE2D2			
	UBE2D3			
	UBE2E1			
	UBE2E2			
	UBE2E3			
	UBE2F			
	UBE2G2			
	UBE2H			
	UBE2I			
	UBE2J1			
	UBE2J2			
	UBE2L6			

	UBE2M			
	UBE2N			
	UBE2O			
	UBE2Q1			
	UBE2Z			
	UBE3A			
	UBE3C			
	UBIAD1			
	UBL3			
	UBL4A			
	UBL5			
	UBL7			
	UBN1			
	UBQLN1			
	UBQLN4			
	UBR4			
	UBR5			
	UBTF			
	UBXN1			
	UBXN11			
	UBXN2B			
	UBXN6			
	UBXN7			
	UCHL1			
	UCHL5			
	UCK1			
	UCK2			
	UCP2			
	UEVLD			
	UFD1			
	UFSP2			
	UGDH-AS1			
	UGGT2			
	UGT1A7			
	UHRF1BP1			
	UHRF2			
	UIMC1			
	ULBP2			
	ULK1			
	UMPS			
	UNC13A			
	UNC13B			
	UNC13D			
	UNC45A			
	UNC5D			
	UNC93B1			
	UNK			
	UPF1			
	UPF3A			
	UPF3B			
	UPK2			
	UPK3B			
	UPP1			
	UQCC1			
	UQCC2			
	UQCRC1			
	UQCRC2			
	UQCRFS1			
	URB1			
	URB2			

	URGCP			
	URM1			
	UROD			
	USB1			
	USE1			
	USF1			
	USF3			
	USH1C			
	USHBP1			
	USP10			
	USP15			
	USP18			
	USP19			
	USP20			
	USP21			
	USP22			
	USP25			
	USP3			
	USP30			
	USP39			
	USP4			
	USP40			
	USP47			
	USP48			
	USP49			
	USP5			
	USP53			
	USP7			
	UTP14C			
	UTP20			
	UTP23			
	UTP3			
	UTP4			
	UTP6			
	VAC14			
	VAMP1			
	VAMP3			
	VAMP5			
	VANGL1			
	VANGL2			
	VAPB			
	VASH1			
	VASN			
	VASP			
	VAT1			
	VAV1			
	VAV2			
	VAV3			
	VAX1			
	VCAN			
	VCP			
	VCPIP1			
	VCPKMT			
	VDAC1			
	VDAC3			
	VEGFA			
	VEGFB			
	VEGFD			
	VEZT			
	VGLL3			

	VGLL4			
	VIM			
	VIPAS39			
	VIPR1			
	VIRMA			
	VKORC1			
	VLDLR			
	VNN2			
	VNN3			
	VOPP1			
	VPS11			
	VPS13D			
	VPS16			
	VPS18			
	VPS25			
	VPS26A			
	VPS26C			
	VPS28			
	VPS29			
	VPS33B			
	VPS35			
	VPS37B			
	VPS39			
	VPS45			
	VPS51			
	VPS53			
	VPS54			
	VPS72			
	VPS8			
	VSIG1			
	VSIG10			
	VSIG10L			
	VSIG2			
	VSX1			
	VTI1A			
	VTRNA1-1			
	VTRNA1-2			
	VWA7			
	VWF			
	WAPL			
	WARS2			
	WAS			
	WASF2			
	WASF3			
	WASH2P			
	WASH3P			
	WASH4P			
	WASH5P			
	WASH6P			
	WASH7P			
	WASH8P			
	WASH9P			
	WASHC1			
	WASHC2A			
	WASHC4			
	WASL			
	WBP1L			
	WDFY2			
	WDFY3			
	WDR11			

	WDR13			
	WDR18			
	WDR20			
	WDR27			
	WDR34			
	WDR35			
	WDR36			
	WDR37			
	WDR4			
	WDR43			
	WDR44			
	WDR45B			
	WDR46			
	WDR5			
	WDR59			
	WDR6			
	WDR60			
	WDR61			
	WDR74			
	WDR75			
	WDR78			
	WDR82			
	WDR83OS			
	WDR88			
	WDR89			
	WDR91			
	WDTC1			
	WDYHV1			
	WEE1			
	WFDC2			
	WFDC21P			
	WFS1			
	WHAMM			
	WIPF1			
	WIPI1			
	WIPI2			
	WNK4			
	WNT2B			
	WNT7B			
	WNT9A			
	WRAP73			
	WRNIP1			
	WTAP			
	WTIP			
	WWC2			
	WWTR1			
	XAB2			
	XAF1			
	XAGE1A			
	XAGE1B			
	XKR9			
	XPA			
	XPC			
	XPNPEP1			
	XPO5			
	XRCC6			
	XRN1			
	XRN2			
	XYLB			
	XYLT1			

	XYLT2			
	YAE1			
	YAP1			
	YARS1			
	YBX1			
	YBX1P1			
	YEATS2			
	YIPF1			
	YIPF5			
	YIPF6			
	YJU2			
	YKT6			
	YPEL2			
	YPEL3			
	YRDC			
	YTHDF1			
	YWHAE			
	YWHAZ			
	YY2			
	ZBED2			
	ZBED4			
	ZBED5			
	ZBED8			
	ZBP1			
	ZBTB1			
	ZBTB10			
	ZBTB14			
	ZBTB17			
	ZBTB18			
	ZBTB2			
	ZBTB24			
	ZBTB33			
	ZBTB34			
	ZBTB38			
	ZBTB39			
	ZBTB4			
	ZBTB40			
	ZBTB44			
	ZBTB46			
	ZBTB5			
	ZBTB7A			
	ZBTB8OS			
	ZBTB9			
	ZC3H11A			
	ZC3H12A			
	ZC3H18			
	ZC3H4			
	ZC3H6			
	ZC3H7A			
	ZC3H7B			
	ZC3HAV1			
	ZC3HC1			
	ZCCHC14			
	ZCCHC24			
	ZCCHC3			
	ZCCHC7			
	ZCCHC8			
	ZCCHC9			
	ZDHHC11			
	ZDHHC11B			

	ZDHHC12			
	ZDHHC13			
	ZDHHC14			
	ZDHHC16			
	ZDHHC19			
	ZDHHC2			
	ZDHHC6			
	ZDHHC7			
	ZDHHC8P1			
	ZEB1			
	ZER1			
	ZFAND2A			
	ZFAND2B			
	ZFAND5			
	ZFAND6			
	ZFAT			
	ZFP28			
	ZFP3			
	ZFP36L1			
	ZFP36L2			
	ZFP64			
	ZFP90			
	ZFYVE1			
	ZFYVE19			
	ZFYVE27			
	ZHX3			
	ZKSCAN1			
	ZKSCAN5			
	ZMAT2			
	ZMIZ1			
	ZMIZ2			
	ZMPSTE24			
	ZMYM2			
	ZMYM4			
	ZMYND12			
	ZMYND19			
	ZNF10			
	ZNF100			
	ZNF106			
	ZNF12			
	ZNF121			
	ZNF131			
	ZNF133			
	ZNF134			
	ZNF136			
	ZNF140			
	ZNF142			
	ZNF154			
	ZNF160			
	ZNF175			
	ZNF184			
	ZNF185			
	ZNF197			
	ZNF211			
	ZNF212			
	ZNF22			
	ZNF221			
	ZNF222			
	ZNF227			
	ZNF24			

	ZNF248			
	ZNF25			
	ZNF26			
	ZNF263			
	ZNF266			
	ZNF273			
	ZNF276			
	ZNF277			
	ZNF282			
	ZNF286A			
	ZNF3			
	ZNF317			
	ZNF330			
	ZNF331			
	ZNF333			
	ZNF335			
	ZNF343			
	ZNF35			
	ZNF354A			
	ZNF384			
	ZNF385A			
	ZNF385D			
	ZNF394			
	ZNF395			
	ZNF407			
	ZNF408			
	ZNF431			
	ZNF433			
	ZNF440			
	ZNF449			
	ZNF45			
	ZNF451			
	ZNF461			
	ZNF462			
	ZNF468			
	ZNF473			
	ZNF480			
	ZNF488			
	ZNF496			
	ZNF497			
	ZNF502			
	ZNF513			
	ZNF514			
	ZNF518A			
	ZNF558			
	ZNF576			
	ZNF577			
	ZNF583			
	ZNF585B			
	ZNF589			
	ZNF592			
	ZNF595			
	ZNF597			
	ZNF598			
	ZNF599			
	ZNF600			
	ZNF610			
	ZNF614			
	ZNF621			
	ZNF622			

	ZNF629			
	ZNF638			
	ZNF655			
	ZNF664			
	ZNF672			
	ZNF687			
	ZNF688			
	ZNF689			
	ZNF692			
	ZNF697			
	ZNF700			
	ZNF710			
	ZNF720			
	ZNF732			
	ZNF740			
	ZNF76			
	ZNF761			
	ZNF766			
	ZNF768			
	ZNF776			
	ZNF778			
	ZNF783			
	ZNF785			
	ZNF800			
	ZNF81			
	ZNF816			
	ZNF823			
	ZNF829			
	ZNF83			
	ZNF836			
	ZNF84			
	ZNF841			
	ZNF862			
	ZNF876P			
	ZNHIT1			
	ZNHIT3			
	ZNNT1			
	ZNRD2			
	ZNRF2			
	ZP3			
	ZPLD1			
	ZPR1			
	ZRANB1			
	ZRANB3			
	ZRSR2			
	ZSCAN16			
	ZSCAN18			
	ZSCAN22			
	ZSCAN29			
	ZSCAN31			
	ZSCAN9			
	ZSWIM7			
	ZSWIM8			
	ZUP1			
	ZW10			
	ZWINT			
	ZYG11A			
	ZZEF1			
