# Supplementary material for: Transcriptome of nasopharyngeal samples from COVID-19 patients and a comparative analysis with other SARS-CoV-2 infection models reveal disparate host responses against SARS-CoV-2
Source: J Transl Med. 2021 Jan 7;19:32. doi: 10.1186/s12967-020-02695-0 (PMC7790360; doi:10.1186/s12967-020-02695-0)
Supplement: Supplementary file 8 — Additional file 8. Genes and associated terms used for filtering the expression values used in Fig. 6. [file 12967_2020_2695_MOESM8_ESM.pdf]

Additional file 8 : Genes and associated terms used for filtering the expression values used in Figure 6.

Cytokine related genes	Associated terms
CD55	CD4-positive, alpha-beta T cell cytokine production
E2F8	cell cycle comprising mitosis without cytokinesis
AKAP6	cellular response to cytokine stimulus
ASAH2	cellular response to cytokine stimulus
CASP1	cellular response to cytokine stimulus
CCR7	cellular response to cytokine stimulus
CRNN	cellular response to cytokine stimulus
CSF1R	cellular response to cytokine stimulus
CSF3	cellular response to cytokine stimulus
CXCR4	cellular response to cytokine stimulus
FLT3	cellular response to cytokine stimulus
FOXF1	cellular response to cytokine stimulus
HAX1	cellular response to cytokine stimulus
HCLS1	cellular response to cytokine stimulus
IL13	cellular response to cytokine stimulus
IL18BP	cellular response to cytokine stimulus
IL18R1	cellular response to cytokine stimulus
IL18RAP	cellular response to cytokine stimulus
IL1RL2	cellular response to cytokine stimulus
IL37	cellular response to cytokine stimulus
ITGA4	cellular response to cytokine stimulus
LEF1	cellular response to cytokine stimulus
PID1	cellular response to cytokine stimulus
PTPN14	cellular response to cytokine stimulus
PTPN2	cellular response to cytokine stimulus
PTPN7	cellular response to cytokine stimulus
STAT1	cellular response to cytokine stimulus
TRPV1	cellular response to cytokine stimulus
CD28	cytokine biosynthetic process
CEBPE	cytokine biosynthetic process
BATF	cytokine-mediated signaling pathway
BCL6	cytokine-mediated signaling pathway
BIRC5	cytokine-mediated signaling pathway
CASP1	cytokine-mediated signaling pathway
CCL11	cytokine-mediated signaling pathway
CCL20	cytokine-mediated signaling pathway
CCL22	cytokine-mediated signaling pathway
CCL2	cytokine-mediated signaling pathway
CCL4	cytokine-mediated signaling pathway
CCL5	cytokine-mediated signaling pathway
CCR1	cytokine-mediated signaling pathway
CCR2	cytokine-mediated signaling pathway
CCR5	cytokine-mediated signaling pathway
CD36	cytokine-mediated signaling pathway
CD4	cytokine-mediated signaling pathway

CD80	cytokine-mediated signaling pathway
CD86	cytokine-mediated signaling pathway
CNOT9	cytokine-mediated signaling pathway
CNTF	cytokine-mediated signaling pathway
CRK	cytokine-mediated signaling pathway
CSF1	cytokine-mediated signaling pathway
CSF1R	cytokine-mediated signaling pathway
CSF2RB	cytokine-mediated signaling pathway
CSF3	cytokine-mediated signaling pathway
CX3CL1	cytokine-mediated signaling pathway
CXCL10	cytokine-mediated signaling pathway
CXCL1	cytokine-mediated signaling pathway
CXCL2	cytokine-mediated signaling pathway
CXCL8	cytokine-mediated signaling pathway
DUOX1	cytokine-mediated signaling pathway
DUOX2	cytokine-mediated signaling pathway
EPOR	cytokine-mediated signaling pathway
F3	cytokine-mediated signaling pathway
FASLG	cytokine-mediated signaling pathway
FER	cytokine-mediated signaling pathway
FGF2	cytokine-mediated signaling pathway
FLT3	cytokine-mediated signaling pathway
FLT3LG	cytokine-mediated signaling pathway
FN1	cytokine-mediated signaling pathway
FOS	cytokine-mediated signaling pathway
FYN	cytokine-mediated signaling pathway
FZD4	cytokine-mediated signaling pathway
GREM2	cytokine-mediated signaling pathway
HGF	cytokine-mediated signaling pathway
ICAM1	cytokine-mediated signaling pathway
IFNL1	cytokine-mediated signaling pathway
IFNL2	cytokine-mediated signaling pathway
IFNL3	cytokine-mediated signaling pathway
IFNLR1	cytokine-mediated signaling pathway
IGHE	cytokine-mediated signaling pathway
IGHG1	cytokine-mediated signaling pathway
IL11	cytokine-mediated signaling pathway
IL11RA	cytokine-mediated signaling pathway
IL12A	cytokine-mediated signaling pathway
IL13	cytokine-mediated signaling pathway
IL16	cytokine-mediated signaling pathway
IL17RB	cytokine-mediated signaling pathway
IL1A	cytokine-mediated signaling pathway
IL1B	cytokine-mediated signaling pathway
IL1R2	cytokine-mediated signaling pathway
IL1RAP	cytokine-mediated signaling pathway
IL1RAPL2	cytokine-mediated signaling pathway
IL1RL1	cytokine-mediated signaling pathway
IL1RL2	cytokine-mediated signaling pathway
IL1RN	cytokine-mediated signaling pathway

IL20	cytokine-mediated signaling pathway
IL20RB	cytokine-mediated signaling pathway
IL22RA1	cytokine-mediated signaling pathway
IL23A	cytokine-mediated signaling pathway
IL23R	cytokine-mediated signaling pathway
IL24	cytokine-mediated signaling pathway
IL2RA	cytokine-mediated signaling pathway
IL2RG	cytokine-mediated signaling pathway
IL31RA	cytokine-mediated signaling pathway
IL32	cytokine-mediated signaling pathway
IL34	cytokine-mediated signaling pathway
IL36RN	cytokine-mediated signaling pathway
IL37	cytokine-mediated signaling pathway
IL3RA	cytokine-mediated signaling pathway
IL4	cytokine-mediated signaling pathway
IL4R	cytokine-mediated signaling pathway
IL5RA	cytokine-mediated signaling pathway
IL6	cytokine-mediated signaling pathway
IL6R	cytokine-mediated signaling pathway
IL6ST	cytokine-mediated signaling pathway
INPP5D	cytokine-mediated signaling pathway
IRF5	cytokine-mediated signaling pathway
ITGAM	cytokine-mediated signaling pathway
ITGAX	cytokine-mediated signaling pathway
ITGB2	cytokine-mediated signaling pathway
JAK2	cytokine-mediated signaling pathway
JUNB	cytokine-mediated signaling pathway
KRAS	cytokine-mediated signaling pathway
LBP	cytokine-mediated signaling pathway
LRP8	cytokine-mediated signaling pathway
MCL1	cytokine-mediated signaling pathway
MPL	cytokine-mediated signaling pathway
MYC	cytokine-mediated signaling pathway
MYD88	cytokine-mediated signaling pathway
OPRD1	cytokine-mediated signaling pathway
OPRM1	cytokine-mediated signaling pathway
PIK3CB	cytokine-mediated signaling pathway
POMC	cytokine-mediated signaling pathway
PTGS2	cytokine-mediated signaling pathway
PTPRN	cytokine-mediated signaling pathway
SAA1	cytokine-mediated signaling pathway
SOCS1	cytokine-mediated signaling pathway
SOCS3	cytokine-mediated signaling pathway
STAT1	cytokine-mediated signaling pathway
STAT2	cytokine-mediated signaling pathway
STAT4	cytokine-mediated signaling pathway
STAT5B	cytokine-mediated signaling pathway
STX4	cytokine-mediated signaling pathway
TNF	cytokine-mediated signaling pathway
TNFRSF1A	cytokine-mediated signaling pathway

TP53	cytokine-mediated signaling pathway
VCAM1	cytokine-mediated signaling pathway
YWHAZ	cytokine-mediated signaling pathway
TNFSF15	cytokine metabolic process
AZI2	cytokine production
BATF	cytokine production
CD226	cytokine production
CD4	cytokine production
DBH	cytokine production
FABP4	cytokine production
FOXP3	cytokine production
MAF	cytokine production
NFATC1	cytokine production
NFATC2	cytokine production
NFATC3	cytokine production
PIK3CG	cytokine production
S1PR3	cytokine production
CD96	cytokine production involved in inflammatory response
IDO1	cytokine production involved in inflammatory response
BTN3A1	cytokine secretion
NLRP3	cytokine secretion involved in immune response
CECR2	cytoskeleton-dependent cytokinesis
SEPTIN12	cytoskeleton-dependent cytokinesis
SEPTIN1	cytoskeleton-dependent cytokinesis
SEPTIN4	cytoskeleton-dependent cytokinesis
SEPTIN5	cytoskeleton-dependent cytokinesis
SEPTIN6	cytoskeleton-dependent cytokinesis
SEPTIN9	cytoskeleton-dependent cytokinesis
ANK3	mitotic cytokinesis
ANLN	mitotic cytokinesis
APC	mitotic cytokinesis
CENPA	mitotic cytokinesis
CEP55	mitotic cytokinesis
CKAP2	mitotic cytokinesis
ECT2	mitotic cytokinesis
EFHC1	mitotic cytokinesis
ESPL1	mitotic cytokinesis
KIF20A	mitotic cytokinesis
KIF23	mitotic cytokinesis
KIF4A	mitotic cytokinesis
PLK1	mitotic cytokinesis
RACGAP1	mitotic cytokinesis
RHOB	mitotic cytokinesis
ROCK1	mitotic cytokinesis
ROCK2	mitotic cytokinesis
SEPTIN6	mitotic cytokinesis
SPTBN1	mitotic cytokinesis
TRIM36	mitotic cytokinesis
UNC119	mitotic cytokinesis
AURKB	mitotic cytokinesis checkpoint

CNTROB	mitotic cytokinetic process
ASB1	negative regulation of cytokine biosynthetic process
FOXP3	negative regulation of cytokine biosynthetic process
TIA1	negative regulation of cytokine biosynthetic process
IL36RN	negative regulation of cytokine-mediated signaling pathway
PTPRC	negative regulation of cytokine-mediated signaling pathway
PXDN	negative regulation of cytokine-mediated signaling pathway
AXL	negative regulation of cytokine production
BTK	negative regulation of cytokine production
CLEC4A	negative regulation of cytokine production
MIR155	negative regulation of cytokine production
NFKB1	negative regulation of cytokine production
TWSG1	negative regulation of cytokine production
ABCD2	negative regulation of cytokine production involved in inflammatory response
ADCY7	negative regulation of cytokine production involved in inflammatory response
APOD	negative regulation of cytokine production involved in inflammatory response
F2	negative regulation of cytokine production involved in inflammatory response
IL1R2	negative regulation of cytokine production involved in inflammatory response
MEFV	negative regulation of cytokine production involved in inflammatory response
MIR155	negative regulation of cytokine production involved in inflammatory response
ZC3H12A	negative regulation of cytokine production involved in inflammatory response
BTN2A2	negative regulation of cytokine secretion
FCGR2B	negative regulation of cytokine secretion
FFAR4	negative regulation of cytokine secretion
FOXP3	negative regulation of cytokine secretion
PTGER4	negative regulation of cytokine secretion
SRGN	negative regulation of cytokine secretion
ANGPT1	negative regulation of cytokine secretion involved in immune response
APOA1	negative regulation of cytokine secretion involved in immune response
APOA2	negative regulation of cytokine secretion involved in immune response
LILRB1	negative regulation of cytokine secretion involved in immune response
TNF	negative regulation of cytokine secretion involved in immune response
AURKB	negative regulation of cytokinesis
E2F7	negative regulation of cytokinesis
E2F8	negative regulation of cytokinesis
TGFB2	negative regulation of macrophage cytokine production
TGFB3	negative regulation of macrophage cytokine production
BCL6	negative regulation of mast cell cytokine production
CD96	negative regulation of natural killer cell cytokine production
HLA-F	negative regulation of natural killer cell cytokine production
BST2	negative regulation of plasmacytoid dendritic cell cytokine production
APOA1	negative regulation of response to cytokine stimulus
KLF4	negative regulation of response to cytokine stimulus
MAPK7	negative regulation of response to cytokine stimulus
FOXP3	negative regulation of T cell cytokine production
HLA-F	negative regulation of T cell cytokine production
SMAD7	negative regulation of T cell cytokine production
ARG1	negative regulation of T-helper 2 cell cytokine production
NRP1	positive regulation of cytokine activity
AXL	positive regulation of cytokine-mediated signaling pathway

CD74	positive regulation of cytokine-mediated signaling pathway
RIPK2	positive regulation of cytokine-mediated signaling pathway
IFI16	positive regulation of cytokine production
IL21	positive regulation of cytokine production
IL33	positive regulation of cytokine production
NFAM1	positive regulation of cytokine production
TLR3	positive regulation of cytokine production
TNF	positive regulation of cytokine production
WNT5A	positive regulation of cytokine production
CD6	positive regulation of cytokine production involved in inflammatory response
CLEC7A	positive regulation of cytokine production involved in inflammatory response
GBP5	positive regulation of cytokine production involved in inflammatory response
KARS1	positive regulation of cytokine production involved in inflammatory response
MIR21	positive regulation of cytokine production involved in inflammatory response
MYD88	positive regulation of cytokine production involved in inflammatory response
TICAM1	positive regulation of cytokine production involved in inflammatory response
TLR4	positive regulation of cytokine production involved in inflammatory response
TLR6	positive regulation of cytokine production involved in inflammatory response
C1QTNF3	positive regulation of cytokine secretion
CADM1	positive regulation of cytokine secretion
FGR	positive regulation of cytokine secretion
IL1A	positive regulation of cytokine secretion
PTGER4	positive regulation of cytokine secretion
SAA1	positive regulation of cytokine secretion
TNF	positive regulation of cytokine secretion
KARS1	positive regulation of cytokine secretion involved in immune response
TNFRSF14	positive regulation of cytokine secretion involved in immune response
WNT5A	positive regulation of cytokine secretion involved in immune response
AURKB	positive regulation of cytokinesis
CDC14A	positive regulation of cytokinesis
CDC25B	positive regulation of cytokinesis
CUL3	positive regulation of cytokinesis
CXCR5	positive regulation of cytokinesis
DRD3	positive regulation of cytokinesis
ECT2	positive regulation of cytokinesis
KIF14	positive regulation of cytokinesis
KIF23	positive regulation of cytokinesis
OR1A2	positive regulation of cytokinesis
RACGAP1	positive regulation of cytokinesis
RXFP3	positive regulation of cytokinesis
CD36	positive regulation of macrophage cytokine production
CD74	positive regulation of macrophage cytokine production
HLA-G	positive regulation of macrophage cytokine production
LILRB1	positive regulation of macrophage cytokine production
SEMA7A	positive regulation of macrophage cytokine production
SPON2	positive regulation of macrophage cytokine production
TLR4	positive regulation of macrophage cytokine production
WNT5A	positive regulation of macrophage cytokine production
FCER1G	positive regulation of mast cell cytokine production
NR4A3	positive regulation of mast cell cytokine production

NUP62	positive regulation of mitotic cytokinetic process
DHX36	positive regulation of myeloid dendritic cell cytokine production
TICAM1	positive regulation of myeloid dendritic cell cytokine production
CD226	positive regulation of natural killer cell cytokine production
CLNK	positive regulation of natural killer cell cytokine production
HLA-E	positive regulation of natural killer cell cytokine production
HLA-F	positive regulation of natural killer cell cytokine production
HLA-G	positive regulation of natural killer cell cytokine production
IFIH1	positive regulation of response to cytokine stimulus
WNT5A	positive regulation of response to cytokine stimulus
B2M	positive regulation of T cell cytokine production
FZD5	positive regulation of T cell cytokine production
SASH3	positive regulation of T cell cytokine production
IL18R1	positive regulation of T-helper 1 cell cytokine production
IL1B	positive regulation of T-helper 1 cell cytokine production
CD81	positive regulation of T-helper 2 cell cytokine production
IL6	positive regulation of T-helper 2 cell cytokine production
NLRP3	positive regulation of T-helper 2 cell cytokine production
RSAD2	positive regulation of T-helper 2 cell cytokine production
BTK	regulation of B cell cytokine production
GREM2	regulation of cytokine activity
IGF2BP2	regulation of cytokine biosynthetic process
MAP2K3	regulation of cytokine biosynthetic process
ELF1	regulation of cytokine-mediated signaling pathway
RUNX1	regulation of cytokine-mediated signaling pathway
BTN2A1	regulation of cytokine production
BTN2A2	regulation of cytokine production
BTN3A1	regulation of cytokine production
BTN3A2	regulation of cytokine production
BTN3A3	regulation of cytokine production
BTNL2	regulation of cytokine production
BTNL3	regulation of cytokine production
BTNL8	regulation of cytokine production
BTNL9	regulation of cytokine production
ELF1	regulation of cytokine production
ICOSLG	regulation of cytokine production
JPH4	regulation of cytokine production
MOG	regulation of cytokine production
TRIL	regulation of cytokine production involved in immune response
PER1	regulation of cytokine production involved in inflammatory response
CCN4	regulation of cytokine secretion
SOCS1	regulation of cytokine secretion
TLR10	regulation of cytokine secretion
TLR6	regulation of cytokine secretion
TLR8	regulation of cytokine secretion
MIR155	regulation of cytokine secretion involved in immune response
AURKA	regulation of cytokinesis
AURKB	regulation of cytokinesis
CCP110	regulation of cytokinesis
FLCN	regulation of cytokinesis

KIF13A	regulation of cytokinesis
KIF20A	regulation of cytokinesis
KLHL13	regulation of cytokinesis
MYO19	regulation of cytokinesis
PLK1	regulation of cytokinesis
PLK2	regulation of cytokinesis
PLK4	regulation of cytokinesis
PRC1	regulation of cytokinesis
RAB11FIP3	regulation of cytokinesis
UVRAG	regulation of cytokinesis
ECT2	regulation of cytokinesis, actomyosin contractile ring assembly
TLR3	regulation of dendritic cell cytokine production
TLR4	regulation of dendritic cell cytokine production
CCR2	regulation of T cell cytokine production
CLC	regulation of T cell cytokine production
ACP5	response to cytokine
AIF1	response to cytokine
ALDH1A2	response to cytokine
AVPR2	response to cytokine
CCL5	response to cytokine
FOSL1	response to cytokine
FOS	response to cytokine
IL6R	response to cytokine
IL6ST	response to cytokine
ITIH4	response to cytokine
JUNB	response to cytokine
JUN	response to cytokine
MAPKAPK3	response to cytokine
MCL1	response to cytokine
NFKB1	response to cytokine
NFKB2	response to cytokine
OXTR	response to cytokine
PLA2G5	response to cytokine
PML	response to cytokine
PTGS2	response to cytokine
RARA	response to cytokine
RELB	response to cytokine
REL	response to cytokine
SRF	response to cytokine
STAT1	response to cytokine
SYNJ1	response to cytokine
TIMP3	response to cytokine
TIMP4	response to cytokine
TYMS	response to cytokine
XCR1	response to cytokine
IL12A	T-helper 1 cell cytokine production
IL18RAP	T-helper 1 cell cytokine production
DENND1B	T-helper 2 cell cytokine production
IL31RA	T-helper 2 cell cytokine production
IL4	T-helper 2 cell cytokine production

**Inflammation related genes**	**Associated terms**
C4B	inflammatory response
F3	activation of plasma proteins involved in acute inflammatory response
APOA2	acute inflammatory response
NUPR1	acute inflammatory response
OGG1	acute inflammatory response
TREM1	acute inflammatory response
CD6	acute inflammatory response to antigenic stimulus
ICAM1	acute inflammatory response to antigenic stimulus
IL31RA	acute inflammatory response to antigenic stimulus
OPRM1	acute inflammatory response to antigenic stimulus
SERPINC1	acute inflammatory response to antigenic stimulus
CCL11	chronic inflammatory response
CXCL13	chronic inflammatory response
THBS1	chronic inflammatory response
TNF	chronic inflammatory response to antigenic stimulus
IL1A	connective tissue replacement involved in inflammatory response wound healing
CD96	cytokine production involved in inflammatory response
IDO1	cytokine production involved in inflammatory response
FASLG	inflammatory cell apoptotic process
ACKR2	inflammatory response
ACOD1	inflammatory response
ADAM8	inflammatory response
ADORA2A	inflammatory response
ADORA3	inflammatory response
AFAP1L2	inflammatory response
AGER	inflammatory response
AGTR1	inflammatory response
AIF1	inflammatory response
AIM2	inflammatory response
AOC3	inflammatory response
APOL3	inflammatory response
AXL	inflammatory response
BCL6	inflammatory response
BMPR1B	inflammatory response
C3AR1	inflammatory response
C3	inflammatory response
C4A	inflammatory response
C4B	inflammatory response
CALCA	inflammatory response
CAMK1D	inflammatory response
CCL11	inflammatory response
CCL14	inflammatory response
CCL15-CCL14	inflammatory response

CCL16	inflammatory response
CCL17	inflammatory response
CCL20	inflammatory response
CCL22	inflammatory response
CCL2	inflammatory response
CCL4	inflammatory response
CCL5	inflammatory response
CCL8	inflammatory response
CCR1	inflammatory response
CCR2	inflammatory response
CCR5	inflammatory response
CCR7	inflammatory response
CD163	inflammatory response
CD180	inflammatory response
CD40	inflammatory response
CD44	inflammatory response
CD5L	inflammatory response
CHI3L1	inflammatory response
CHUK	inflammatory response
CIITA	inflammatory response
CLEC7A	inflammatory response
CMKLR1	inflammatory response
CNR2	inflammatory response
CRP	inflammatory response
CSF1	inflammatory response
CSF1R	inflammatory response
CSRP3	inflammatory response
CX3CL1	inflammatory response
CXCL10	inflammatory response
CXCL11	inflammatory response
CXCL13	inflammatory response
CXCL1	inflammatory response
CXCL2	inflammatory response
CXCL3	inflammatory response
CXCL6	inflammatory response
CXCL8	inflammatory response
CXCL9	inflammatory response
CXCR3	inflammatory response
CXCR4	inflammatory response
CXCR6	inflammatory response
DAB2IP	inflammatory response
FCGR2B	inflammatory response
FOS	inflammatory response
FPR2	inflammatory response
FPR3	inflammatory response
FUT7	inflammatory response
GBP5	inflammatory response
GPER1	inflammatory response
GPR68	inflammatory response
HDAC4	inflammatory response

HMGB2	inflammatory response
HRH1	inflammatory response
HRH4	inflammatory response
HYAL3	inflammatory response
IDO1	inflammatory response
IFI16	inflammatory response
IL13	inflammatory response
IL15	inflammatory response
IL17C	inflammatory response
IL18R1	inflammatory response
IL18RAP	inflammatory response
IL1A	inflammatory response
IL1B	inflammatory response
IL1RAP	inflammatory response
IL1RL2	inflammatory response
IL1RN	inflammatory response
IL23A	inflammatory response
IL23R	inflammatory response
IL2RA	inflammatory response
IL34	inflammatory response
IL36RN	inflammatory response
IL37	inflammatory response
IL6	inflammatory response
IRGM	inflammatory response
ITGB2	inflammatory response
KDM6B	inflammatory response
KNG1	inflammatory response
KRT16	inflammatory response
LGALS9	inflammatory response
LTB4R2	inflammatory response
LTB4R	inflammatory response
LYZ	inflammatory response
MAP2K3	inflammatory response
MEFV	inflammatory response
MMP25	inflammatory response
MS4A2	inflammatory response
MYD88	inflammatory response
NCR3	inflammatory response
NDST1	inflammatory response
NFAM1	inflammatory response
NFATC3	inflammatory response
NFKB1	inflammatory response
NFKB2	inflammatory response
NFKBID	inflammatory response
NLRP1	inflammatory response
NLRP3	inflammatory response
NLRP4	inflammatory response
NMI	inflammatory response
NOD1	inflammatory response
NOX1	inflammatory response

NRROS	inflammatory response
ORM1	inflammatory response
PIK3CG	inflammatory response
PLA2G2D	inflammatory response
PLA2G2E	inflammatory response
PLA2G4C	inflammatory response
POLB	inflammatory response
PPBP	inflammatory response
PROK2	inflammatory response
PTGDR	inflammatory response
PTGER1	inflammatory response
PTGER3	inflammatory response
PTGER4	inflammatory response
PTGS1	inflammatory response
PTGS2	inflammatory response
PTX3	inflammatory response
PXK	inflammatory response
RARRES2	inflammatory response
REG3A	inflammatory response
RELB	inflammatory response
REL	inflammatory response
RIPK2	inflammatory response
S1PR3	inflammatory response
SCG2	inflammatory response
SELE	inflammatory response
SEMA7A	inflammatory response
STAB1	inflammatory response
TACR1	inflammatory response
TBXA2R	inflammatory response
THBS1	inflammatory response
THEMIS2	inflammatory response
TICAM1	inflammatory response
TLR10	inflammatory response
TLR3	inflammatory response
TLR4	inflammatory response
TLR6	inflammatory response
TLR8	inflammatory response
TMIGD3	inflammatory response
TNFAIP3	inflammatory response
TNF	inflammatory response
TNFRSF1A	inflammatory response
TNIP1	inflammatory response
TOLLIP	inflammatory response
TREX1	inflammatory response
TRIL	inflammatory response
TRPV1	inflammatory response
TSPAN2	inflammatory response
XCR1	inflammatory response
ZC3H12A	inflammatory response
CYSLTR1	inflammatory response to antigenic stimulus

HLA-DRB1	inflammatory response to antigenic stimulus
HMGB2	inflammatory response to antigenic stimulus
IL1A	inflammatory response to antigenic stimulus
IL1B	inflammatory response to antigenic stimulus
IL1RN	inflammatory response to antigenic stimulus
IL20RB	inflammatory response to antigenic stimulus
IL2RA	inflammatory response to antigenic stimulus
IL36RN	inflammatory response to antigenic stimulus
IL37	inflammatory response to antigenic stimulus
IL5RA	inflammatory response to antigenic stimulus
KDM6B	inflammatory response to antigenic stimulus
NOTCH1	inflammatory response to antigenic stimulus
RBPJ	inflammatory response to antigenic stimulus
TREX1	inflammatory response to antigenic stimulus
CCR2	inflammatory response to wounding
LBP	leukocyte chemotaxis involved in inflammatory response
SLAMF8	leukocyte chemotaxis involved in inflammatory response
ADAM8	leukocyte migration involved in inflammatory response
CCR6	leukocyte migration involved in inflammatory response
CX3CL1	leukocyte migration involved in inflammatory response
FUT7	leukocyte migration involved in inflammatory response
ITGB2	leukocyte migration involved in inflammatory response
JAM3	leukocyte migration involved in inflammatory response
SELE	leukocyte migration involved in inflammatory response
APCS	negative regulation of acute inflammatory response
NLRP3	negative regulation of acute inflammatory response
PPARG	negative regulation of acute inflammatory response
FCGR2B	negative regulation of acute inflammatory response to antigenic stimulus
CYP19A1	negative regulation of chronic inflammatory response
FOXP3	negative regulation of chronic inflammatory response
TNFAIP3	negative regulation of chronic inflammatory response
AGER	negative regulation of connective tissue replacement involved in inflammatory response wound healing
ABCD2	negative regulation of cytokine production involved in inflammatory response
ADCY7	negative regulation of cytokine production involved in inflammatory response
APOD	negative regulation of cytokine production involved in inflammatory response
F2	negative regulation of cytokine production involved in inflammatory response
IL1R2	negative regulation of cytokine production involved in inflammatory response
MEFV	negative regulation of cytokine production involved in inflammatory response
MIR155	negative regulation of cytokine production involved in inflammatory response
ZC3H12A	negative regulation of cytokine production involved in inflammatory response
ABR	negative regulation of inflammatory response
ACOD1	negative regulation of inflammatory response
ACP5	negative regulation of inflammatory response
ADORA2A	negative regulation of inflammatory response
APOA1	negative regulation of inflammatory response
APOE	negative regulation of inflammatory response
BCR	negative regulation of inflammatory response
C1QTNF3	negative regulation of inflammatory response
CNR2	negative regulation of inflammatory response

CXCL17	negative regulation of inflammatory response
FFAR4	negative regulation of inflammatory response
FOXF1	negative regulation of inflammatory response
FOXP3	negative regulation of inflammatory response
FPR2	negative regulation of inflammatory response
GHRL	negative regulation of inflammatory response
GHSR	negative regulation of inflammatory response
GPER1	negative regulation of inflammatory response
HGF	negative regulation of inflammatory response
IL2RA	negative regulation of inflammatory response
KLF4	negative regulation of inflammatory response
KRT1	negative regulation of inflammatory response
MAPK7	negative regulation of inflammatory response
MEFV	negative regulation of inflammatory response
MIR147B	negative regulation of inflammatory response
MIR155	negative regulation of inflammatory response
MIR205	negative regulation of inflammatory response
MIR223	negative regulation of inflammatory response
MVK	negative regulation of inflammatory response
NFKB1	negative regulation of inflammatory response
NLRP3	negative regulation of inflammatory response
NR1D1	negative regulation of inflammatory response
PBK	negative regulation of inflammatory response
PPARG	negative regulation of inflammatory response
PTGER4	negative regulation of inflammatory response
PTPN2	negative regulation of inflammatory response
SAA1	negative regulation of inflammatory response
SOCS3	negative regulation of inflammatory response
TEK	negative regulation of inflammatory response
TNFAIP3	negative regulation of inflammatory response
TNFRSF1A	negative regulation of inflammatory response
ZFP36	negative regulation of inflammatory response
GPR17	negative regulation of inflammatory response to antigenic stimulus
HLA-DRB1	negative regulation of inflammatory response to antigenic stimulus
SIGLEC10	negative regulation of inflammatory response to wounding
ARG2	negative regulation of macrophage inflammatory protein 1 alpha production
MEFV	negative regulation of macrophage inflammatory protein 1 alpha production
CD200	negative regulation of neuroinflammatory response
CD200R1	negative regulation of neuroinflammatory response
IGF1	negative regulation of neuroinflammatory response
IL4	negative regulation of neuroinflammatory response
MIR195	negative regulation of neuroinflammatory response
NR1D1	negative regulation of neuroinflammatory response
MEFV	negative regulation of NLRP3 inflammasome complex assembly
DUSP10	negative regulation of respiratory burst involved in inflammatory response
SLAMF8	negative regulation of respiratory burst involved in inflammatory response
ADCY1	neuroinflammatory response
ADCY8	neuroinflammatory response
IL4	neuroinflammatory response
TLR4	nitric oxide production involved in inflammatory response

NLRP1	NLRP1 inflammasome complex assembly
NLRP3	NLRP3 inflammasome complex assembly
ADAM8	positive regulation of acute inflammatory response
C2CD4A	positive regulation of acute inflammatory response
CREB3L3	positive regulation of acute inflammatory response
IL6	positive regulation of acute inflammatory response
IL6ST	positive regulation of acute inflammatory response
PIK3CG	positive regulation of acute inflammatory response
IDO1	positive regulation of chronic inflammatory response
TNF	positive regulation of chronic inflammatory response to antigenic stimulus
CD6	positive regulation of cytokine production involved in inflammatory response
CLEC7A	positive regulation of cytokine production involved in inflammatory response
GBP5	positive regulation of cytokine production involved in inflammatory response
KARS1	positive regulation of cytokine production involved in inflammatory response
MIR21	positive regulation of cytokine production involved in inflammatory response
MYD88	positive regulation of cytokine production involved in inflammatory response
TICAM1	positive regulation of cytokine production involved in inflammatory response
TLR4	positive regulation of cytokine production involved in inflammatory response
TLR6	positive regulation of cytokine production involved in inflammatory response
ADAM8	positive regulation of inflammatory response
AGTR1	positive regulation of inflammatory response
CCN4	positive regulation of inflammatory response
CCR2	positive regulation of inflammatory response
CD47	positive regulation of inflammatory response
CX3CL1	positive regulation of inflammatory response
EGFR	positive regulation of inflammatory response
FABP4	positive regulation of inflammatory response
IL15	positive regulation of inflammatory response
IL17RB	positive regulation of inflammatory response
IL1B	positive regulation of inflammatory response
IL1RL1	positive regulation of inflammatory response
IL21	positive regulation of inflammatory response
IL23A	positive regulation of inflammatory response
IL33	positive regulation of inflammatory response
JAK2	positive regulation of inflammatory response
MIR155	positive regulation of inflammatory response
MIR21	positive regulation of inflammatory response
NAPEPLD	positive regulation of inflammatory response
NFKBIA	positive regulation of inflammatory response
PLA2G2A	positive regulation of inflammatory response
PTGER4	positive regulation of inflammatory response
SERPINE1	positive regulation of inflammatory response
SNCA	positive regulation of inflammatory response
STAT5B	positive regulation of inflammatory response
TGM2	positive regulation of inflammatory response
TLR10	positive regulation of inflammatory response
TLR3	positive regulation of inflammatory response
TLR4	positive regulation of inflammatory response
TNF	positive regulation of inflammatory response
TNFRSF1A	positive regulation of inflammatory response

TNIP1	positive regulation of inflammatory response
TRPV4	positive regulation of inflammatory response
WNT5A	positive regulation of inflammatory response
CD28	positive regulation of inflammatory response to antigenic stimulus
CD81	positive regulation of inflammatory response to antigenic stimulus
MIR21	positive regulation of inflammatory response to wounding
SERPINE1	positive regulation of leukotriene production involved in inflammatory response
TRPV4	positive regulation of macrophage inflammatory protein 1 alpha production
IL1B	positive regulation of neuroinflammatory response
IL33	positive regulation of neuroinflammatory response
IL6	positive regulation of neuroinflammatory response
NUPR1	positive regulation of neuroinflammatory response
TNF	positive regulation of neuroinflammatory response
CD36	positive regulation of NLRP3 inflammasome complex assembly
DDX3X	positive regulation of NLRP3 inflammasome complex assembly
GBP5	positive regulation of NLRP3 inflammasome complex assembly
TLR4	positive regulation of NLRP3 inflammasome complex assembly
TLR6	positive regulation of NLRP3 inflammasome complex assembly
LBP	positive regulation of respiratory burst involved in inflammatory response
CD36	production of molecular mediator involved in inflammatory response
IL4R	production of molecular mediator involved in inflammatory response
DNASE1	regulation of acute inflammatory response
CCL5	regulation of chronic inflammatory response
PER1	regulation of cytokine production involved in inflammatory response
ABHD12	regulation of inflammatory response
AGER	regulation of inflammatory response
AGTR1	regulation of inflammatory response
AKNA	regulation of inflammatory response
BCL6B	regulation of inflammatory response
BCL6	regulation of inflammatory response
BIRC3	regulation of inflammatory response
BRD4	regulation of inflammatory response
CASP1	regulation of inflammatory response
CCR2	regulation of inflammatory response
CYLD	regulation of inflammatory response
DUOXA1	regulation of inflammatory response
DUOXA2	regulation of inflammatory response
ESR1	regulation of inflammatory response
FABP4	regulation of inflammatory response
FANCA	regulation of inflammatory response
GGT1	regulation of inflammatory response
IL1RL2	regulation of inflammatory response
IL20	regulation of inflammatory response
JAK2	regulation of inflammatory response
MAS1	regulation of inflammatory response
MCPH1	regulation of inflammatory response
MYD88	regulation of inflammatory response
NLRP1	regulation of inflammatory response
NLRP3	regulation of inflammatory response
PIK3AP1	regulation of inflammatory response

PTGS2	regulation of inflammatory response
RICTOR	regulation of inflammatory response
SBNO2	regulation of inflammatory response
SELE	regulation of inflammatory response
SEMA7A	regulation of inflammatory response
SPATA2	regulation of inflammatory response
STING1	regulation of inflammatory response
TLR4	regulation of inflammatory response
TNF	regulation of inflammatory response
TNIP1	regulation of inflammatory response
TREX1	regulation of inflammatory response
USP18	regulation of inflammatory response
WNT5A	regulation of inflammatory response
CD200R1	regulation of neuroinflammatory response
CD200	regulation of neuroinflammatory response
IL6	regulation of neuroinflammatory response
PTGS2	regulation of neuroinflammatory response
C2CD4A	regulation of vascular permeability involved in acute inflammatory response
MYLK3	regulation of vascular permeability involved in acute inflammatory response

Integrin related genes	Associated terms
CD14	Beta-1 integrin cell surface interactions
CD81	Beta-1 integrin cell surface interactions
COL1A1	Beta-1 integrin cell surface interactions
COL1A2	Beta-1 integrin cell surface interactions
COL2A1	Beta-1 integrin cell surface interactions
COL3A1	Beta-1 integrin cell surface interactions
COL4A1	Beta-1 integrin cell surface interactions
COL4A3	Beta-1 integrin cell surface interactions
COL4A4	Beta-1 integrin cell surface interactions
COL4A5	Beta-1 integrin cell surface interactions
COL4A6	Beta-1 integrin cell surface interactions
COL5A1	Beta-1 integrin cell surface interactions
COL5A2	Beta-1 integrin cell surface interactions
COL6A1	Beta-1 integrin cell surface interactions
COL6A2	Beta-1 integrin cell surface interactions
COL6A3	Beta-1 integrin cell surface interactions
COL7A1	Beta-1 integrin cell surface interactions
COL11A1	Beta-1 integrin cell surface interactions
COL11A2	Beta-1 integrin cell surface interactions
CSPG4	Beta-1 integrin cell surface interactions
F13A1	Beta-1 integrin cell surface interactions
FBN1	Beta-1 integrin cell surface interactions
FGA	Beta-1 integrin cell surface interactions

FGB	Beta-1 integrin cell surface interactions
FGG	Beta-1 integrin cell surface interactions
FN1	Beta-1 integrin cell surface interactions
TNC	Beta-1 integrin cell surface interactions
ITGA6	Beta-1 integrin cell surface interactions
ITGA1	Beta-1 integrin cell surface interactions
ITGA2	Beta-1 integrin cell surface interactions
ITGA3	Beta-1 integrin cell surface interactions
ITGA4	Beta-1 integrin cell surface interactions
ITGA5	Beta-1 integrin cell surface interactions
ITGA7	Beta-1 integrin cell surface interactions
ITGA9	Beta-1 integrin cell surface interactions
ITGAV	Beta-1 integrin cell surface interactions
ITGB1	Beta-1 integrin cell surface interactions
LAMA2	Beta-1 integrin cell surface interactions
LAMA3	Beta-1 integrin cell surface interactions
LAMA4	Beta-1 integrin cell surface interactions
LAMA5	Beta-1 integrin cell surface interactions
LAMB1	Beta-1 integrin cell surface interactions
LAMB2	Beta-1 integrin cell surface interactions
LAMB3	Beta-1 integrin cell surface interactions
LAMC1	Beta-1 integrin cell surface interactions
LAMC2	Beta-1 integrin cell surface interactions
MDK	Beta-1 integrin cell surface interactions
NID1	Beta-1 integrin cell surface interactions
PLAU	Beta-1 integrin cell surface interactions
PLAUR	Beta-1 integrin cell surface interactions
SPP1	Beta-1 integrin cell surface interactions
TGFBI	Beta-1 integrin cell surface interactions
TGM2	Beta-1 integrin cell surface interactions
THBS1	Beta-1 integrin cell surface interactions
THBS2	Beta-1 integrin cell surface interactions
VCAM1	Beta-1 integrin cell surface interactions
VEGFA	Beta-1 integrin cell surface interactions
VTN	Beta-1 integrin cell surface interactions
ITGA10	Beta-1 integrin cell surface interactions
ITGA8	Beta-1 integrin cell surface interactions
ITGA11	Beta-1 integrin cell surface interactions
JAM2	Beta-1 integrin cell surface interactions
COL18A1	Beta-1 integrin cell surface interactions
IGSF8	Beta-1 integrin cell surface interactions
NPNT	Beta-1 integrin cell surface interactions
LAMA1	Beta-1 integrin cell surface interactions
CDH1	Alpha-E beta-7 integrin cell surface interactions
ITGAE	Alpha-E beta-7 integrin cell surface interactions
ITGB7	Alpha-E beta-7 integrin cell surface interactions
CD47	Beta-3 integrin cell surface interactions
COL1A1	Beta-3 integrin cell surface interactions
COL1A2	Beta-3 integrin cell surface interactions
COL4A1	Beta-3 integrin cell surface interactions

COL4A3	Beta-3 integrin cell surface interactions
COL4A4	Beta-3 integrin cell surface interactions
COL4A5	Beta-3 integrin cell surface interactions
COL4A6	Beta-3 integrin cell surface interactions
FBN1	Beta-3 integrin cell surface interactions
FGA	Beta-3 integrin cell surface interactions
FGB	Beta-3 integrin cell surface interactions
FGG	Beta-3 integrin cell surface interactions
FN1	Beta-3 integrin cell surface interactions
HMGB1	Beta-3 integrin cell surface interactions
TNC	Beta-3 integrin cell surface interactions
IBSP	Beta-3 integrin cell surface interactions
CYR61	Beta-3 integrin cell surface interactions
ITGA2B	Beta-3 integrin cell surface interactions
ITGAV	Beta-3 integrin cell surface interactions
ITGB3	Beta-3 integrin cell surface interactions
KDR	Beta-3 integrin cell surface interactions
L1CAM	Beta-3 integrin cell surface interactions
LAMA4	Beta-3 integrin cell surface interactions
LAMB1	Beta-3 integrin cell surface interactions
LAMC1	Beta-3 integrin cell surface interactions
PDGFB	Beta-3 integrin cell surface interactions
PDGFRB	Beta-3 integrin cell surface interactions
PECAM1	Beta-3 integrin cell surface interactions
PLAU	Beta-3 integrin cell surface interactions
PLAUR	Beta-3 integrin cell surface interactions
PVR	Beta-3 integrin cell surface interactions
SDC1	Beta-3 integrin cell surface interactions
SDC4	Beta-3 integrin cell surface interactions
SPP1	Beta-3 integrin cell surface interactions
TGFBI	Beta-3 integrin cell surface interactions
TGFBR2	Beta-3 integrin cell surface interactions
THBS1	Beta-3 integrin cell surface interactions
THY1	Beta-3 integrin cell surface interactions
VEGFA	Beta-3 integrin cell surface interactions
VTN	Beta-3 integrin cell surface interactions
SPHK1	Beta-3 integrin cell surface interactions
EDIL3	Beta-3 integrin cell surface interactions
F11R	Beta-3 integrin cell surface interactions
RHOA	Alpha-V beta-3 integrin/OPN pathway
CD44	Alpha-V beta-3 integrin/OPN pathway
CDC42	Alpha-V beta-3 integrin/OPN pathway
CHUK	Alpha-V beta-3 integrin/OPN pathway
PTK2B	Alpha-V beta-3 integrin/OPN pathway
FOS	Alpha-V beta-3 integrin/OPN pathway
GSN	Alpha-V beta-3 integrin/OPN pathway
ILK	Alpha-V beta-3 integrin/OPN pathway
ITGAV	Alpha-V beta-3 integrin/OPN pathway
ITGB3	Alpha-V beta-3 integrin/OPN pathway
JUN	Alpha-V beta-3 integrin/OPN pathway

MAP3K1	Alpha-V beta-3 integrin/OPN pathway
MMP2	Alpha-V beta-3 integrin/OPN pathway
MMP9	Alpha-V beta-3 integrin/OPN pathway
NFKB1	Alpha-V beta-3 integrin/OPN pathway
NFKBIA	Alpha-V beta-3 integrin/OPN pathway
PIK3CA	Alpha-V beta-3 integrin/OPN pathway
PIK3R1	Alpha-V beta-3 integrin/OPN pathway
PLAU	Alpha-V beta-3 integrin/OPN pathway
MAPK1	Alpha-V beta-3 integrin/OPN pathway
MAPK3	Alpha-V beta-3 integrin/OPN pathway
MAPK8	Alpha-V beta-3 integrin/OPN pathway
RAC1	Alpha-V beta-3 integrin/OPN pathway
RELA	Alpha-V beta-3 integrin/OPN pathway
SPP1	Alpha-V beta-3 integrin/OPN pathway
SYK	Alpha-V beta-3 integrin/OPN pathway
PIP5K1A	Alpha-V beta-3 integrin/OPN pathway
MAP3K14	Alpha-V beta-3 integrin/OPN pathway
ROCK2	Alpha-V beta-3 integrin/OPN pathway
BCAR1	Alpha-V beta-3 integrin/OPN pathway
VAV3	Alpha-V beta-3 integrin/OPN pathway
ADRB2	Arf6 integrin-mediated signaling pathway
AGTR1	Arf6 integrin-mediated signaling pathway
BIN1	Arf6 integrin-mediated signaling pathway
ARF6	Arf6 integrin-mediated signaling pathway
AVPR2	Arf6 integrin-mediated signaling pathway
CDH1	Arf6 integrin-mediated signaling pathway
CLTC	Arf6 integrin-mediated signaling pathway
CPE	Arf6 integrin-mediated signaling pathway
CTNNA1	Arf6 integrin-mediated signaling pathway
CTNNB1	Arf6 integrin-mediated signaling pathway
CTNND1	Arf6 integrin-mediated signaling pathway
DNM2	Arf6 integrin-mediated signaling pathway
EDNRB	Arf6 integrin-mediated signaling pathway
IL2RA	Arf6 integrin-mediated signaling pathway
INS	Arf6 integrin-mediated signaling pathway
ITGA6	Arf6 integrin-mediated signaling pathway
ITGA1	Arf6 integrin-mediated signaling pathway
ITGA2	Arf6 integrin-mediated signaling pathway
ITGA3	Arf6 integrin-mediated signaling pathway
ITGA4	Arf6 integrin-mediated signaling pathway
ITGA5	Arf6 integrin-mediated signaling pathway
ITGA7	Arf6 integrin-mediated signaling pathway
ITGA9	Arf6 integrin-mediated signaling pathway
ITGAV	Arf6 integrin-mediated signaling pathway
ITGB1	Arf6 integrin-mediated signaling pathway
KLC1	Arf6 integrin-mediated signaling pathway
NME1	Arf6 integrin-mediated signaling pathway
PLD1	Arf6 integrin-mediated signaling pathway
PLD2	Arf6 integrin-mediated signaling pathway
RALA	Arf6 integrin-mediated signaling pathway

SLC2A4	Arf6 integrin-mediated signaling pathway
TSHR	Arf6 integrin-mediated signaling pathway
ITGA10	Arf6 integrin-mediated signaling pathway
ITGA8	Arf6 integrin-mediated signaling pathway
ASAP2	Arf6 integrin-mediated signaling pathway
SPAG9	Arf6 integrin-mediated signaling pathway
VAMP3	Arf6 integrin-mediated signaling pathway
ACAP1	Arf6 integrin-mediated signaling pathway
SCAMP2	Arf6 integrin-mediated signaling pathway
EXOC5	Arf6 integrin-mediated signaling pathway
EXOC3	Arf6 integrin-mediated signaling pathway
ITGA11	Arf6 integrin-mediated signaling pathway
MAPK8IP3	Arf6 integrin-mediated signaling pathway
EXOC7	Arf6 integrin-mediated signaling pathway
PIP5K1C	Arf6 integrin-mediated signaling pathway
EXOC6	Arf6 integrin-mediated signaling pathway
EXOC1	Arf6 integrin-mediated signaling pathway
EXOC2	Arf6 integrin-mediated signaling pathway
EXOC4	Arf6 integrin-mediated signaling pathway
ADAM8	Alpha-9 beta-1 integrin pathway
CSF2	Alpha-9 beta-1 integrin pathway
CSF2RA	Alpha-9 beta-1 integrin pathway
F13A1	Alpha-9 beta-1 integrin pathway
FIGF	Alpha-9 beta-1 integrin pathway
FN1	Alpha-9 beta-1 integrin pathway
ADAM2	Alpha-9 beta-1 integrin pathway
TNC	Alpha-9 beta-1 integrin pathway
ITGA9	Alpha-9 beta-1 integrin pathway
ITGB1	Alpha-9 beta-1 integrin pathway
KCNJ15	Alpha-9 beta-1 integrin pathway
NOS2	Alpha-9 beta-1 integrin pathway
PXN	Alpha-9 beta-1 integrin pathway
RAC1	Alpha-9 beta-1 integrin pathway
SAT1	Alpha-9 beta-1 integrin pathway
SPP1	Alpha-9 beta-1 integrin pathway
SRC	Alpha-9 beta-1 integrin pathway
TGM2	Alpha-9 beta-1 integrin pathway
VCAM1	Alpha-9 beta-1 integrin pathway
VEGFA	Alpha-9 beta-1 integrin pathway
VEGFC	Alpha-9 beta-1 integrin pathway
ADAM12	Alpha-9 beta-1 integrin pathway
ADAM15	Alpha-9 beta-1 integrin pathway
BCAR1	Alpha-9 beta-1 integrin pathway
PAOX	Alpha-9 beta-1 integrin pathway
AGER	Alpha-M beta-2 integrin signaling
AKT1	Alpha-M beta-2 integrin signaling
APOB	Alpha-M beta-2 integrin signaling
RHOA	Alpha-M beta-2 integrin signaling
BLK	Alpha-M beta-2 integrin signaling
CTGF	Alpha-M beta-2 integrin signaling

FGR	Alpha-M beta-2 integrin signaling
FYN	Alpha-M beta-2 integrin signaling
HCK	Alpha-M beta-2 integrin signaling
HMGB1	Alpha-M beta-2 integrin signaling
ICAM1	Alpha-M beta-2 integrin signaling
IL6	Alpha-M beta-2 integrin signaling
ITGAM	Alpha-M beta-2 integrin signaling
ITGB2	Alpha-M beta-2 integrin signaling
LCK	Alpha-M beta-2 integrin signaling
LPA	Alpha-M beta-2 integrin signaling
LRP1	Alpha-M beta-2 integrin signaling
LYN	Alpha-M beta-2 integrin signaling
MMP2	Alpha-M beta-2 integrin signaling
MMP9	Alpha-M beta-2 integrin signaling
MST1	Alpha-M beta-2 integrin signaling
MST1R	Alpha-M beta-2 integrin signaling
MYH2	Alpha-M beta-2 integrin signaling
NFKB1	Alpha-M beta-2 integrin signaling
PLAT	Alpha-M beta-2 integrin signaling
PLAU	Alpha-M beta-2 integrin signaling
PLAUR	Alpha-M beta-2 integrin signaling
PLG	Alpha-M beta-2 integrin signaling
PRKCZ	Alpha-M beta-2 integrin signaling
RAP1A	Alpha-M beta-2 integrin signaling
RAP1B	Alpha-M beta-2 integrin signaling
ROCK1	Alpha-M beta-2 integrin signaling
SELP	Alpha-M beta-2 integrin signaling
SELPLG	Alpha-M beta-2 integrin signaling
SRC	Alpha-M beta-2 integrin signaling
THY1	Alpha-M beta-2 integrin signaling
TLN1	Alpha-M beta-2 integrin signaling
TNF	Alpha-M beta-2 integrin signaling
YES1	Alpha-M beta-2 integrin signaling
JAM2	Alpha-M beta-2 integrin signaling
JAM3	Alpha-M beta-2 integrin signaling
RHOA	Alpha-4 beta-7 integrin signaling
CD44	Alpha-4 beta-7 integrin signaling
ITGA4	Alpha-4 beta-7 integrin signaling
ITGB1	Alpha-4 beta-7 integrin signaling
ITGB7	Alpha-4 beta-7 integrin signaling
PTK2	Alpha-4 beta-7 integrin signaling
PXN	Alpha-4 beta-7 integrin signaling
VCAM1	Alpha-4 beta-7 integrin signaling
MADCAM1	Alpha-4 beta-7 integrin signaling
AKT1	Alpha-6 beta-4 integrin signaling pathway
RHOA	Alpha-6 beta-4 integrin signaling pathway
MAPK14	Alpha-6 beta-4 integrin signaling pathway
EIF4EBP1	Alpha-6 beta-4 integrin signaling pathway
MTOR	Alpha-6 beta-4 integrin signaling pathway
GAB1	Alpha-6 beta-4 integrin signaling pathway

HRAS	Alpha-6 beta-4 integrin signaling pathway
ITGA6	Alpha-6 beta-4 integrin signaling pathway
IRS1	Alpha-6 beta-4 integrin signaling pathway
ITGB4	Alpha-6 beta-4 integrin signaling pathway
LAMA2	Alpha-6 beta-4 integrin signaling pathway
LAMA3	Alpha-6 beta-4 integrin signaling pathway
LAMA5	Alpha-6 beta-4 integrin signaling pathway
LAMB1	Alpha-6 beta-4 integrin signaling pathway
LAMB2	Alpha-6 beta-4 integrin signaling pathway
LAMB3	Alpha-6 beta-4 integrin signaling pathway
LAMC1	Alpha-6 beta-4 integrin signaling pathway
LAMC2	Alpha-6 beta-4 integrin signaling pathway
PIK3R1	Alpha-6 beta-4 integrin signaling pathway
PIK3R2	Alpha-6 beta-4 integrin signaling pathway
PRKCA	Alpha-6 beta-4 integrin signaling pathway
PRKCD	Alpha-6 beta-4 integrin signaling pathway
MAPK1	Alpha-6 beta-4 integrin signaling pathway
MAPK3	Alpha-6 beta-4 integrin signaling pathway
PTK2	Alpha-6 beta-4 integrin signaling pathway
PTPN11	Alpha-6 beta-4 integrin signaling pathway
RAC1	Alpha-6 beta-4 integrin signaling pathway
SHC1	Alpha-6 beta-4 integrin signaling pathway
SRC	Alpha-6 beta-4 integrin signaling pathway
IRS2	Alpha-6 beta-4 integrin signaling pathway
LAMA1	Alpha-6 beta-4 integrin signaling pathway
AKT1	Alpha-6 beta-1 and alpha-6 beta-4 integrin signaling
CASP7	Alpha-6 beta-1 and alpha-6 beta-4 integrin signaling
CD9	Alpha-6 beta-1 and alpha-6 beta-4 integrin signaling
CDH1	Alpha-6 beta-1 and alpha-6 beta-4 integrin signaling
COL17A1	Alpha-6 beta-1 and alpha-6 beta-4 integrin signaling
EGF	Alpha-6 beta-1 and alpha-6 beta-4 integrin signaling
EGFR	Alpha-6 beta-1 and alpha-6 beta-4 integrin signaling
ERBB2	Alpha-6 beta-1 and alpha-6 beta-4 integrin signaling
ERBB3	Alpha-6 beta-1 and alpha-6 beta-4 integrin signaling
SFN	Alpha-6 beta-1 and alpha-6 beta-4 integrin signaling
GRB2	Alpha-6 beta-1 and alpha-6 beta-4 integrin signaling
HRAS	Alpha-6 beta-1 and alpha-6 beta-4 integrin signaling
IL1A	Alpha-6 beta-1 and alpha-6 beta-4 integrin signaling
ITGA6	Alpha-6 beta-1 and alpha-6 beta-4 integrin signaling
ITGB1	Alpha-6 beta-1 and alpha-6 beta-4 integrin signaling
ITGB4	Alpha-6 beta-1 and alpha-6 beta-4 integrin signaling
LAMA2	Alpha-6 beta-1 and alpha-6 beta-4 integrin signaling
LAMA3	Alpha-6 beta-1 and alpha-6 beta-4 integrin signaling
LAMA4	Alpha-6 beta-1 and alpha-6 beta-4 integrin signaling
LAMA5	Alpha-6 beta-1 and alpha-6 beta-4 integrin signaling
LAMB1	Alpha-6 beta-1 and alpha-6 beta-4 integrin signaling
LAMB2	Alpha-6 beta-1 and alpha-6 beta-4 integrin signaling
LAMB3	Alpha-6 beta-1 and alpha-6 beta-4 integrin signaling
LAMC1	Alpha-6 beta-1 and alpha-6 beta-4 integrin signaling
LAMC2	Alpha-6 beta-1 and alpha-6 beta-4 integrin signaling

MET	Alpha-6 beta-1 and alpha-6 beta-4 integrin signaling
MST1	Alpha-6 beta-1 and alpha-6 beta-4 integrin signaling
MST1R	Alpha-6 beta-1 and alpha-6 beta-4 integrin signaling
PIK3CA	Alpha-6 beta-1 and alpha-6 beta-4 integrin signaling
PIK3R1	Alpha-6 beta-1 and alpha-6 beta-4 integrin signaling
PMP22	Alpha-6 beta-1 and alpha-6 beta-4 integrin signaling
PRKCA	Alpha-6 beta-1 and alpha-6 beta-4 integrin signaling
RAC1	Alpha-6 beta-1 and alpha-6 beta-4 integrin signaling
RPS6KB1	Alpha-6 beta-1 and alpha-6 beta-4 integrin signaling
RXRA	Alpha-6 beta-1 and alpha-6 beta-4 integrin signaling
RXRB	Alpha-6 beta-1 and alpha-6 beta-4 integrin signaling
RXRG	Alpha-6 beta-1 and alpha-6 beta-4 integrin signaling
SHC1	Alpha-6 beta-1 and alpha-6 beta-4 integrin signaling
YWHAB	Alpha-6 beta-1 and alpha-6 beta-4 integrin signaling
YWHAE	Alpha-6 beta-1 and alpha-6 beta-4 integrin signaling
YWHAG	Alpha-6 beta-1 and alpha-6 beta-4 integrin signaling
YWHAH	Alpha-6 beta-1 and alpha-6 beta-4 integrin signaling
YWHAZ	Alpha-6 beta-1 and alpha-6 beta-4 integrin signaling
LAMC3	Alpha-6 beta-1 and alpha-6 beta-4 integrin signaling
YWHAQ	Alpha-6 beta-1 and alpha-6 beta-4 integrin signaling
LAMA1	Alpha-6 beta-1 and alpha-6 beta-4 integrin signaling
CRK	p130Cas linkage to MAPK signaling for integrins
PTK2	p130Cas linkage to MAPK signaling for integrins
BCAR1	p130Cas linkage to MAPK signaling for integrins
FES	Sema3A-plexin repulsion signaling by inhibiting integrin adhesion
FYN	Sema3A-plexin repulsion signaling by inhibiting integrin adhesion
PLXNA1	Sema3A-plexin repulsion signaling by inhibiting integrin adhesion
PLXNA2	Sema3A-plexin repulsion signaling by inhibiting integrin adhesion
RAC1	Sema3A-plexin repulsion signaling by inhibiting integrin adhesion
RRAS	Sema3A-plexin repulsion signaling by inhibiting integrin adhesion
TLN1	Sema3A-plexin repulsion signaling by inhibiting integrin adhesion
NRP1	Sema3A-plexin repulsion signaling by inhibiting integrin adhesion
FARP2	Sema3A-plexin repulsion signaling by inhibiting integrin adhesion
SEMA3A	Sema3A-plexin repulsion signaling by inhibiting integrin adhesion
PIP5K1C	Sema3A-plexin repulsion signaling by inhibiting integrin adhesion
RND1	Sema3A-plexin repulsion signaling by inhibiting integrin adhesion
PLXNA3	Sema3A-plexin repulsion signaling by inhibiting integrin adhesion
PLXNA4	Sema3A-plexin repulsion signaling by inhibiting integrin adhesion
CRK	GRB2-SOS provides linkage to MAPK signaling for integrins
FGA	GRB2-SOS provides linkage to MAPK signaling for integrins
FGB	GRB2-SOS provides linkage to MAPK signaling for integrins
FGG	GRB2-SOS provides linkage to MAPK signaling for integrins
FN1	GRB2-SOS provides linkage to MAPK signaling for integrins
GRB2	GRB2-SOS provides linkage to MAPK signaling for integrins
ITGA2B	GRB2-SOS provides linkage to MAPK signaling for integrins
ITGB3	GRB2-SOS provides linkage to MAPK signaling for integrins
PTK2	GRB2-SOS provides linkage to MAPK signaling for integrins
RAP1A	GRB2-SOS provides linkage to MAPK signaling for integrins
RAP1B	GRB2-SOS provides linkage to MAPK signaling for integrins
SOS1	GRB2-SOS provides linkage to MAPK signaling for integrins

SRC	GRB2-SOS provides linkage to MAPK signaling for integrins
TLN1	GRB2-SOS provides linkage to MAPK signaling for integrins
VWF	GRB2-SOS provides linkage to MAPK signaling for integrins
BCAR1	GRB2-SOS provides linkage to MAPK signaling for integrins
APBB1IP	GRB2-SOS provides linkage to MAPK signaling for integrins
