# Supplementary material for: Transcriptome of nasopharyngeal samples from COVID-19 patients and a comparative analysis with other SARS-CoV-2 infection models reveal disparate host responses against SARS-CoV-2
Source: J Transl Med. 2021 Jan 7;19:32. doi: 10.1186/s12967-020-02695-0 (PMC7790360; doi:10.1186/s12967-020-02695-0)
Supplement: Supplementary file 10 — Additional file 10. Differentially expressed genes in SARS-CoV-2 infected lungs compared to the our nasal samples used in this study. [file 12967_2020_2695_MOESM10_ESM.pdf]

Additional file 10 : Differentially expressed genes in SARS-CoV-2 infected lungs compared to the our nasal samples used in this study.

Up-regulated in Lungs compared to Nasal samples (Bangladeshi)	Down-regulated in Lungs compared to Nasal samples (Bangladeshi)
CLCA4	AC027290.3
S100A2	IL1RN
FGFBP1	RN7SKP255
AC091429.1	CD53
LYPD3	SPI1
RPL17P36	PNRC1
MTRNR2L1	IFI44L
FOLH1B	AC073571.1
RPL21P134	MX2
RPL41P2	FBXO48
EEF1A1P12	ZFP36
KRT6C	AL354919.1
TPT1P9	IFIT1
KRT8P48	AC016590.1
AC209007.1	H2BC4
AC090543.3	PTCHD4
AC004453.1	IFIT2
EEF1A1P14	DUSP5
UNC93B2	MUC5AC
SLC44A5	MUC5B
H3C9P	AEN
YWHAZP4	AC099489.1
BX248409.1	NR4A1
FTH1P5	SKAP2
MT-TA	AC006435.4
RPL10P4	CROCC2
AL133260.1	H2BC8
KRT8P5	EGR1
RPL31P63	HOPX
AC091685.2	EVI2A
PPIAP13	SCARNA7
EEF1A1P16	C3AR1
AC073072.1	AC092299.7
EEF1A1P22	RN7SKP80
LDHBP2	AC015967.2
EEF1A1P8	FP236383.8
FTH1P12	H4C8
PTMAP5	H2BC7
HSP90AA2P	MEG3
RPS2P7	MOCS1
KRT8P3	HTN3
RPS27AP16	RN7SKP203
PPIAP16	FP236383.7
RPL17P22	FAM27E3
RPS18P12	H2AC12
HSPA8P1	SNORA54
PDIA3P1	SLC38A5

RPL10P6	H19
AC104563.1	SIGLEC14
AC022210.1	MT-RNR1
GAPDHP63	JSRP1
MT-TE	SNORA79B
RPL7P23	VSIG4
FTH1P16	RN7SL396P
APOD	RNU5B-1
PPIAP6	SSTR5-AS1
RPL24P4	IGKC
GAPDHP1	LINC01783
SETP20	AC011595.1
AC104339.1	RNU6ATAC
KRT8P32	COL5A1
MFSD3	RN7SL471P
AC012005.1	SNORD94
BX679664.1	HIST1H3B
AC025518.1	SNORA74D
HLA-J	CCL19
RPL7P15	SNORA71D
EEF1A1P7	H1-5
RPL12P38	SNORD17
CROT	FRZB
RPS3AP5	ADAMTS8
AC068522.1	RN7SKP71
MTND6P3	PLA2G5
AL162430.2	SNORA5A
RPS26P15	SLC5A2
AC090686.1	AC010768.1
AC092670.1	AL121758.1
PA2G4P6	RNA5SP481
RPL10AP6	LRRC8C-DT
AC097658.2	AC005515.1
HNRNPA1P2	IGF2
RPL3P2	RNA5-8SP6
ANXA8	AL161626.1
HSPA8P5	H2BC9
AC006386.2	SNORD89
HNRNPKP4	H3C11
RPL13AP25	RNA5SP74
PPIAL4C	PRH2
RPS13P2	H2AC4
PPIAP43	NDUFA4L2
AL049873.1	COL5A3
HSP90AA6P	AC024267.4
ANXA8L1	SNORA49
SUMO2P1	RN7SL778P
TPT1P5	RN7SL151P
RPL7P19	C19orf38
PPIAP29	H1-3
AC005000.1	AC024051.8
RPS7P14	AC024051.10
RPL12P8	AC024051.3
RPS27P29	AC024051.7
RPS3AP25	SNORA74A
AP002784.2	RNA5SP162
MTND5P11	H4C4

AL627402.1	AC024051.1
AL121871.1	AL162581.1
TAPT1-AS1	RNU2-1
RPS2P4	AC105036.3
RPS26P39	KIAA1614-AS1
MT-TL1	RP11-180P8.3
RPSAP19	PTGDS
AC078819.1	H4C3
RPS3AP47	AC027281.2
FTH1P3	ARHGAP26-AS1
RPL15P18	H1-4
RPS20P14	H4C6
AL009174.1	SCARNA5
RPL23AP65	KCNK12
AC083873.1	AC026369.2
ACTA1	SNORA80A
Z97353.1	ADGRL3
KRT8P45	CTC-251I16.1
RPL4P3	FAM27B
RPL15P20	RNA5SP225
EEF1A1P29	RPA4
AC092865.1	RNA5SP149
EIF4A1P10	AC087521.3
AC100757.1	HMGCLL1
FTH1P20	RNU1-67P
TMX1	NCAM2
IMP3	PAPPA
AC104619.3	KL
ST13P3	RNA5SP141
AC104257.1	FP236383.5
AC136632.1	RNU4ATAC
AC020898.1	MIR5188
AL139095.2	MAGI1-AS1
EPHA1	RNA5SP506
HLA-G	FZD10
TMSB4XP2	HES5
AC113404.3	AC027514.1
HSP90AB3P	MIR23A
EEF1A1P38	AC024051.9
COX6A1P2	H4C12
DTYMK	RNA5SP429
ALG5	RNA28S5
AL162151.2	B4GALNT1
GAPDHP61	AFF2
RPL7AP11	H2BC14
DPYD	RNA5SP145
KRT8P33	AC005476.2
YWHAZP3	AL157895.2
SCAMP3	AC006449.2
RPS26P28	MIR3648-2
AC073861.1	AC025423.1
RPL23P8	AL355388.1
MORF4L1P1	SNORD15B
NACA3P	ALX4
DENND10P1	CCDC33
PTN	REXO1L2P
AC010468.1	RAG2

UQCRFS1P1	AC092612.1
RPSAP61	PCA3
ALKBH3	RNVU1-27
RPS26P3	DIRC3
AC244034.1	H4C11
CRYZ	ROBO3
AL355802.1	MIR3648-1
FTH1P11	RN7SL752P
PTGES3P3	RN7SL801P
RPSAP5	RN7SL4P
YWHAZP5	FP236383.10
AC112187.1	AL109920.1
AL158206.1	RN7SL5P
CDC42P6	RNA5SP226
RPL21P93	FP236383.12
RBM8B	FP236383.4
XRCC6P2	FP671120.7
GAPDHP65	PDZD4
DBP	IDSP1
TPI1P1	AC010768.2
EZH2	OLFM5P
COQ5	SNORA53
AC092597.1	PRB1
CHCHD1	RNU5A-1
PRCP	H4C5
PGDP1	ERICH6B
FOLR1	TMIGD3
TOP2A	AC090970.1
PIGO	SNORA74B
RPL7AP31	RNA5SP161
SIL1	RNY4P6
AC000089.1	OR10A3
TAGLN2P1	LY86-AS1
C1GALT1C1	AC074135.1
RPL10P12	PRB2
PFN1P1	SCAT2
RTN3P1	AL135938.1
PARP2	RNA5-8SN1
AC107032.1	FP671120.4
CHST12	RNA5-8SN2
HMGN2P46	RNA5-8SN3
GPR89A	PRB4
GLULP4	LINC00901
HLA-DRB6	AL031716.1
TMEM179B	AL160408.1
AC026271.1	RNU4-1
TMSB4XP4	AC079601.1
EIF4BP7	RNY3
ABHD3	HCN2
RPS23P8	BAIAP2L2
EIF4HP1	AC024051.4
MCM2	AC004223.2
DDX50	RNVU1-31
RPSAP18	AC024051.6
RPL26P19	CNTN3
SLC27A4	AC004817.2
TTC3P1	CHRNA4

B3GALT6	RNA5SP502
EIF4BP6	AC024051.2
SETP14	MYH1
AL080243.4	RNA5SP387
AP001324.1	SNORA73B
RFNG	RNY3P1
HYAL2	RNA5SP298
HMGB1P1	DNASE2B
AGAP14P	DLK1
AC022968.1	AC024051.5
RPL12P6	AC024051.11
PWP2	FP671120.2
EEF1B2P3	CR392039.1
FAM171A1	FP236383.9
HNRNPCP2	RP11-216P16.8
AC092115.2	HNRNPA1P9
EIF4BP3	AC019070.2
FKBP9P1	RNA5SP389
FP565260.1	RN7SL753P
MMP15	AC245008.1
EIF4A1P2	AL133368.1
UNC50	DCAF13P2
ST13P5	AL365209.1
MMAB	AC009495.3
HNRNPA3P6	RNA5SP335
EIF4A1P4	AC024051.12
ACTBP2	MIR2278
ZNF226	SMIM9
DPAGT1	AC013403.2
THNSL2	PHOX2A
RDH14	FAM27E2
POLR2I	LINC01391
AL391244.2	IL24
NPM1P39	AC239859.1
TMED1	RP5-1011O1.2
TMEM212	RNY4
RPL12P4	SERTM2
SERBP1P5	RNVU1-2
RPSAP4	AC087239.1
RNF26	AC115989.1
RAC1P2	AC125793.1
MIR22HG	CTD-2306M10.1
TMEM147	RNU4-2
RPL7AP50	NPTX1
ABCB7	FP236383.6
MSH2	SLC45A2
MCM5	VTRNA1-1
ADH1A	RNA5S9
LRRCC1	RNU1-11P
ERO1B	RNA5SP202
AP000936.3	RNU1-3
AL354702.1	RNU1-1
PTK7	RNA5SP370
SIRT3	RNVU1-28
RPL23AP7	CDR1
ALG6	RNVU1-18
EIF3FP3	RNU1-2

CFB	RNU1-4
MSTO1	RNA5S4
MCM6	RNA5S5
STAG3L1	RNA5S6
SMG1P1	RNA5S7
TOPORS	RNA5S3
TMTC4	RNA5S2
TAP2	RNU1-88P
DNAJB9	RNA5S1
EIF2S2P4	RNY1
PMS1	RNA5S12
EXOSC8	RNA5S17
RAMAC	RNA5S16
G6PC3	RNA5S10
SETSIP	RNA5S15
NSMCE3	RNA5S13
TMEM43	RNA5S8
GPR87	RNA5S11
SRD5A3	RNA5S14
AC105250.1	RNVU1-29
H3-5	RNVU1-7
SMIM4	RNU1-27P
LSAMP	RNU1-28P
FSCN1	
COL6A3	
CYP3A5	
NR2C1	
TMEM106C	
FRG1HP	
ANAPC4	
BMI1	
ATP5ME	
NAMPTP1	
ABHD6	
ILKAP	
WFS1	
AC092490.1	
OCLNP1	
ACAD10	
ARAP3	
FBLN1	
MITD1	
CPQ	
BORCS5	
AC115223.1	
LTV1	
SESN1	
DSE	
CARM1	
FKSG70	
TCN1	
PGM5P2	
KLHL42	
PROS1	
FKSG61	
ERVK3-1	
EEF1A1P13	

B3GNT3	
RPS26P31	
AL663070.2	
ANXA2P2	
PPIAP31	
RPS26P47	
RPS7P1	
PPIAP22	
FAM3D	
TP63	
RPL9P7	
GPX1P1	
EEF1A1P11	
MT-TY	
PPIC	
AC004057.1	
ATP1B3	
RPL3P4	
KRT5	
ANKRD66	
RPS3AP6	
RPP25L	
AC024293.1	
EEF1A1P9	
RPS15AP1	
FTH1P7	
RPS26P8	
ADH1B	
UBE2I	
RPS24P8	
AC004552.1	
KRT6B	
GALNT14	
KIT	
ITGA6	
AC092683.1	
HMGB1P5	
FGFR3	
AL049597.1	
TMEM183B	
SLC52A2	
NDUFA12	
H3P47	
MTRNR2L9	
DCAF13	
EEF1A1P25	
H3P6	
HMGB1P6	
RARRES1	
MST1R	
MIR205HG	
ACOT1	
MFSD5	
AC012085.1	
CLCA2	
SERPINB4	
ARSD	

MAD2L1BP	
DPY30	
KRT6A	
RHBDL2	
S100A4	
EMC7	
LPCAT3	
RPL13AP20	
DSG3	
APLP2	
ELMO3	
RPL7P1	
AC009245.1	
H3P16	
SVBP	
THYN1	
IFNGR1	
MRPS21	
RPS7P11	
SRSF2	
AQP3	
ERAL1	
TEX264	
SLC44A3	
FCGRT	
AC106795.1	
PHF14	
CYP2J2	
RPS2P55	
EDEM2	
ITGB6	
CD14	
JPT1	
FTLP3	
ATP10B	
HNRNPA1P7	
SELENOS	
BZW1P2	
UQCC2	
RPL7P9	
RPS26P6	
MINPP1	
PLLP	
TBL2	
PDIA3	
DDAH2	
ITM2C	
RPN2	
PSPC1	
TUBAP2	
NPC1	
EEF1A1P19	
ECI1	
LAMC1	
SLC35B2	
ARMT1	
EXOSC9	

CLDN1	
HIBCH	
FMO5	
RPL7P32	
SNHG6	
PRDX4	
RPS2P46	
PRPF39	
PSME1	
SCYL3	
AC064799.1	
UBBP4	
MANF	
UXS1	
PAPSS2	
SPINT2	
RPL7AP6	
RPL21P28	
TST	
RMDN3	
TUSC3	
METTL17	
SCPEP1	
NOMO2	
PBXIP1	
PYCARD	
CTSC	
CD9	
HLA-A	
PRMT7	
CDS2	
CCDC59	
AC113935.1	
SMC4	
LMAN2	
FANCI	
HNRNPA1P48	
RPL14P1	
AL135745.1	
GAS6	
GPN1	
PGRMC1	
ATRAID	
TSPAN6	
MTCO2P12	
CCT6A	
HEXB	
FKBP9	
TMCO1	
CTSH	
MFSD11	
HLA-F	
ETHE1	
SNX14	
DNAJB11	
PFKM	
ASB3	

HLA-H	
EEF1A1P4	
LAMB3	
BCAP31	
UPK1B	
SLC39A6	
GUSB	
ITM2B	
NDUFAB1	
PPIAL4G	
C1QBP	
PTDSS1	
RUFY2	
KRT8	
KTN1	
AC090498.1	
SDHA	
DSC3	
CXADR	
SNRPD2	
KDSR	
HLA-C	
UFD1	
THOC3	
HADH	
HSP90B1	
ATP6AP1	
HSP90AA1	
RRAGA	
LRRC8D	
ATP6AP2	
PSMD1	
EBPL	
ARMC1	
RPL22P1	
TMEM9	
AGR2	
GBA	
NPTN	
UQCRC1	
PSENEN	
CUEDC1	
CHPF	
MAPKAPK3	
BX679664.3	
PLS1	
CALR	
AC026403.1	
CLK1	
PRSS8	
RCN2	
SNRPA1	
RRM1	
VARS1	
ADAM28	
AC233968.1	
CDH1	

CTNNAL1	
RPL13P12	
BCAT2	
SMARCAD1	
IL10RB	
ACSF2	
CAMK2G	
CHKA	
PLTP	
GFM2	
ARL3	
RPAP2	
SNHG1	
ATP5F1B	
AC233699.1	
RPN1	
RPL10P16	
ACOT2	
GTF2H2B	
GAA	
CD81	
NOMO1	
TWF2	
ABCE1	
GTF2H2C	
CHPT1	
RPL13AP5	
C5orf15	
LUC7L3	
RARS1	
NT5DC1	
SURF1	
PRDX1	
LRG1	
ERMP1	
CKMT1A	
RPS2P5	
ITFG1	
CRYM	
AL592114.1	
CEACAM5	
LRP5	
TAP1	
F11R	
SRSF11	
AKAP1	
HSD17B13	
FAT1	
ANXA4	
HSD17B12	
TXNDC15	
RTN3	
KIFAP3	
CAPG	
DNAJC1	
ARF5	
GCLC	

SDF4	
TMEM59	
KRT19	
CDH3	
HLA-B	
GSTK1	
TM9SF2	
ADRM1	
PCNA	
MAN1B1	
ANKRD36	
WDR61	
RCN1	
MPP7	
PDIA6	
KRT15	
FHL2	
DDOST	
TSPAN13	
AGL	
ITGAV	
SLC35A2	
ASCC3	
TRAPPC3	
GSS	
GRN	
RASA1	
STAM2	
PRELID1	
SMG1P4	
PRKAR2B	
NBEAL1	
ERLEC1	
RPL7AP66	
GNS	
CFH	
P4HB	
CD47	
CTSB	
GLRX5	
RPS21	
AMFR	
MGST1	
B2M	
HACD3	
CLDN4	
BCAP29	
WASHC2C	
APP	
FDFT1	
VPS25	
ATP1A1	
ALDH7A1	
PRXL2A	
ADAM15	
SREK1	
CD151	

TACSTD2	
ARL6IP1	
TOR3A	
MDH1	
MORN2	
ATP2C1	
OS9	
MIPEP	
AKR1A1	
DSG2	
SELENBP1	
ATP5MG	
TSPAN1	
PPIB	
OSBPL3	
TMEM50B	
MBOAT2	
SYNGR2	
S100A6	
WDR33	
SLC44A2	
SOD1	
HNRNPA1P10	
ZMPSTE24	
TK2	
ST14	
TMEM9B	
PTTG1IP	
AC087473.1	
EPCAM	
ANXA1	
MAT2B	
KARS1	
EFTUD2	
TMEM87A	
GPD2	
RPL21P16	
SLC38A10	
PSMB3	
RBM5	
EXOC1	
ARPC3	
MTDH	
RETREG2	
REEP5	
ERBB3	
RBM6	
ATP6V0D1	
MAP3K5	
ALDH2	
TUFM	
RPS26	
PSAP	
ATP5MC3	
CD63	
POR	
UBXN1	

PGD	
ALDH1A1	
DSP	
LRPAP1	
ENO1	
PTPRK	
GALNT7	
TOP2B	
SARAF	
ATXN10	
CCNDBP1	
YWHAB	
HSPA5	
QSOX1	
EEF1A1P5	
SRI	
OCIAD1	
EXOC3	
DDX19A	
EMC4	
SDC1	
CCT5	
HTATIP2	
MMP14	
PKM	
ANXA3	
SLC39A7	
TUBA1A	
ARPC1B	
TAGLN2	
ATP5F1A	
USP48	
PCYOX1	
HMGB1	
PCMTD2	
SRSF3	
CLSTN1	
GSN	
PPP1CA	
DDB1	
NUCB2	
LRIG1	
HNRNPAB	
CLPTM1L	
SMARCA2	
LGALS3BP	
ARPC5	
PYGL	
ANKRD36B	
RTN4	
PSMD4	
NDUFV1	
HNRNPA2B1	
ANPEP	
PLD3	
NRBP1	
IFT57	

CTNNB1	
COPB1	
ACSL5	
DDX5	
ACTG1	
COX4I1	
SRPRA	
GDE1	
SEPHS2	
CAST	
PRKCSH	
LDHB	
HNRNPL	
TSPYL1	
NFE2L1	
EIF3C	
EIF3CL	
SFPQ	
DHCR24	
