# Supplementary material for: Transcriptome of nasopharyngeal samples from COVID-19 patients and a comparative analysis with other SARS-CoV-2 infection models reveal disparate host responses against SARS-CoV-2
Source: J Transl Med. 2021 Jan 7;19:32. doi: 10.1186/s12967-020-02695-0 (PMC7790360; doi:10.1186/s12967-020-02695-0)
Supplement: Supplementary file 11 — Additional file 11. Sources of the data used in this study. [file 12967_2020_2695_MOESM11_ESM.pdf]

Additional file 11: Sources of the data used in this study.

RNA-seq data generated from Nasopharyngeal samples of Bangladeshi COVID-19 patients

Sample ID	Specimen	Virus: GISAID Accession	Human RNA-seq: GEO accession	Total Read No. (R1)	Total Read No. (R2)	Mapped Read No. (Human GRCh38)	Sequencing technology	Virus Assembly method	Lineage	Clade
**S2**	Nasopharyngeal swab	**EPI_ISL_450340**	GSM4667504	10144042	10144042	8354586	Illumina NextSeq500	MEGAHIT v1.1.3	A	S
**S3**	Nasopharyngeal swab	**EPI_ISL_450341**	GSM4667505	9276172	9276172	6542386	Illumina NextSeq500	MEGAHIT v1.1.3	A	S
**S4**	Nasopharyngeal swab	**EPI_ISL_450342**	GSM4667506	9029322	9029322	4788596	Illumina NextSeq500	MEGAHIT v1.1.3	A	S
**S9**	Nasopharyngeal swab	**EPI_ISL_450345**	GSM4667507	8234222	8234222	4170828	Illumina NextSeq500	MEGAHIT v1.1.3	A	S
			**GSE ID**							
			**GSE154244**							

Publicly available data used in this study

**Nasal Control (WT) Healthy Data**							
**GEO Accession**	**SAMPLE-1 (WT)**	**SAMPLE-2 (WT)**	**SAMPLE-3 (WT)**	**SAMPLE-4 (WT)**			
**GSE97668**	GSM2574998	GSM2574999	GSM2575000	GSM2575001	disease status: Non-asthmatic	age: Adult. No Treatment	cell type: Nasal epithelial
Citation: Heymann PW, Nguyen HT, Steinke JW, Turner RB et al. Rhinovirus infection results in stronger and more persistent genomic dysregulation: Evidence for altered innate immune response in asthmatics at baseline, early in infection, and during convalescence. PLoS One 2017;12(5):e0178096. PMID: 28552993							

Infected Cell Data

GEO Accession			GEO: GSM (Sample ID)	
**GSE147507**	ung_GSE147507_GSM44624	Lung	GSM4462415	Lung-Postmortem
	Lung_GSE147507_GSM44624	Lung	GSM4462416	Lung-Postmortem
**GSE150316**	ung_GSE150316_GSM45465	Lung	GSM4546576	Lung-Autopsy
	Lung_GSE150316_GSM45465	Lung	GSM4546581	Lung-Autopsy
	Lung_GSE150316_GSM45465	Lung	GSM4546586	Lung-Autopsy
	Lung_GSE150316_GSM45465	Lung	GSM4546589	Lung-Autopsy
	Lung_GSE150316_GSM45465	Lung	GSM4546596	Lung-Autopsy
**GSE148729**	LU3_GSE148729_GSM4477	Calu3	GSM4477962	Lung-epithelial-cell
	CALU3_GSE148729_GSM4477	Calu3	GSM4477963	Lung-epithelial-cell
**GSE147507**	IBE_GSE147507_GSM4432	NHBE	GSM4432381	Bronchial-Epithelial
	NHBE_GSE147507_GSM4432	NHBE	GSM4432382	Bronchial-Epithelial
	NHBE_GSE147507_GSM4432	NHBE	GSM4432383	Bronchial-Epithelial
