# Supplementary material for: Transcriptome of nasopharyngeal samples from COVID-19 patients and a comparative analysis with other SARS-CoV-2 infection models reveal disparate host responses against SARS-CoV-2
Source: J Transl Med. 2021 Jan 7;19:32. doi: 10.1186/s12967-020-02695-0 (PMC7790360; doi:10.1186/s12967-020-02695-0)
Supplement: Supplementary file 12 — Additional file 12. Per base sequence quality reports of the generated RNA-seq reads of the four COVID-19 infected nasal samples used in this study. [file 12967_2020_2695_MOESM12_ESM.pdf]

Additional file 12 : Per base sequence quality reports of the generated RNA-seq reads of the four COVID-19 infected nasal samples used in this study.

A. Sample S2

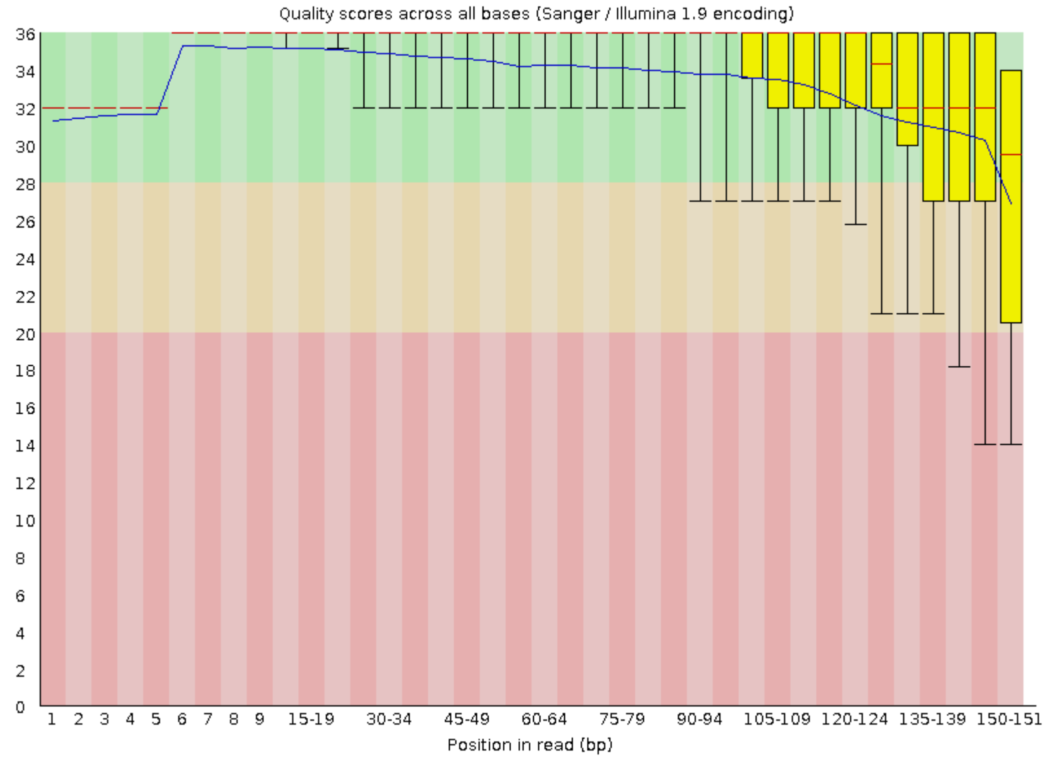

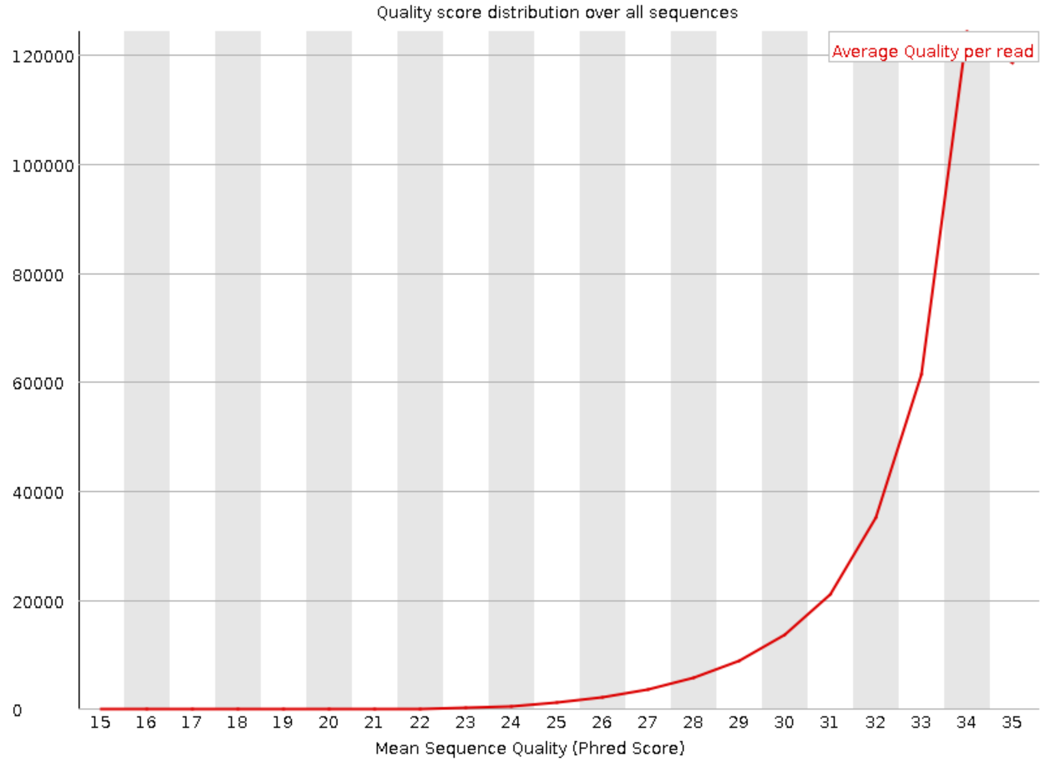

B. Sample S3

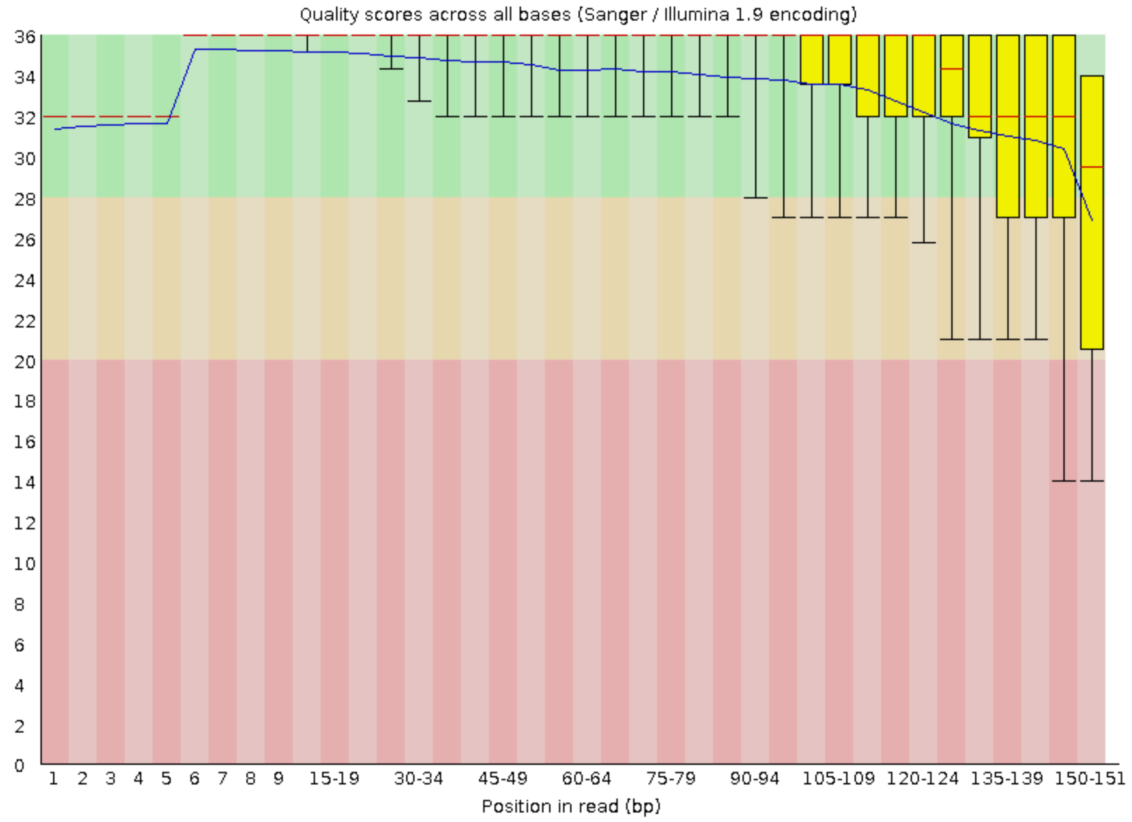

Quality scores across all bases (Sanger / Illumina 1.9 encoding)
36
34
32
30
28
26
24
22
20
18
16
14
12
10
8
6
4
2
0
1 2 3 4 5 6 7 8 9 15-19 30-34 45-49 60-64 75-79 90-94 105-109 120-124 135-139 150-151
Position in read (bp)

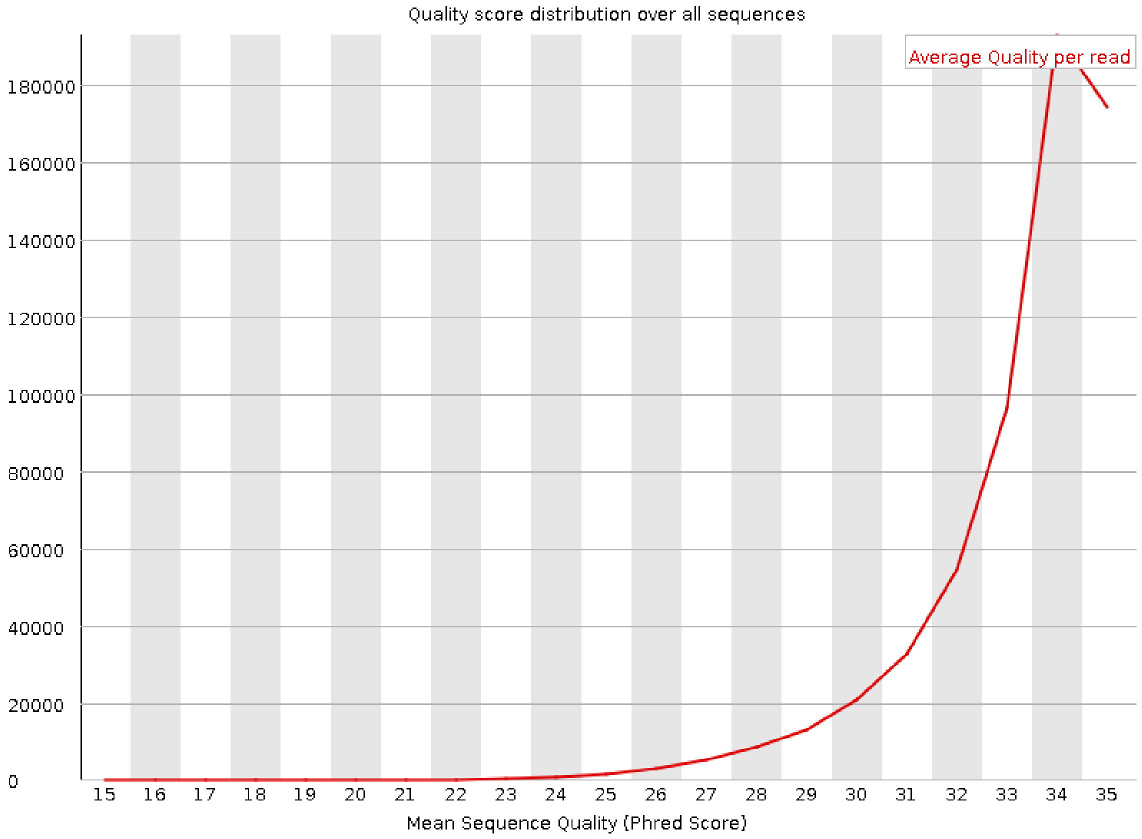

Quality score distribution over all sequences
Average Quality per read
180000
160000
140000
120000
100000
80000
60000
40000
20000
0
15 16 17 18 19 20 21 22 23 24 25 26 27 28 29 30 31 32 33 34 35
Mean Sequence Quality (Phred Score)

C. Sample S4

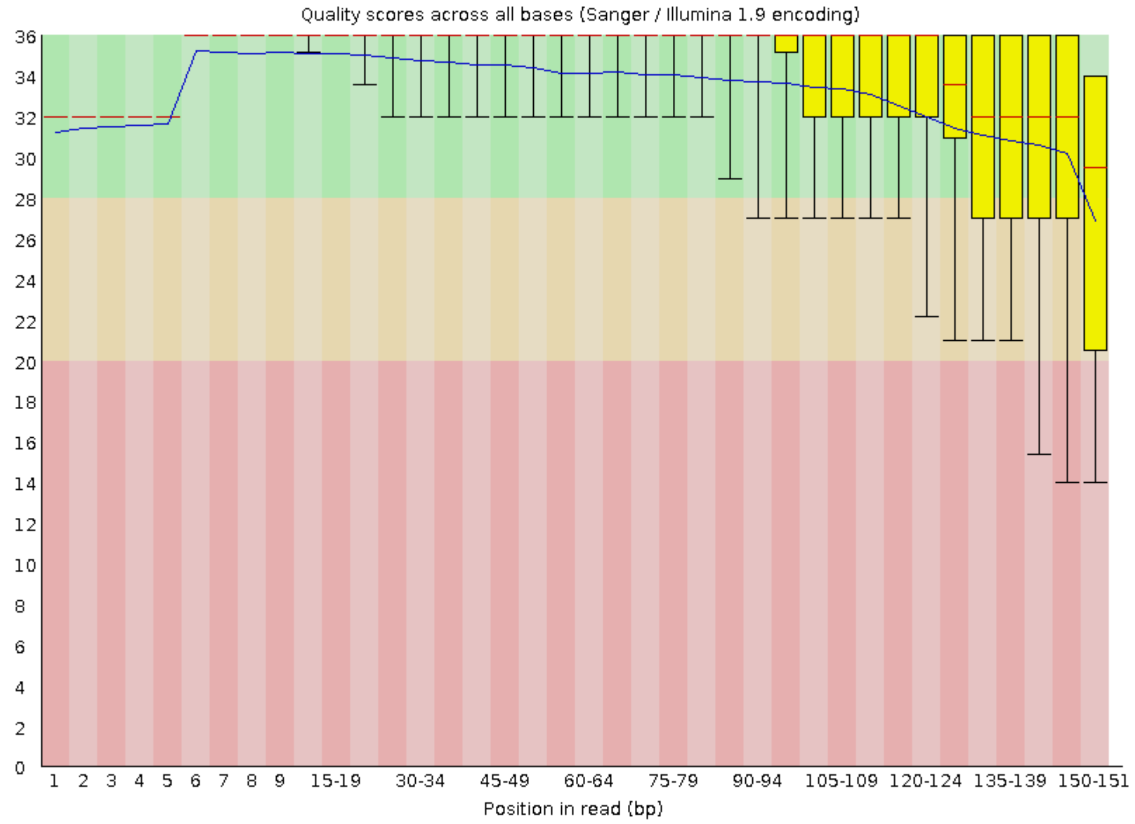

Quality scores across all bases (Sanger / Illumina 1.9 encoding)
36
34
32
30
28
26
24
22
20
18
16
14
12
10
8
6
4
2
0
1 2 3 4 5 6 7 8 9 15-19 30-34 45-49 60-64 75-79 90-94 105-109 120-124 135-139 150-151
Position in read (bp)

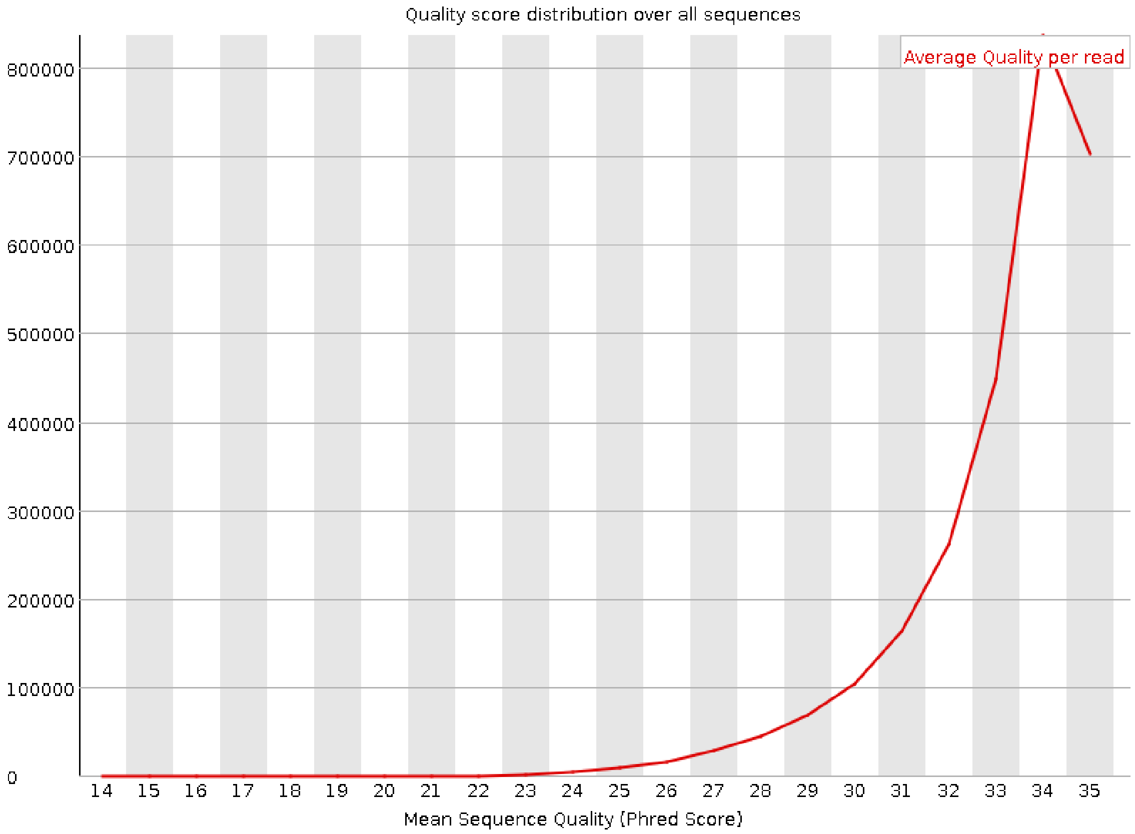

Quality score distribution over all sequences
Average Quality per read
800000
700000
600000
500000
400000
300000
200000
100000
0
14 15 16 17 18 19 20 21 22 23 24 25 26 27 28 29 30 31 32 33 34 35
Mean Sequence Quality (Phred Score)

D. Sample S9

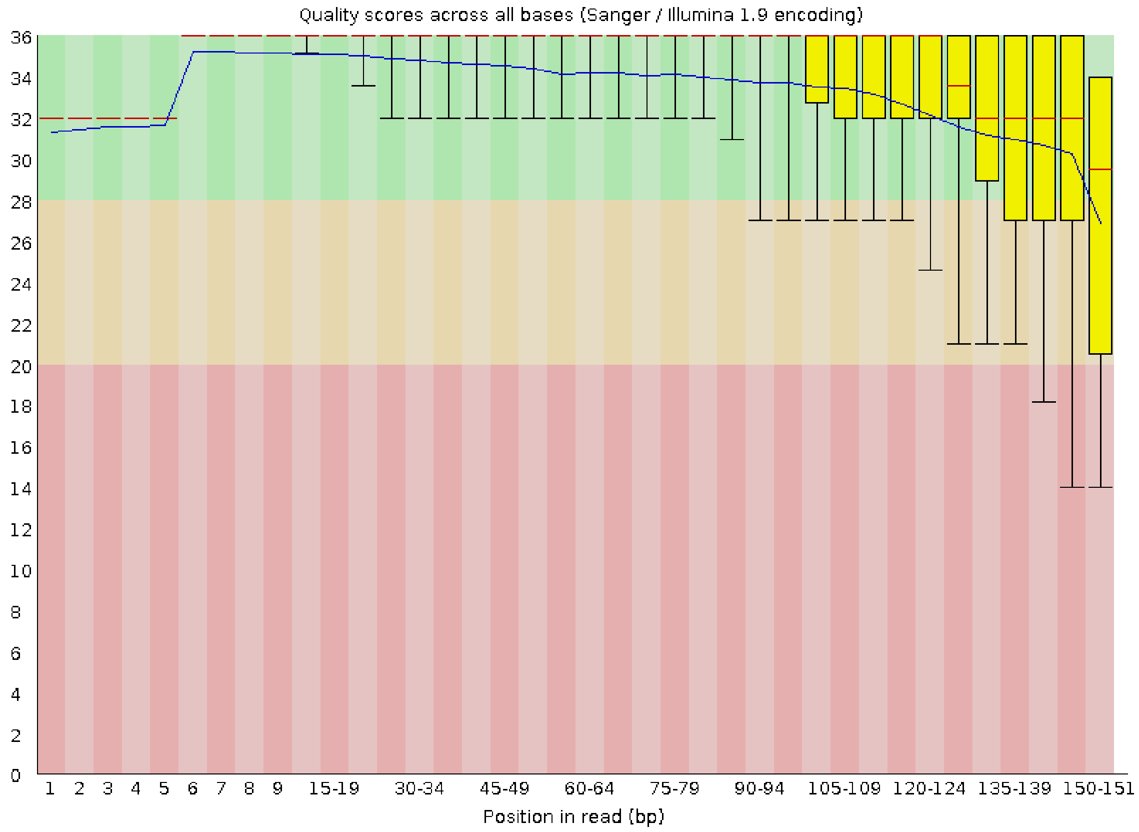
Quality scores across all bases (Sanger / Illumina 1.9 encoding)
0 2 4 6 8 10 12 14 16 18 20 22 24 26 28 30 32 34 36
1 2 3 4 5 6 7 8 9 15-19 30-34 45-49 60-64 75-79 90-94 105-109 120-124 135-139 150-151
Position in read (bp)

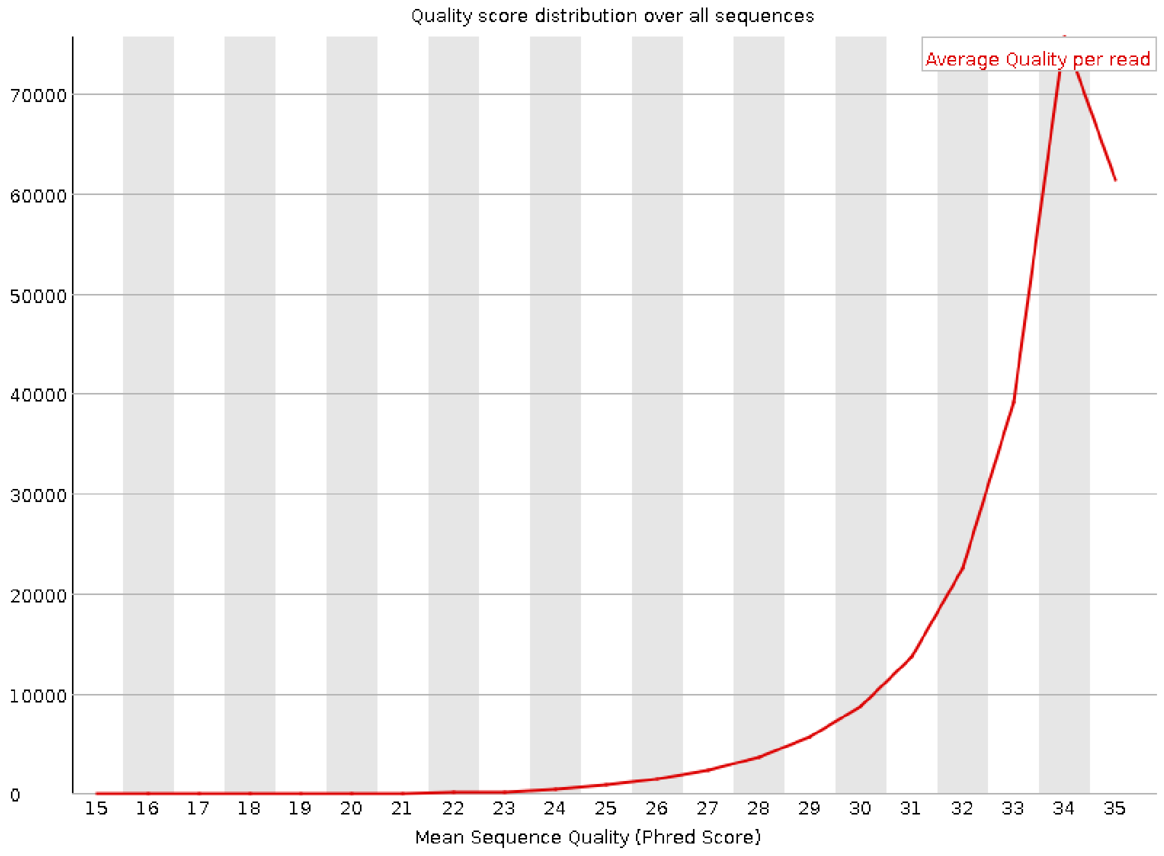
Quality score distribution over all sequences
Average Quality per read
0 10000 20000 30000 40000 50000 60000 70000
15 16 17 18 19 20 21 22 23 24 25 26 27 28 29 30 31 32 33 34 35
Mean Sequence Quality (Phred Score)
